# Determination of Structure and Cytotoxicity of Ten Undescribed Steroidal Glycosides from *Allium cristophii* × *A. macleanii* ‘Globemaster’

**DOI:** 10.3390/molecules28176248

**Published:** 2023-08-25

**Authors:** Tamami Shimazaki, Tomoki Iguchi, Yuna Takahashi, Kie Yamamoto, Naoki Takahashi, Yoshihiro Mimaki

**Affiliations:** Department of Medicinal Pharmacognosy, School of Pharmacy, Tokyo University of Pharmacy and Life Sciences, 1432-1, Horinouchi, Hachioji 192-0392, Tokyo, Japan; y184116@toyaku.ac.jp (T.S.); yuna.disney1124@gmail.com (Y.T.); kiporin2990k@icloud.com (K.Y.); y20612@toyaku.ac.jp (N.T.); mimakiy@toyaku.ac.jp (Y.M.)

**Keywords:** ‘Globemaster’, *Allium cristophii*, *Allium macleanii*, steroidal glycoside, bulb, HL-60 cell, A549 cell, SBC-3 cell, cytotoxicity

## Abstract

‘Globemaster’ is an ornamental hybrid cultivar whose parent plants are *Allium cristophii* and *A. macleanii*. The chemical constituents of ‘Globemaster’ bulbs have not yet been examined; thus, a systematic phytochemical investigation was undertaken herein. A series of chromatographic separations of the MeOH extract of ‘Globemaster’ bulbs afforded 27 steroidal glycosides (**1**–**27**), which are classified into 23 spirostanol glycosides (**1**–**8** and **11**–**25**), two furostanol glycosides (**9** and **26**), a pregnane glycoside (**10**), and a cholestane glycoside (**27**). The structures of the hitherto undescribed compounds (**1**–**10**) were determined from the two-dimensional NMR spectroscopic data and hydrolysis. The cytotoxicity of the isolated compounds (**1**–**27**) toward HL-60 human promyelocytic leukemia cells, A549 human adenocarcinoma lung cancer cells, and SBC-3 human small-cell lung cancer cells was evaluated. Compounds **8**, **22**, **23**, **24**, and **26** exhibited cytotoxicity toward all cell lines in a dose-dependent manner, with IC_50_ values in the 1.3–49 µM range.

## 1. Introduction

The genus *Allium*, belonging to the family Amaryllidaceae, is distributed throughout the Northern Hemisphere and comprises more than 950 species [1]. Although most *Allium* plants are cultivated for ornamental purposes, some species, including *A. cepa* (onion), *A. sativum* (garlic), and *A. chinense* (rakkyo), are used as food sources [1]. Previously, we isolated diverse spirostan-, furostan-, and cholestane-type steroidal glycosides from the following *Allium* species: *A. aflatunense* [2,3], *A. albopilosum* [4], *A. ampeloprasum* [5], *A. chinense* [6], *A. giganteum* [2,7,8], *A. jesdianum* [9], *A. karataviense* [10,11], *A. macleanii* [12], *A. narcissiflorum* [13], *A. ostrowskianum* [4], *A. schubertii* [14,15], *A. senescens* [12], and *A. sphaerosephalon* [16]. These studies indicated that *Allium* species are rich sources of steroidal glycosides. ‘Globemaster’ is a hybrid cultivar of the parent plants *A. cristophii* and *A. macleanii*, which are used as ornamental plants [17]. No phytochemical study or evaluation of the biological activity of the bulbs of ‘Globemaster’ was found in a literature survey.

In 2020, approximately 19 million new cancer cases and 10 million cancer-related deaths were documented globally. Approximately 2.2 million and 470,000 new cases of lung cancer and leukemia and 1.8 million and 310,000 related deaths were estimated in 2020, respectively. Apart from breast cancer, lung cancer is the leading cause of cancer-related mortality in women worldwide [18]. Although anticancer agents such as cisplatin, carboplatin, etoposide, and irinotecan are effective against small-cell lung cancer (SCLC), patients relapse at a relatively high rate [19]. Thus, the development of new anticancer agents is desirable for improving the treatment of cancer patients worldwide.

In this study, we isolated 27 steroidal glycosides (**1**–**27**), including 10 previously undescribed ones (**1**–**10**), from the bulbs of ‘Globemaster’. The structures of **1**–**10** were determined by NMR spectroscopy and hydrolysis. The cytotoxicity of the isolated compounds towards HL-60 human promyelocytic leukemia cells, A549 human adenocarcinoma lung cancer cells, and SBC-3 human small-cell lung cancer cells was evaluated.

## 2. Results

### 2.1. Structure Determination

**Figure 1:**
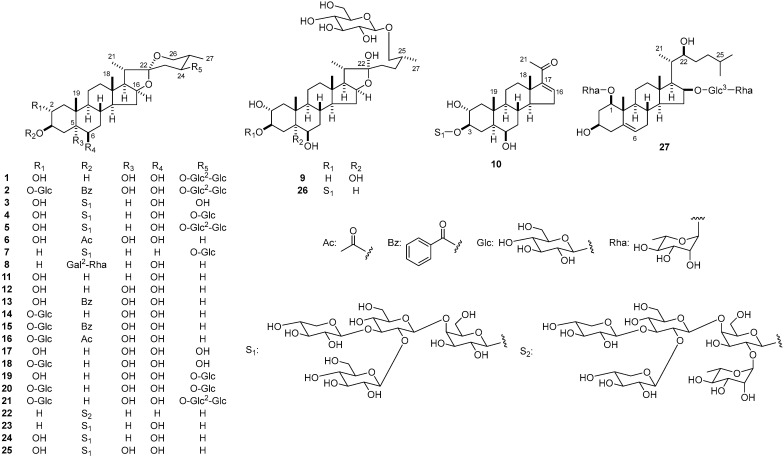
Structures of **1**–**27** from the bulbs of ‘Globemaster’.

Twenty-seven compounds (**1**–**27**) were collected from the bulbs of ‘Globemaster’. Compounds **1**–**27** are classified into 23 spirostanol glycosides (**1**–**8** and **11**–**25**), two furostanol glycosides (**9** and **26**), a pregnane glycoside (**10**), and a cholestane glycoside (**27**). Compounds **11**–**27** were assigned as follows, and select structures are presented in Figure 1: (25*R*)-5α-spirostan-2α,3β,6β-triol (agigenin, **11**) [20], (25*R*)-spirostan-2α,3β,5α,6β-tetrol (alliogenin, **12**) [7], (25*R*)-3β-benzoyloxy-spirostan-2α,5α,6β-triol (karatavigenin, **13**) [21], (25*R*)-3β,5α,6β-trihydroxyspirostan-2α-yl β-d-glucopyranoside (**14**) [7], (25*R*)-3β-benzoyloxy-5α,6β-dihydroxyspirostan-2α-yl β-d-glucopyranoside (**15**) [7], (25*R*)-3β-acetoxy-5α,6β-dihydroxyspirostan-2α-yl β-d-glucopyranoside (**16**) [7], (24*S*,25*S*)-spirostan-2α,3β,5α,6β,24-pentol (**17**) [8], (24*S*,25*S*)-3β,5α,6β,24-tetrahydroxyspirostan-2α-yl β-d-glucopyranoside (**18**) [8], (24*S*,25*S*)-2α,3β,5α,6β-tetrahydroxyspirostan-24-yl β-d-glucopyranoside (**19**) [2], (24*S*,25*S*)-24-[(β-d-glucopyranosyl)oxy]-3β,5α,6β-trihydroxyspirostan-2α-yl β-d-glucopyranoside (**20**) [10], (24*S*,25*S*)-24-[(*O*-β-d-glucopyranosyl-(1→2)-β-d-glucopyranosl)oxy]-3β,5α,6β-trihydroxyspirostan-2α-yl *O*-β-d-glucopyranoside (**21**) [10], (25*R*)-5α-spirostan-3β-yl *O*-α-l-rhamnopyranosyl-(1→2)-*O*-[β-d-xylopyranosyl-(1→2)-*O*-[β-d-xylopyranosyl-(1→3)]-*O*-β-d-glucopyranosyl]-(1→4)-β-d-galactopyranoside (**22**) [12], (25*R*)-6β-hydroxy-5α-spirostan-3β-yl *O*-β-d-glucopyranosyl-(1→2)-*O*-[β-d-xylopyranosyl-(1→3)]-*O*-β-d-glucopyranosyl-(1→4)-β-d-galactopyranoside (**23**) [9], (25*R*)-2α,6β-dihydroxy-5α-spirostan-3β-yl *O*-β-d-glucopyranosyl-(1→2)-*O*-[β-d-xylopyranosyl-(1→3)]-*O*-β-d-glucopyranosyl-(1→4)-β-d-galactopyranoside (**24**) [4], (25*R*)-2α,5α,6β-trihydroxyspirostan-3β-yl *O*-β-d-glucopyranosyl-(1→2)-*O*-[β-d-xylopyranosyl-(1→3)]-*O*-β-d-glucopyranosyl-(1→4)-β-d-galactopyranoside (**25**) [3], (25*R*)-26-[(β-d-glucopyranosyl)oxy]-2α,6β,22α-trihydroxy-5α-furostan-3β-yl *O*-β-d-glucopyranosyl-(1→2)-*O*-[β-d-xylopyranosyl-(1→3)]-*O*-β-d-glucopyranosyl-(1→4)-β-d-galactopyranoside (**26**) [15], and (22*S*)-16β-[(*O*-α-l-rhamnopyranosyl-(1→3)-β-d-glucopyranosyl)oxy]-3β-hydroxycholestan-5-en-1β-yl α-l-rhamnopyranoside (**27**) [4].

**Figure 2:**
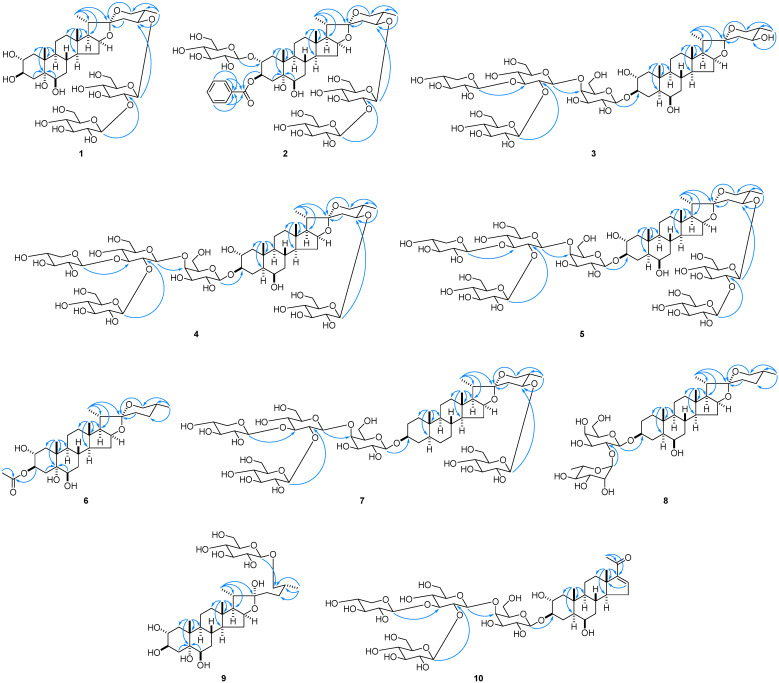
Representative HMBC correlations of **1**–**10**.

Compound **1** was collected as an amorphous solid. The molecular formula of **1** was determined to be C_39_H_64_O_17_ from the high-resolution electrospray ionization time-of-flight mass spectroscopy (HRESITOFMS) (*m*/*z* 827.4035 [M + Na]^+^, calculated for C_39_H_64_NaO_17_: 827.4041) and ^13^C NMR spectral data. The characteristic signals of the following groups were observed in the ^1^H and ^13^C NMR spectra of **1**: two tertiary methyl groups [δ_H_ 1.65 (s, Me-19) and 0.81 (s, Me-18); δ_C_ 18.5 (C-19) and 16.7 (C-18)], two secondary methyl groups [δ_H_ 1.22 (d, *J* = 6.4 Hz, Me-27) and 1.00 (d, *J* = 7.0 Hz, Me-21); δ_C_ 14.8 (C-21) and 13.6 (C-27)], five oxygenated methine groups [δ_H_ 4.83 (m, H-3), 4.50 (q-like, *J* = 7.6 Hz, H-16), 4.45 (m, H-2), 4.22 (m, H-6), and 3.94 (ddd, *J* = 10.7, 10.7, 4.9 Hz, H-24); δ_C_ 81.7 (C-24), 81.5 (C-16), 75.5 (C-6), 73.7 (C-2), and 73.6 (C-3)], an oxygenated methylene group [δ_H_ 3.59 (dd, *J* = 11.4, 4.9 Hz, H-26eq) and 3.49 (dd, *J* = 11.4, 11.4 Hz, H-26ax); δ_C_ 65.2 (C-26)], an acetal carbon [δ_C_ 111.5 (C-22)], an oxygenated quaternary carbon [δ_C_ 75.6 (C-5)], and two anomeric protons/carbons [δ_H_ 5.37 (d, *J* = 7.7 Hz) and 4.90 (d, *J* = 7.7 Hz); δ_C_ 106.1 and 104.2]. Enzymatic hydrolysis of **1** with β-d-glucosidase at 28 °C for nine days yielded **19** and a sugar fraction. The sugar fraction was subjected to direct HPLC analysis, which allowed the identification of d-glucose. The heteronuclear multiple bond correlation (HMBC) spectrum of **1** shows long-range correlations between H-1″ of β-d-glucopyranosyl [Glc (II): δ_H_ 5.37 (d, *J* = 7.7 Hz)] and C-2′ of Glc (I) (δ_C_ 83.7), and between H-1′ of β-d-glucopyranosyl [Glc (I): δ_H_ 4.90 (d, *J* = 7.7 Hz)] and C-24 of the aglycone (δ_C_ 81.7) (Figure 2). The β-anomeric orientations of Glc (I) and Glc (II) were confirmed by their relatively large ^3^*J*_H-1,H-2_ values. Therefore, **1** was determined to be (24*S*,25*S*)-2α,3β,5α,6β-tetrahydroxyspirostan-24-yl *O*-β-d-glucopyranosyl-(1→2)-β-d-glucopyranoside.

Although the ^1^H and ^13^C NMR spectra of **2** (C_52_H_78_O_23_) were similar to those of **21** (C_45_H_74_O_22_), **2** was larger than **21** because of the presence of C_7_H_4_O in the molecular formula of the former. The ^1^H and ^13^C NMR spectra of **2** displayed signals arising from a 1-substituted aromatic group [δ_H_ 8.43 (2H, dd, *J* = 7.8, 1.9 Hz, H-2″″ and H-6″″), 7.45 (1H, m, H-4″″), and 7.44 (2H, m, H-3″″ and H-5″″); δ_C_ 131.9, 130.3, 128.6, 132.9, 128.6, and 130.3 (C-1″″–C-6″″)] and a carbonyl group [δ_C_ 166.8 (C-7″″)]. Analysis of the ^1^H-^1^H correlation spectroscopy (COSY) and heteronuclear single quantum coherence (HSQC) spectra of **2** revealed that **2** has a benzoyl (Bz) moiety. The HMBC spectrum of **2** shows a ^3^*J*_C,H_ correlation from H-3 of the aglycone [δ_H_ 6.29 (ddd, *J* = 11.1, 9.5, 6.2 Hz)] to C-7″″ of Bz (Figure 2). Accordingly, **2** was determined to be (24*S*,25*S*)-3β-benzoyloxy-2α-[(β-d-glucopyranosyl)oxy]-5α,6β-dihydroxyspirostan-24-yl *O*-β-d-glucopyranosyl-(1→2)-β-d-glucopyranoside.

**Figure 3:**
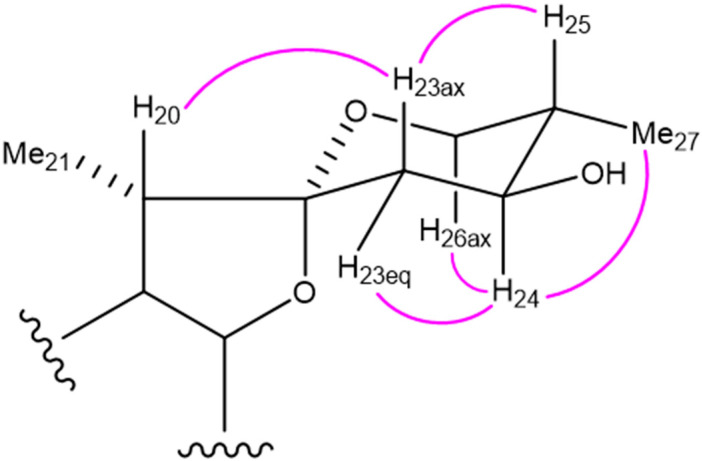
Representative NOE correlations of the F-ring part of **3**.

Analysis of the ^1^H and ^13^C NMR spectra revealed that **3** (C_50_H_82_O_25_) is similar to **24**, except for the F-ring part of the aglycone. The ^1^H-^1^H COSY spectrum of **3** shows spin-coupling correlations between the methine proton [δ_H_ 1.81 (m)] attributable to H-25 and the methyl protons [δ_H_ 1.07 (d, *J* = 6.6 Hz, Me-27)], oxygenated methylene protons [δ_H_ 3.70 (dd, *J* = 10.8, 4.8 Hz, H-26eq) and δ_H_ 3.58 (dd, *J* = 10.8, 10.8 Hz, H-26ax)], and oxygenated methine proton [δ_H_ 3.99 (m, H-24)]. These correlations indicate the presence of a hydroxy group at C-24 of the aglycone. The configuration of C-24 was determined to be *S* based on the nuclear Overhauser effect (NOE) correlation between H-24 and H-26ax in the nuclear Overhauser enhancement spectroscopy (NOESY) spectrum of **3** (Figure 3). The spin-coupling constants of ^3^*J*_H-23ax,H-24_ (12.6 Hz) and ^3^*J*_H-23eq,H-24_ (4.8 Hz) are consistent with the 24*S* configuration. Therefore, **3** was determined to be (24*S*,25*S*)-2α,6β,24-trihydroxy-5α-spirostan-3β-yl *O*-β-d-glucopyranosyl-(1→2)-*O*-[β-d-xylopyranosyl-(1→3)]-*O*-β-d-glucopyranosyl-(1→4)-β-d-galactopyranoside.

The ^1^H and ^13^C NMR spectra of **4** (C_56_H_92_O_30_) were closely related to those of **3**; however, an additional anomeric proton signal [Glc (III): δ_H_ 4.90 (d, *J* = 7.7 Hz, H-1″″′ of Glc (III)] and six carbon signals [δ_C_ 106.3, 75.6, 78.5, 71.6, 77.9, and 62.7 (C-1″″′–C-6″″′ of Glc (III)] corresponding to a terminal β-d-glucopyranosyl unit were detected in the ^1^H and ^13^C NMR spectra of **4**. The HMBC spectrum of **4** shows a long-range correlation between H-1″″′ of Glc (III) and C-24 of the aglycone (δ_C_ 81.3) (Figure 2). Thus, **4** was determined to be (24*S*,25*S*)-24-[(β-d-glucopyranosyl)oxy]-2α,6β-dihydroxy-5α-spirostan-3β-yl *O*-β-d-glucopyranosyl-(1→2)-*O*-[β-d-xylopyranosyl-(1→3)]-*O*-β-d-glucopyranosyl-(1→4)-β-d-galactopyranoside.

The molecular formula of **5** (C_62_H_102_O_35_) was larger than that of **4** (C_56_H_92_O_30_) by C_6_H_10_O_5_. Comparison of the ^1^H and ^13^C NMR spectra of **5** with those of **4** indicated that one more β-d-glucopyranosyl unit [Glc (IV): δ_H_ 5.38 (d, *J* = 7.7 Hz, H-1″″″ of Glc (IV)); δ_C_ 106.0, 76.9, 78.3, 71.4, 77.8, and 62.7 (C-1″″″–C-6″″″ of Glc (IV))] was present in **5**, and the signal of C-2 of the β-d-glucopyranosyl unit [Glc (III): δ_H_ 4.91 (d, *J* = 7.5 Hz, H-1″″′ of Glc (III)); δ_C_ 104.2, 83.6, 78.1, 71.6, 78.4, and 62.5 (C-1″″′–C-6″″′ of Glc (III))] attached to C-24 of the aglycone was shifted downfield by 8.0 ppm. The HMBC spectrum of **5** showed long-range correlations between H-1″″″ of Glc (IV) and C-2″″′ of Glc (III) (δ_C_ 83.6) and between H-1″″′ of Glc (III) and C-24 of the aglycone (δ_C_ 81.7) (Figure 2). Accordingly, **5** was determined to be (24*S*,25*S*)-24-[(*O*-β-d-glucopyranosyl-(1→2)-β-d-glucopyranosyl)oxy]-2α,6β-dihydroxy-5α-spirostan-3β-yl *O*-β-d-glucopyranosyl-(1→2)-*O*-[β-d-xylopyranosyl-(1→3)]-*O*-β-d-glucopyranosyl-(1→4)-β-d-galactopyranoside.

Compound **6** (C_29_H_46_O_7_) was obtained as an amorphous solid. Although the ^1^H and ^13^C NMR spectra of **6** were similar to those of **12**, the signals of the acetyl group [δ_H_ 1.99 (s); δ_C_ 171.0 and 21.4] were detected in the spectra of **6**. A ^3^*J*_C,H_ correlation was observed between H-3 of the aglycone [δ_H_ 6.08 (ddd, *J* = 11.5, 9.4, 5.9 Hz)] and the carbonyl carbon of the acetyl group (δ_C_ 171.0) in the HMBC spectrum of **6** (Figure 2). Therefore, **6** was determined to be (25*R*)-3β-acetoxyspirostan-2α,5α,6β-triol.

The ^1^H and ^13^C NMR spectral data of **7** (C_56_H_92_O_28_) were similar to those of (25*R*)-5α-spirostan-3β-yl *O*-β-d-glucopyranosyl-(1→2)-*O*-[β-d-xylopyranosyl-(1→3)]-*O*-β-d-glucopyranosyl-(1→4)-β-d-galactopyranoside (desgalactotigonin) [22], which was isolated from *Chlorophytum comosum*, except for the signals attributable to the F-ring part of the aglycone. Analysis of the ^1^H-^1^H COSY and HSQC spectra of **7** revealed that the methylene carbon assignable to C-24 (δ_C_ 29.3) of the aglycone in desgalactotigonin was replaced by an oxygenated methine carbon (δ_C_ 81.4) in **7**. Furthermore, an additional signal of the terminal β-d-glucopyranosyl unit [Glc (III): δ_H_ 4.91 (d, *J* = 7.8 Hz, H-1″″′ of Glc (III)); δ_C_ 106.3, 75.6, 78.5, 71.7, 78.0, and 62.8 (C-1″″′–C-6″″′ of Glc (III))] was observed in the ^1^H and ^13^C NMR spectra of **7**. The HMBC spectrum of **7** indicated a long-range correlation between H-1″″′ of Glc (III) and C-24 of the aglycone. The configuration of C-24 was determined to be *S* based on the NOE correlations observed between H-24 [δ_H_ 4.03 (m)] and H-26ax [δ_H_ 3.57 (dd, *J* = 11.4, 11.4 Hz)]. The spin-coupling constants of ^3^*J*_H-23ax,H-24_ (13.2 Hz) and ^3^*J*_H-23eq,H-24_ (4.8 Hz) supported the 24*S* configuration. Thus, **7** was determined to be (24*S*,25*S*)-24-[(β-d-glucopyranosyl)oxy]-5α-spirostan-3β-yl *O*-β-d-glucopyranosyl-(1→2)-*O*-[β-d-xylopyranosyl-(1→3)]-*O*-β-d-glucopyranosyl-(1→4)-β-d-galactopyranoside.

Comparison of the ^1^H and ^13^C NMR spectra of **8** with those of **23** showed that **8** and **23** shared the same aglycone. Analysis of ^1^H-^1^H COSY and HSQC spectra of **8** indicated the presence of a 2-substituted β-d-galactopyranosyl unit [Gal: δ_H_ 4.99 (d, *J* = 7.9 Hz, H-1′ of Gal); δ_C_ 100.5, 76.3, 76.5, 70.7, 76.6, and 62.2 (C-1′–C-6′ of Gal)] and a terminal α-l-rhamnopyranosyl unit [Rha: δ_H_ 6.26 (d, *J* = 1.3 Hz, H-1″ of Rha); δ_C_ 102.0, 72.4, 72.7, 74.3, 69.3, and 18.6 (C-1″–C-6″ of Rha)]. The HMBC spectrum of **8** showed long-range correlations between H-1″ of Rha and C-2′ of Gal, and between H-1′ of Gal and C-3 of the aglycone (δ_C_ 77.9). Accordingly, **8** was determined to be (25*R*)-6β-hydroxy-5α-spirostan-3β-yl *O*-α-l-rhamnopyranosyl-(1→2)-β-d-galactopyranoside.

Compound **9** (C_33_H_56_O_12_) was obtained as an amorphous solid. The ^1^H and ^13^C NMR spectra of **9** displayed signals of the following groups: four steroidal methyl groups [δ_H_ 1.66 (s, Me-19), 1.30 (d, *J* = 7.0 Hz, Me-21), 0.97 (d, *J* = 6.7 Hz, Me-27), and 0.93 (s, Me-18); δ_C_ 18.5 (C-19), 17.4 (C-27), 16.8 (C-18), and 16.3 (C-21)], four oxygenated methine groups [δ_H_ 4.93 (q-like, *J* = 7.3 Hz, H-16), 4.85 (m, H-3), 4.44 (m, H-2), and 4.22 (dd, *J* = 2.7, 2.2 Hz, H-6); δ_C_ 81.1 (C-16), 75.5 (C-6), 73.7 (C-2), and 73.6 (C-3)], an oxygenated methylene group [δ_H_ 3.92 (dd, *J* = 9.6, 7.2 Hz, H-26a) and 3.62 (dd, *J* = 9.6, 5.9 Hz, H-26b); δ_C_ 75.2 (C-26)], an oxygenated quaternary carbon [δ_C_ 75.6 (C-5)], a hemiacetal carbon [δ_C_ 110.6 (C-22)], and an anomeric proton/carbon [δ_H_ 4.81 (d, *J* = 7.8 Hz); δ_C_ 104.8]. The above data and positive Ehrlich’s test results suggest that **9** is a furostanol glycoside. Compound **9** was treated with β-d-glucosidase to afford the corresponding spirostanol glycoside (**12**) and d-glucose. The HMBC spectrum of **9** showed a long-range correlation from H-1′ of the β-d-glucopyranosyl (δ_H_ 4.81) to C-26 of the aglycone (δ_C_ 75.2). In the NOESY spectrum of **9**, NOE correlations were detected between H-20 [δ_H_ 2.23 (m)] and Me-18/H_2_-23 [δ_H_ 2.04 and 1.98 (each m)], indicating the C-20α configuration. Therefore, **9** was determined to be (25*R*)-26-[(β-d-glucopyranosyl)oxy]-furostan-2α,3β,5α,6β,22α-pentol.

Compound **10** (C_44_H_70_O_23_) was obtained as an amorphous solid, the ^1^H and ^13^C NMR spectra of which showed signals of the following groups: three methyl groups [δ_H_ 2.22 (s, Me-21), 1.26 (s, Me-19), and 0.90 (s, Me-18); δ_C_ 27.1 (C-21), 16.9 (C-19), and 16.1 (C-18)], three oxygenated methine groups [δ_H_ 4.01 (m, H-3), 3.99 (m, H-6), and 3.79 (t-like, *J* = 8.8 Hz, H-2); δ_C_ 84.4 (C-3), 70.3 (C-2), and 69.9 (C-6)], and four anomeric protons/carbons [δ_H_ 5.58 (d, *J* = 7.8 Hz), 5.24 (d, *J* = 7.8 Hz), 5.18 (d, *J* = 7.8 Hz), and 4.97 (d, *J* = 7.7 Hz); δ_C_ 104.8, 104.6 (×2), and 103.0]. The existence of an α,β-unsaturated carbonyl group was verified from the infrared (IR) (1700 cm^−1^), ultraviolet (UV) [255 (log *ε* 3.89) and 204 (log *ε* 3.83) nm], and ^13^C NMR [δ_C_ 196.3 (C=O), 155.3 (C), and 144. 7 (CH)] spectra. The HMBC spectrum of **10** indicated a ^2^*J*_C,H_ correlation between the methyl singlet signal assignable to Me-21 (δ_H_ 2.22) and the carbonyl carbon [δ_C_ 196.3 (C-20)] and a ^3^*J*_C,H_ correlation between Me-21 and the olefinic carbon [δ_C_ 155.3 (C-17)]. Furthermore, the olefinic proton [δ_H_ 6.57 (br s, H-16)] exhibited a ^3^*J*_C,H_ correlation with the quaternary carbon [δ_C_ 46.6 (C-13)] bearing an angular methyl group. These spectral data and comparison with those of previously reported compounds [23] and concomitantly isolated compounds, including **3**–**5** and **24**, enabled identification of the aglycone of **10** as 2α,3β,6β-trihydroxy-5α-pregn-16-en-20-one and the sugar moiety as *O*-β-d-glucopyranosyl-(1→2)-*O*-[β-d-xylopyranosyl-(1→3)]-*O*-β-d-glucopyranosyl-(1→4)-β-d-galactopyranosyl. Accordingly, **10** was determined to be 3β-[(*O*-β-d-glucopyranosyl-(1→2)-*O*-[β-d-xylopyranosyl-(1→3)]-*O*-β-d-glucopyranosyl-(1→4)-β-d-galactopyranosyl)oxy]-2α,6β-dihydroxy-5α-pregn-16-en-20-one.

### 2.2. Cytotoxicity of **1**–**27**

**Figure 4:**
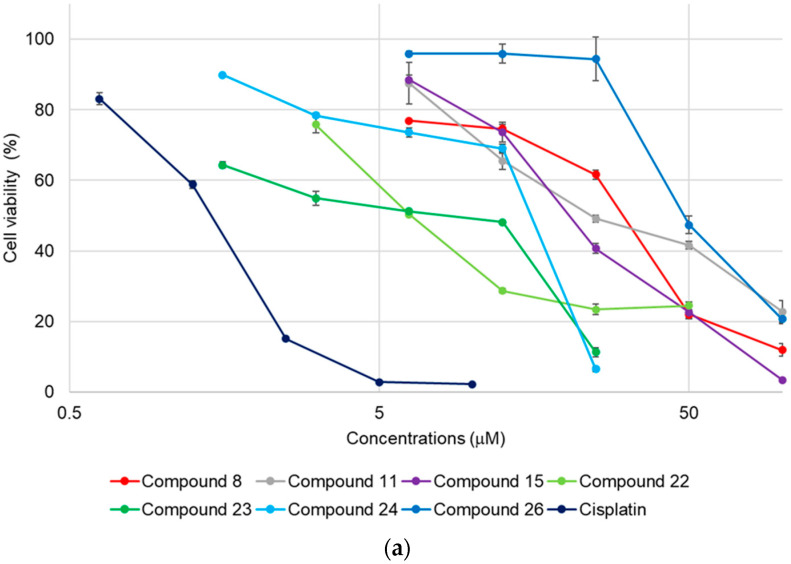

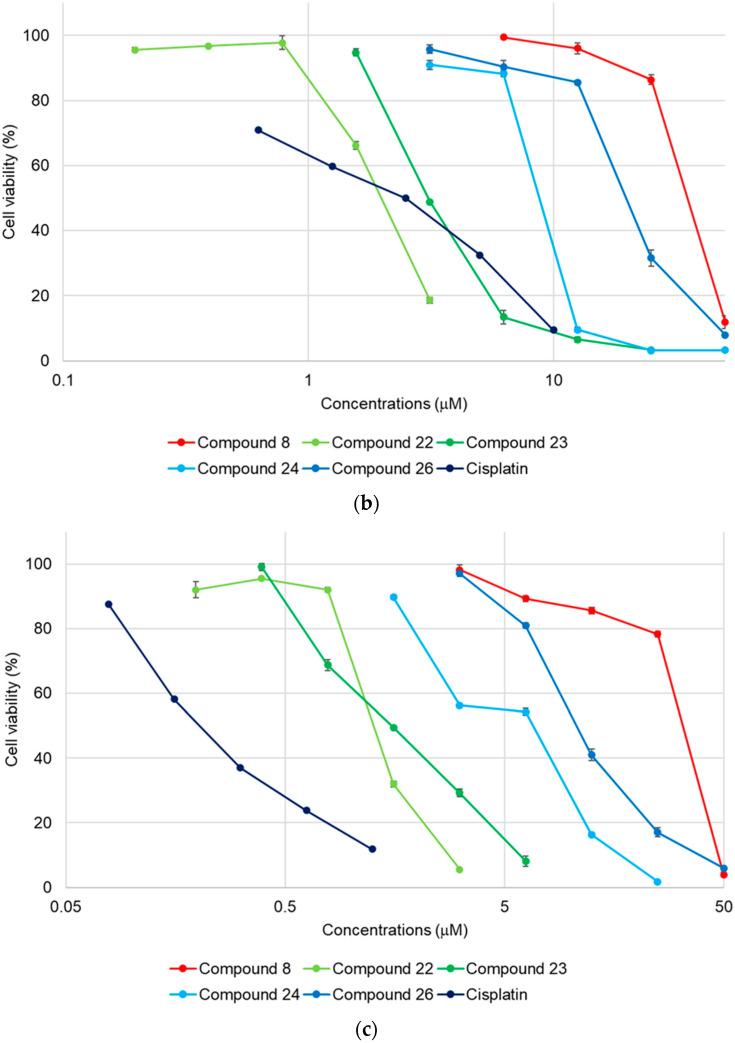
Dose–response curves of **8**, **11**, **15**, **22**–**24**, **26**, and cisplatin. (**a**) HL-60 cells were treated with either **8**, **11**, **15**, **22**–**24**, **26**, or cisplatin for 72 h. (**b**,**c**) A549 cells and SBC-3 cells were independently treated with either **8**, **22**–**24**, **26**, or cisplatin for 72 h. After treatment with each compound, the cell viability was calculated through a modified MTT assay.

The cytotoxicity of **1**–**27** towards HL-60, A549, and SBC-3 cells was evaluated using a modified 3-(4,5-dimethylthiazol-2-yl)-2,5-diphenyltetrazolium bromide (MTT) assay. Compounds **8**, **22**–**24**, and **26** exhibited cytotoxicity toward all cell lines in a dose-dependent manner (Table 1, Figure 4). In particular, **22** and **23** demonstrated cytotoxicity comparable to that of cisplatin.

## 3. Experimental Section

### 3.1. General

Optical rotations were measured with a P-1030 automatic digital polarimeter (JASCO, Tokyo, Japan). IR and UV spectral data were recorded on a Fourier transform infrared (FT-IR) 620 spectrometer (JASCO) and a V-630 UV-vis spectrophotometer (JASCO), respectively. NMR spectral data were collected using a DRX-500 (500 MHz for ^1^H NMR; 125 MHz for ^13^C NMR), DPX-600 (600 MHz for ^1^H NMR; 150 MHz for ^13^C NMR) spectrometer (Bruker, Billerica, MA, USA), or JNM-ECZ600R/M1 (600 MHz for ^1^H NMR; 150 MHz for ^13^C NMR) spectrometer (JEOL, Tokyo, Japan) at 300 K. Chemical shifts are read as δ values. The data of HRESITOFMS were obtained using a Waters Micromass LCT mass spectrometer (Milford, MA, USA). Diaion HP-20 porous polymer polystyrene resin (Mitsubishi-Chemical, Tokyo, Japan), silica gel Chromatorex BW-300 (Fuji-Silysia Chemical, Aichi, Japan), and ODS silica gel COSMOSIL 75C_18_-OPN (Nacalai-Tesque, Kyoto, Japan) were applied for column chromatography (CC). Thin-layer chromatography (TLC) analysis was performed by precoated silica gel 60F_254_ or RP18 F_254_S plates (0.25 mm thick; Merck, Darmstadt, Germany). The sample spots were detected by spraying the TLC plate with H_2_SO_4_/H_2_O (1:9) and then heating. The preparative reverse-phase HPLC system was established from an LC-20 AD pump (Shimadzu, Kyoto, Japan), an RID-10A detector (Shimadzu), a Rheodyne injection port (Thermo Fischer Scientific, Waltham, MA, USA), and a TSKgel ODS-100Z column (10 mm i.d. × 250 mm, 5 μm; Tosoh, Tokyo, Japan). The following reagents and materials were adopted for the cell culture and cytotoxic assay: HL-60 cells (JCRB0085), A549 cells (JCRB0076), and SBC-3 cells (JCRB0818) (Japanese Collection of Research Bioresources Cell Bank; National Institutes of Biomedical Innovation, Health and Nutrition, Osaka, Japan); Roswell Park Memorial Institute (RPMI)-1640 medium, minimum-essential medium (MEM), and cisplatin (Sigma, St. Louis, MO, USA); 0.25% trypsin-ethylenediaminetetraacetic acid (EDTA) solution (Gibco, Gland Island, NY, USA); fetal bovine serum (FBS; NICHIREI BIOSCIENCES, Tokyo, Japan); MTT (DOJINDO, Kumamoto, Japan); penicillin G sodium salt and streptomycin sulfate, and paraformaldehyde and phosphate-buffered saline (PBS) (FUJIFILM Wako Pure Chemical, Osaka, Japan); MCO-170AIC-PJ CO_2_ incubator (PHC, Tokyo, Japan); Countess II FL automated cell counter (Thermo Fisher Scientific); SH-1300 Lab microplate reader (CORONA ELECTRIC, Ibaraki, Japan); 96-well flat-bottomed plates (AGC TECHNO GLASS, Shizuoka, Japan).

### 3.2. Plant Material

The bulbs of ‘Globemaster’ were purchased from Fuji-engei (Okayama, Japan) in 2014. A voucher specimen was kept at the herbarium of the Tokyo University of Pharmacy and Life Sciences (KS-2014-009).

### 3.3. Extraction and Isolation

The bulbs of ‘Globemaster’ (fresh weight, 7.2 kg) were extracted with MeOH (28 L, 60 °C) for 2 h. Then, the solution was evaporated under reduced pressure to obtain MeOH extract (460 g). The MeOH extract was loaded on a Diaion HP-20 porous polymer polystyrene resin column, and successively eluted with MeOH-H_2_O (3:7, *v*/*v*; 18 L), MeOH-H_2_O (1:1, *v*/*v*; 12 L), MeOH (9 L), EtOH (6 L), and EtOAc (6 L). The MeOH-H_2_O (1:1) eluted fraction (10 g) was separated by ODS silica gel CC eluted with MeCN-H_2_O (3:7) to yield five subfractions (Fractions B1–B5). Fraction B1 (3.0 g) was subjected to ODS silica gel CC eluted with MeOH-H_2_O (1:1; 9:11; 2:3) and MeCN-H_2_O (3:7; 1:4; 1:3), and preparative reverse-phase HPLC using MeOH-H_2_O (9:11) and MeCN-H_2_O (1:3) to collect **2** (yield: 4.6 mg; ratio of yield to bulbs of ‘Globemaster’: 6.4 × 10^−5^%), **9** (12 mg; 1.7 × 10^−4^%), **20** (34 mg; 4.7 × 10^−4^%), and **21** (41 mg; 5.7 × 10^−4^%). Fraction B2 (740 mg) was divided by ODS silica gel CC eluted with MeOH-H_2_O (1:1; 9:11; 2:3) and MeCN-H_2_O (1:3; 1:4), and preparative reverse-phase HPLC using MeOH-H_2_O (1:1) and MeCN-H_2_O (1:3) to obtain **1** (73 mg; 1.0 × 10^−3^%), **4** (27 mg; 3.8 × 10^−4^%), **5** (149 mg; 2.1 × 10^−3^%), **10** (2.6 mg; 3.6 × 10^−5^%), **18** (5.8 mg; 8.1 × 10^−5^%), **19** (36 mg; 5.0 × 10^−4^%), **26** (57 mg; 7.9 × 10^−4^%), and **27** (25 mg; 3.5 × 10^−4^%). The MeOH eluted portion (15 g) was chromatographed on a silica gel column eluted with a stepwise gradient mixture of CHCl_3_-MeOH-H_2_O (90:10:1; 40:10:1; 20:10:1; 10:10:1) to afford five subfractions (Fractions C1–C5). Fraction C1 (5.0 g) was separated by silica gel CC eluted with CHCl_3_-MeOH-H_2_O (90:10:1) and EtOAc-MeOH-H_2_O (990:10:1; 190:10:1; 90:10:1), and ODS silica gel CC eluted with MeOH-H_2_O (4:1) and MeCN-H_2_O (13:7; 3:2; 3:7; 1:3) to obtain **6** (2.4 mg; 3.3 × 10^−5^%), **11** (126 mg; 1.8 × 10^−3^%), **12** (14 mg; 1.9 × 10^−4^%), **13** (26 mg; 3.6 × 10^−4^%), and **15** (483 mg; 6.7 × 10^−3^%). Fraction C2 (895 mg) was purified by ODS silica gel CC eluted with MeOH-H_2_O (4:1; 7:3) and MeCN-H_2_O (2:3) to furnish **16** (159 mg; 2.2 × 10^−3^%). Fraction C3 (4.1 g) was separated by silica gel CC eluted with CHCl_3_-MeOH-H_2_O (30:10:1) and ODS silica gel CC eluted with MeOH-H_2_O (4:1) and MeCN-H_2_O (2:3) to afford **8** (6.7 mg; 9.3 × 10^−5^%), **17** (3.7 mg; 5.1 × 10^−5^%), and **23** (23 mg; 3.2 × 10^−4^%). Fraction C4 (8.0 g) was divided by silica gel CC eluted with CHCl_3_-MeOH-H_2_O (30:10:1) and ODS silica gel CC eluted with MeOH-H_2_O (4:1; 7:3; 13:7; 11:9) and MeCN-H_2_O (2:3; 7:13; 3:7) to collect **3** (4.8 mg; 6.7 × 10^−5^%), **7** (5.9 mg; 8.2 × 10^−5^%), **14** (1.6 g; 2.2 × 10^−2^%), **22** (33 mg; 4.6 × 10^−4^%), **24** (144 mg; 2.0 × 10^−3^%), and **25** (6.0 mg; 8.3 × 10^−5^%).

### 3.4. Structure Characterization

#### 3.4.1. Compound **1**

Amorphous solid; [α${]}_{D}^{25}$ −45.6 (MeOH; *c* 0.05); IR (film) ν_max_: 3376 (OH), 2930 (CH); For ^1^H NMR spectral data of the aglycone, see Table 2; For ^1^H NMR spectral data of the sugar moieties, see Table 3; For ^13^C NMR spectral data, see Table 4; HRESITOFMS *m*/*z*: 827.4035 [M + Na]^+^ (calculated for C_39_H_64_NaO_17_, 827.4041). For NMR spectra, see Supplementary Material.

#### 3.4.2. Compound **2**

Amorphous solid; [α${]}_{D}^{25}$ −36.8 (MeOH; *c* 0.05); UV λ_max_ (MeOH) nm (log *ε*): 255 (4.10), 204 (4.00); IR (film) ν_max_: 3376 (OH), 2926 (CH), 1709 (C=O); For ^1^H NMR spectral data of the aglycone, see Table 2; For ^1^H NMR spectral data of the sugar and benzoyl moieties, see Table 3; For ^13^C NMR spectral data, see Table 4; HRESITOFMS *m*/*z*: 1093.4824 [M + Na]^+^ (calculated for C_52_H_78_NaO_23_, 1093.4832).

#### 3.4.3. Compound **3**

Amorphous solid; [α${]}_{D}^{25}$ −32.8 (MeOH; *c* 0.05); IR (film) ν_max_: 3358 (OH), 2926 (CH); For ^1^H NMR spectral data of the aglycone, see Table 2; For ^1^H NMR spectral data of the sugar moieties, see Table 3; For ^13^C NMR spectral data, see Table 4; HRESITOFMS *m*/*z*: 1105.5027 [M + Na]^+^ (calculated for C_50_H_82_NaO_25_, 1105.5043).

#### 3.4.4. Compound **4**

Amorphous solid; [α${]}_{D}^{25}$ −41.2 (MeOH; *c* 0.10); IR (film) ν_max_: 3389 (OH), 2928 (CH); For ^1^H NMR spectral data of the aglycone, see Table 2; For ^1^H NMR spectral data of the sugar moieties, see Table 3; For ^13^C NMR spectral data, see Table 4; HRESITOFMS *m*/*z*: 1267.5569 [M + Na]^+^ (calculated for C_56_H_92_NaO_30_, 1267.5571).

#### 3.4.5. Compound **5**

Amorphous solid; [α${]}_{D}^{25}$ −22.2 (MeOH; *c* 0.10); IR (film) ν_max_: 3375 (OH), 2925 (CH); For ^1^H NMR spectral data of the aglycone, see Table 2; For ^1^H NMR spectral data of the sugar moieties, see Table 3; For ^13^C NMR spectral data, see Table 4; HRESITOFMS *m*/*z*: 1429.6108 [M + Na]^+^ (calculated for C_62_H_102_NaO_35_, 1429.6099).

#### 3.4.6. Compound **6**

Amorphous solid; [α${]}_{D}^{25}$ −15.7 (MeOH; *c* 0.05); IR (film) ν_max_: 3376 (OH), 2926 (CH), 1719 (C=O); For ^1^H NMR spectral data of the aglycone, see Table 2; For ^1^H NMR spectral data of the acetyl moiety, see Table 3; For ^13^C NMR spectral data, see Table 4; HRESITOFMS *m*/*z*: 529.3145 [M + Na]^+^ (calculated for C_29_H_46_NaO_7_, 529.3141).

#### 3.4.7. Compound **7**

Amorphous solid; [α${]}_{D}^{25}$ −24.4 (MeOH; *c* 0.05); IR (film) ν_max_: 3358 (OH), 2926 (CH); For ^1^H NMR spectral data of the aglycone, see Table 2; For ^1^H NMR spectral data of the sugar moieties, see Table 3; For ^13^C NMR spectral data, see Table 4; HRESITOFMS *m*/*z*: 1235.5658 [M + Na]^+^ (calculated for C_56_H_92_NaO_28_, 1235.5673).

#### 3.4.8. Compound **8**

Amorphous solid; [α${]}_{D}^{25}$ −79.2 (MeOH; *c* 0.05); IR (film) ν_max_: 3360 (OH), 2929 (CH); For ^1^H NMR spectral data of the aglycone, see Table 2; For ^1^H NMR spectral data of the sugar moieties, see Table 3; For ^13^C NMR spectral data, see Table 4; HRESITOFMS *m*/*z*: 763.4244 [M + Na]^+^ (calculated for C_39_H_64_NaO_13_, 763.4245).

#### 3.4.9. Compound **9**

Amorphous solid; [α${]}_{D}^{25}$ −44.6 (MeOH; *c* 0.05); IR (film) ν_max_: 3389 (OH), 2929 (CH); For ^1^H NMR spectral data of the aglycone, see Table 2; For ^1^H NMR spectral data of the sugar moiety, see Table 3; For ^13^C NMR spectral data, see Table 4; HRESITOFMS *m*/*z*: 667.3660 [M + Na]^+^ (calculated for C_33_H_56_NaO_12_, 667.3669).

#### 3.4.10. Compound **10**

Amorphous solid; [α${]}_{D}^{25}$ −8.0 (MeOH; *c* 0.05); UV λ_max_ (MeOH) nm (log *ε*): 255 (3.89), 204 (3.83); IR (film) ν_max_: 3375 (OH), 2925 (CH), 1700 (α,β-unsaturated carbonyl group); For ^1^H NMR spectral data of the aglycone, see Table 2; For ^1^H NMR spectral data of the sugar moieties, see Table 3; For ^13^C NMR spectral data, see Table 4; HRESITOFMS *m*/*z*: 989.4198 [M + Na]^+^ (calculated for C_44_H_70_NaO_23_, 989.4206).

#### 3.4.11. Enzymatic Hydrolysis of **1** and **9**

Compounds **1** (15 mg) and **9** (5.0 mg) were independently treated with β-d-glucosidase (EC 232-589-7) in AcOH-AcONa (pH 5.0, 2.0 mL) at 28 °C for nine days (**1**) and 70 h (**9**). The crude hydrolysate of **1** was separated by silica gel CC eluted with EtOAc-MeOH-H_2_O (40:10:1) to obtain **19** (6.2 mg) and the sugar fraction (2.2 mg). The reaction mixture of **9** was purified by silica gel CC eluted with CHCl_3_-MeOH-MeOH (90:10:1) to yield **12** (0.94 mg) and the sugar fraction (0.20 mg). Each sugar fraction was analyzed by HPLC under the following conditions: pump, DP-8020 (Tosoh); detector, Shodex OR2 (Showa-Denko, Tokyo, Japan); column, Capcell Pak NH_2_ (4.6 mm i.d. × 250 mm, 5 μm; Shiseido, Tokyo, Japan); solvent, MeCN-H_2_O (17:3); and flow rate, 1.0 mL/min. d-Glucose was identified by comparing the retention time (*t*_R_) and optical rotation with those of the authentic sample (14.68, positive optical rotation).

### 3.5. Cell Culture and Cytotoxic Activity Assay

HL-60 cells were maintained in RPMI-1640 medium, and A549 and SBC-3 cells were preserved in MEM containing 10% heat-inactivated FBS supplemented with 100 unit/mL penicillin G sodium salt, 100 μg/mL streptomycin sulfate, and l-glutamine, respectively. All cell lines were incubated at 37 °C in a 5% CO_2_/air atmosphere. HL-60, A549, and SBC-3 cells were cultured in a 96-well flat-bottomed plate with cell concentrations of 4 × 10^4^, 1 × 10^4^, and 2 × 10^4^ cells/mL, respectively. After preincubation for 24 h, 4 μL of EtOH-H_2_O (1:1) solution, including each screened sample, was added and incubated for 72 h. The control cells were treated with 4 μL of EtOH-H_2_O (1:1) solution. The cell viability was examined using a modified MTT assay. After 72 h, 10 μL of MTT solution dissolved in PBS at a concentration of 5 mg/mL was added to each well, and the 96-well flat-bottomed plate was further incubated. After 4 h, MTT formazan was dissolved in dimethyl sulfoxide (DMSO), and the absorbance was measured at 550 nm. A dose–response curve was diagramed for **8**, **11**, **15**, **22**–**24**, and **26**, which inhibited cell growth by more than 50% at a sample concentration of 50 μM, and the concentrations at which 50% inhibition (IC_50_) of cell growth occurred were calculated.

## Figures and Tables

**Table 1:** Cytotoxicity of **1**–**27** and cisplatin towards HL-60, A549, and SBC-3 cells ^(1)^.

Compounds	HL-60 Cells	A549 Cells	SBC-3 Cells
	IC_50_ (μM)	IC_50_ (μM)	IC_50_ (μM)
**1**	>50	>50	>50
**2**	>50	>50	>50
**3**	>50	>50	>50
**4**	>50	>50	>50
**5**	>50	>50	>50
**6**	>50	>50	>50
**7**	>50	>50	>50
**8**	30 ± 0.52	35 ± 0.36	33 ± 0.17
**9**	>50	>50	>50
**10**	>50	>50	>50
**11**	25 ± 0.98	>50	>50
**12**	>50	>50	>50
**13**	>50	>50	>50
**14**	>50	>50	>50
**15**	21 ± 0.49	>50	>50
**16**	>50	>50	>50
**17**	>50	>50	>50
**18**	>50	>50	>50
**19**	>50	>50	>50
**20**	>50	>50	>50
**21**	>50	>50	>50
**22**	6.3 ± 0.035	2.0 ± 0.019	1.3 ± 0.010
**23**	8.4 ± 0.13	3.1 ± 0.0067	1.5 ± 0.020
**24**	15 ± 0.18	8.8 ± 0.045	6.6 ± 0.12
**25**	>50	>50	>50
**26**	49 ± 1.6	20 ± 0.48	11 ± 0.29
**27**	>50	>50	>50
Cisplatin	1.3 ± 0.024	2.5 ± 0.058	0.20 ± 0.0033

^(1)^ Data are presented as the mean value ± S.E.M. of three experiments performed in triplicate.

**Table 2:** ^1^H NMR spectral data of the aglycone of **1**–**10** in C_5_D_5_N^ (1)^.

**1**					**2**				
Positions		δ_H_		*J* (Hz)	Positions		δ_H_		*J* (Hz)
1	a	2.45	dd	11.9, 11.9	1	a	2.46	dd	12.2, 12.2
	b	2.15	dd	11.9, 5.0		b	2.36	dd	12.2, 5.5
2		4.45	m		2		4.88	m	
3		4.83	m		3		6.29	ddd	11.1, 9.5, 6.2
4	a	3.05	dd	12.8, 11.9	4	a	2.90	dd	12.9, 11.1
	b	2.41	dd	12.8, 5.6		b	2.57	dd	12.9, 6.2
5		–			5		–		
6		4.22	m		6		4.18	m	
7	a	2.22	m		7	a	2.20	m	
	b	1.88	m			b	1.87	m	
8		2.26	m		8		2.21	m	
9		2.03	m		9		1.95	m	
10		–			10		–		
11	a	1.58	m		11	a	1.41	m	
	b	1.46	m			b	1.33	m	
12	a	1.67	m		12	a	1.64	m	
	b	1.12	ddd	12.7, 12.7, 3.8		b	1.07	m	
13		–			13		–		
14		1.27	m		14		1.22	m	
15	a	2.07	m		15	a	2.07	m	
	b	1.40	m			b	1.39	m	
16		4.50	q-like	7.6	16		4.51	q-like	7.8
17		1.75	dd	7.6, 7.0	17		1.76	dd	7.8, 6.5
18		0.81	s		18		0.81	s	
19		1.65	s		19		1.56	s	
20		1.91	m		20		1.91	m	
21		1.00	d	7.0	21		1.01	d	7.0
22		–			22		–		
23	ax	1.95	dd	12.9, 10.7	23	ax	1.97	m	
	eq	2.61	dd	12.9, 4.9		eq	2.63	dd	12.9, 4.7
24		3.94	ddd	10.7, 10.7, 4.9	24		3.95	m	
25		1.98	m		25		1.99	m	
26	ax	3.49	dd	11.4, 11.4	26	ax	3.50	dd	11.4, 11.4
	eq	3.59	dd	11.4, 4.9		eq	3.61	dd	11.4, 5.0
27		1.22	d	6.4	27		1.23	d	6.5
**3**					**4**				
Positions		δ_H_		*J* (Hz)	Positions		δ_H_		*J* (Hz)
1	a	2.20	dd	12.0, 4.2	1	a	2.18	dd	12.7, 4.3
	b	1.24	m			b	1.22	m	
2		4.10	m		2		4.08	m	
3		4.01	m		3		4.11	m	
4	a	2.38	br dd	12.6, 12.6	4	a	2.37	br dd	12.5, 12.5
	b	2.12	m			b	2.13	m	
5		1.16	m		5		1.14	m	
6		3.96	m		6		3.94	m	
7	a	2.00	m		7	a	2.00	m	
	b	1.15	m			b	1.09	m	
8		2.14	m		8		2.08	m	
9		0.73	ddd	12.0, 12.0, 3.6	9		0.70	m	
10		–			10		–		
11	a	1.53	m		11	a	1.48	m	
	b	1.37	m			b	1.34	m	
12	a	1.66	m		12	a	1.59	m	
	b	1.07	m			b	1.02	m	
13		–			13		–		
14		1.12	m		14		1.07	m	
15	a	2.06	m		15	a	2.11	m	
	b	1.42	m			b	1.36	m	
16		4.55	m		16		4.50	m	
17		1.82	dd	8.4, 6.6	17		1.75	dd	8.0, 6.8
18		0.82	s		18		0.74	s	
19		1.26	s		19		1.24	s	
20		2.03	m		20		1.89	m	
21		1.15	d	6.6	21		1.03	d	6.8
22		–			22		–		
23	ax	1.98	dd	12.6, 12.6	23	ax	1.94	dd	13.0, 13.0
	eq	2.29	dd	12.6, 4.8		eq	2.61	dd	13.0, 4.8
24		3.99	m		24		3.99	m	
25		1.81	m		25		1.87	m	
26	ax	3.58	dd	10.8, 10.8	26	ax	3.54	dd	11.5, 11.5
	eq	3.70	dd	10.8, 4.8		eq	3.61	dd	11.5, 5.0
27		1.07	d	6.6	27		1.12	d	6.4
**5**					**6**				
Positions		δ_H_		*J* (Hz)	Positions		δ_H_		*J* (Hz)
1	a	2.19	dd	12.3, 4.2	1	a	2.50	dd	11.5, 11.5
	b	1.21	m			b	2.18	dd	11.5, 5.5
2		4.09	m		2		4.53	ddd	11.5, 9.4, 5.5
3		4.01	m		3		6.08	ddd	11.5, 9.4, 5.9
4	a	2.38	br dd	12.7, 12.7	4	a	2.83	dd	13.2, 11.5
	b	2.13	m			b	2.38	dd	13.2, 5.9
5		1.14	m		5		–		
6		3.94	m		6		4.21	dd	2.5, 2.5
7	a	1.94	m		7	a	2.26	m	
	b	1.10	m			b	1.93	m	
8		2.10	m		8		2.30	m	
9		0.71	m		9		2.04	m	
10		–			10		–		
11	a	1.49	m		11	a	1.58	m	
	b	1.34	m			b	1.49	m	
12	a	1.60	m		12	a	1.73	m	
	b	1.02	m			b	1.16	m	
13		–			13		–		
14		1.07	m		14		1.31	m	
15	a	2.01	m		15	a	2.14	m	
	b	1.36	m			b	1.46	m	
16		4.49	m		16		4.57	q-like	6.5
17		1.74	dd	8.0, 6.7	17		1.84	dd	8.5, 6.5
18		0.76	s		18		0.90	s	
19		1.25	s		19		1.64	s	
20		1.89	m		20		1.95	m	
21		1.01	d	6.9	21		1.12	d	7.0
22		–			22		–		
23	ax	1.93	m		23	a	1.68	m	
	eq	2.61	dd	12.9, 4.6		b	1.62	m	
24		3.93	m		24	a	1.53	m	
25		1.98	m			b	1.17	m	
26	ax	3.51	dd	11.4, 11.4	25		1.56	m	
	eq	3.61	dd	11.4, 5.6	26	ax	3.47	dd	10.7, 10.7
27		1.23	d	6.4		eq	3.56	dd	10.7, 3.9
					27		0.67	d	6.0
**7**					**8**				
Positions		δ_H_		*J* (Hz)	Positions		δ_H_		*J* (Hz)
1	a	1.50	m		1	a	1.59	m	
	b	0.79	m			b	0.88	m	
2	a	1.58	br d	12.0	2	a	2.14	m	
	b	1.31	m			b	1.94	m	
3		3.89	m		3		4.10	m	
4	a	1.77	br d	13.2	4	a	2.40	br dd	12.6, 12.6
	b	1.35	m			b	2.16	m	
5		0.87	m		5		1.05	m	
6	(2H)	1.09	m		6		3.97	m	
7	a	1.48	m		7	a	2.02	m	
	b	0.77	m			b	1.15	m	
8		1.33	m		8		2.19	m	
9		0.47	ddd	12.0, 12.0, 3.6	9		0.63	m	
10		–			10		–		
11	a	1.37	m		11	a	1.47	m	
	b	1.14	m			b	1.40	m	
12	a	1.60	m		12	a	1.70	m	
	b	1.01	m			b	1.11	m	
13		–			13		–		
14		1.00	m		14		1.13	m	
15	a	1.98	m		15	a	2.07	m	
	b	1.34	m			b	1.42	m	
16		4.51	m		16		4.54	m	
17		1.73	dd	8.4, 6.6	17		1.82	dd	8.4, 6.6
18		0.73	s		18		0.83	s	
19		0.62	s		19		1.36	s	
20		1.94	m		20		1.94	m	
21		1.05	d	7.2	21		1.13	d	6.9
22		–			22		–		
23	ax	1.96	dd	13.2, 13.2	23	a	1.66	m	
	eq	2.67	dd	13.2, 4.8		b	1.58	m	
24		4.03	m		24	a	1.53	m	
25		1.90	m			b	1.50	m	
26	ax	3.57	dd	11.4, 11.4	25		1.54	m	
	eq	3.63	dd	11.4, 5.4	26	ax	3.48	dd	10.7, 10.7
27		1.14	d	6.6		eq	3.57	dd	10.7, 3.8
					27		0.67	d	6.0
**9**					**10**				
Positions		δ_H_		*J* (Hz)	Positions		δ_H_		*J* (Hz)
1	a	2.45	dd	12.1, 12.1	1	a	2.13	m	
	b	2.16	dd	12.1, 5.3		b	1.20	m	
2		4.44	m		2		3.79	t-like	8.8
3		4.85	m		3		4.01	m	
4	a	3.06	dd	13.3, 11.8	4	a	2.38	m	
	b	2.41	dd	13.3, 5.6		b	2.15	m	
5		–			5		1.14	m	
6		4.22	dd	2.7, 2.2	6		3.99	m	
7	a	2.21	m		7	a	2.00	m	
	b	1.91	m			b	1.22	m	
8		2.31	m		8		2.13	m	
9		2.03	m		9		0.78	m	
10		–			10		–		
11	a	1.59	m		11	a	1.57	m	
	b	1.51	m			b	1.48	m	
12	a	1.78	br d	12.6	12	a	2.56	m	
	b	1.19	ddd	12.6, 12.6, 3.9		b	1.37	m	
13		–			13		–		
14		1.31	m		14		1.38	m	
15	a	2.09	m		15	a	2.14	m	
	b	1.45	m			b	1.86	br dd	16.4, 12.5
16		4.93	q-like	7.3	16		6.57	br s	
17		1.95	dd	8.7, 7.3	17		–		
18		0.93	s		18		0.90	s	
19		1.66	s		19		1.26	s	
20		2.23	m		20		–		
21		1.30	d	7.0	21		2.22	s	
22		–							
23	a	2.04	m						
	b	1.98	m						
24	a	2.01	m						
	b	1.66	m						
25		1.90	m						
26	a	3.92	dd	9.6, 7.2					
	b	3.62	dd	9.6, 5.9					
27		0.97	d	6.7					

^(1)^ 500 MHz for **2**, **5**, **6**, **8**, and **9**. 600 MHz for **1**, **3**, **4**, **7**, and **10**.

**Table 3:** ^1^H NMR spectral data of the sugar and acyl moieties of **1**–**10** in C_5_D_5_N ^(1)^.

**1**					**2**				
Positions		δ_H_		*J* (Hz)	Positions		δ_H_		*J* (Hz)
Glc (I) 1′		4.90	d	7.7	Glc (I) 1′		5.22	d	7.8
2′		4.22	dd	9.0, 7.7	2′		3.96	dd	9.0, 7.8
3′		4.24	dd	9.0, 9.0	3′		4.30	dd	9.0, 9.0
4′		4.29	dd	9.0, 9.0	4′		4.20	dd	9.0, 9.0
5′		3.90	m		5′		3.89	m	
6′	a	4.44	m		6′	a	4.40	dd	11.6, 2.7
	b	4.39	dd	11.6, 4.5		b	4.31	dd	11.6, 4.6
Glc (II) 1″		5.37	d	7.7	Glc (II) 1″		4.91	d	7.7
2″		4.11	dd	8.8, 7.7	2″		4.22	dd	8.9, 7.7
3″		4.30	dd	9.1, 8.8	3″		4.25	dd	8.9, 8.9
4″		4.25	dd	9.1, 9.1	4″		4.28	m	
5″		3.77	m		5″		3.91	m	
6″	a	4.46	m		6″	a	4.45	dd	11.7, 2.9
	b	4.34	dd	11.7, 5.1		b	4.38	dd	11.7, 5.0
					Glc (III) 1″′		5.37	d	7.7
					2″′		4.11	dd	8.9, 7.7
					3″′		4.29	dd	8.9, 8.9
					4″′		4.23	dd	8.9, 8.9
					5″′		3.78	m	
					6″′	a	4.46	dd	11.8, 2.5
						b	4.34	dd	11.8, 5.3
					Bz 1″″		–		
					2″″		8.43	dd	7.8, 1.9
					3″″		7.44	m	
					4″″		7.45	m	
					5″″		7.44	m	
					6″″		8.43	dd	7.8, 1.9
					7″″		–		
**3**					**4**				
Positions		δ_H_		*J* (Hz)	Positions		δ_H_		*J* (Hz)
Gal 1′		4.96	d	7.8	Gal 1′		4.97	d	7.7
2′		4.53	m		2′		4.52	dd	9.1, 7.7
3′		4.12	m		3′		4.13	dd	9.1, 3.4
4′		4.58	br s		4′		4.58	br d	3.4
5′		4.03	m		5′		4.04	m	
6′	a	4.60	dd	10.8, 8.4	6′	a	4.59	dd	10.7, 8.8
	b	4.20	dd	10.8, 5.4		b	4.20	m	
Glc (I) 1″		5.18	d	7.8	Glc (I) 1″		5.18	d	7.9
2″		4.32	dd	8.4, 7.8	2″		4.31	dd	8.7, 7.9
3″		4.13	m		3″		4.12	dd	8.7, 8.7
4″		3.79	dd	8.4, 8.4	4″		3.79	dd	8.7, 8.7
5″		3.84	m		5″		3.84	m	
6″	a	4.48	dd	11.4, 1.8	6″	a	4.37	dd	11.8, 5.1
	b	4.05	m			b	4.03	dd	11.8, 3.5
Glc (II) 1″′		5.57	d	7.8	Glc (II) 1″′		5.58	d	7.9
2″′		4.03	dd	9.0, 7.8	2″′		4.02	dd	8.9, 7.9
3″′		4.16	dd	9.0, 9.0	3″′		4.17	dd	8.9, 8.9
4″′		4.07	dd	9.0, 9.0	4″′		4.09	dd	8.9, 8.9
5″′		3.90	m		5″′		3.89	m	
6″′	a	4.52	dd	12.0, 1.8	6″′	a	4.48	dd	12.1, 2.0
	b	4.41	dd	12.0, 5.4		b	4.41	dd	12.1, 5.4
Xyl 1″″		5.24	d	7.8	Xyl 1″″		5.24	d	7.8
2″″		3.95	dd	8.4, 7.8	2″″		3.95	dd	8.7, 7.8
3″″		4.10	m		3″″		4.11	dd	8.7, 8.7
4″″		4.11	m		4″″		4.14	m	
5″″	a	4.22	m		5″″	a	4.21	dd	10.8, 4.3
	b	3.66	dd	10.2, 10.2		b	3.66	dd	10.8, 10.8
					Glc (III) 1″″′		4.90	d	7.7
					2″″′		4.06	dd	8.8, 7.7
					3″″′		4.23	dd	8.8, 8.8
					4″″′		4.26	dd	8.8, 8.8
					5″″′		3.86	m	
					6″″′	a	4.52	dd	12.1, 2.0
						b	4.39	dd	12.1, 5.1
**5**					**6**				
Positions		δ_H_		*J* (Hz)	Positions		δ_H_		*J* (Hz)
Gal 1′		4.97	d	7.8	Ac	1.99	s		
2′		4.55	dd	9.0, 7.8					
3′		4.03	m						
4′		4.59	br d	2.9					
5′		3.85	m						
6′	a	4.61	dd	10.6, 9.0					
	b	4.20	br d	10.6					
Glc (I) 1″		5.20	d	7.9					
2″		4.34	dd	8.8, 7.9					
3″		4.13	dd	8.8, 8.8					
4″		3.80	dd	8.8, 8.8					
5″		3.84	m						
6″	a	4.49	dd	11.3, 2.2					
	b	4.03	m						
Glc (II) 1″′		5.59	d	7.5					
2″′		4.04	dd	9.0, 7.5					
3″′		4.15	dd	9.0, 9.0					
4″′		4.07	dd	9.0, 9.0					
5″′		3.90	m						
6″′	a	4.54	dd	12.3, 2.3					
	b	4.41	dd	12.3, 5.7					
Xyl 1″″		5.25	d	7.8					
2″″		3.95	dd	8.2, 7.8					
3″″		4.10	m						
4″″		4.11	m						
5″″	a	4.21	dd	10.9, 4.5					
	b	3.66	dd	10.9, 10.9					
Glc (III) 1″″′		4.91	d	7.5					
2″″′		4.23	dd	9.2, 7.5					
3″″′		4.28	dd	9.2, 9.2					
4″″′		4.27	m						
5″″′		3.92	m						
6″″′	a	4.46	dd	11.9, 2.5					
	b	4.32	dd	11.9, 4.5					
Glc (IV) 1″″″		5.38	d	7.7					
2″″″		4.10	dd	8.8, 7.7					
3″″″		4.24	dd	8.8, 8.8					
4″″″		4.22	dd	8.8, 8.8					
5″″″		3.79	m						
6″″″	a	4.45	br d	11.9					
	b	4.37	dd	11.9, 4.9					
**7**					**8**				
Positions		δ_H_		*J* (Hz)	Positions		δ_H_		*J* (Hz)
Gal 1′		4.89	d	7.8	Gal 1′		4.99	d	7.9
2′		4.40	dd	9.0, 7.8	2′		4.62	dd	9.5, 7.9
3′		4.11	m		3′		4.24	dd	9.5, 3.5
4′		4.59	br d	3.0	4′		4.53	br d	3.5
5′		4.04	m		5′		4.05	m	
6′	a	4.70	dd	10.2, 9.0	6′	a	4.55	m	
	b	4.21	m			b	4.43	dd	11.4, 5.3
Glc (I) 1″		5.16	d	7.8	Rha 1″		6.26	d	1.3
2″		4.37	dd	9.0, 7.8	2″		4.80	dd	3.4, 1.3
3″		4.14	dd	9.0, 9.0	3″		4.66	dd	9.4, 3.4
4″		3.80	dd	9.0, 9.0	4″		4.28	dd	9.4, 9.4
5″		3.90	m		5″		4.98	m	
6″	a	4.50	m		6″		1.67	d	6.2
	b	4.04	m						
Glc (II) 1″′		5.55	d	7.8					
2″′		4.02	m						
3″′		4.12	m						
4″′		4.20	dd	9.0, 9.0					
5″′		3.89	m						
6″′	a	4.53	dd	12.0, 1.8					
	b	4.38	m						
Xyl 1″″		5.22	d	7.8					
2″″		3.96	dd	8.4, 7.8					
3″″		4.10	m						
4″″		4.12	m						
5″″	a	4.22	m						
	b	3.67	dd	11.4, 11.4					
Glc (III) 1″″′		4.91	d	7.8					
2″″′		4.04	dd	9.0, 7.8					
3″″′		4.21	m						
4″″′		4.25	dd	9.0, 9.0					
5″″′		3.85	m						
6″″′	a	4.48	dd	12.0, 2.4					
	b	4.35	dd	12.0, 5.4					
**9**					**10**				
Positions		δ_H_		*J* (Hz)	Positions		δ_H_		*J* (Hz)
Glc 1′		4.81	d	7.8	Gal 1′		4.97	d	7.7
2′		4.02	dd	8.6, 7.8	2′		4.53	dd	8.8, 7.7
3′		4.26	dd	8.6, 8.6	3′		4.14	m	
4′		4.23	dd	8.6, 8.6	4′		4.58	br d	3.4
5′		3.93	m		5′		4.00	m	
6′	a	4.54	dd	11.8, 2.4	6′	a	4.60	dd	10.7, 8.9
	b	4.39	dd	11.8, 5.3		b	4.20	m	
					Glc (I) 1″		5.18	d	7.8
					2″		4.32	dd	8.5, 7.8
					3″		4.13	m	
					4″		4.07	dd	9.1, 9.1
					5″		3.84	m	
					6″	a	4.49	br d	11.3
						b	4.20	m	
					Glc (II) 1″′		5.58	d	7.8
					2″′		4.03	dd	9.0, 7.8
					3″′		4.17	dd	9.0, 9.0
					4″′		4.12	m	
					5″′		3.90	m	
					6″′	a	4.52	dd	12.1, 3.8
						b	4.41	dd	12.1, 5.3
					Xyl 1″″		5.24	d	7.8
					2″″		3.95	dd	8.3, 7.8
					3″″		4.11	m	
					4″″		4.08	m	
					5″″	a	4.21	m	
						b	3.66	dd	11.3, 11.3

^(1)^ 500 MHz for **2**, **5**, **6**, **8**, and **9**. 600 MHz for **1**, **3**, **4**, **7**, and **10**.

**Table 4:** ^13^C NMR spectral data of **1**–**10** in C_5_D_5_N ^(1)^.

**Positions**	**1**	**2**	**3**	**4**	**5**
	**δ**_**C**_	**δ**_**C**_	**δ**_**C**_	**δ**_**C**_	**δ**_**C**_
1	42.2	38.9	47.1	47.1	47.1
2	73.7	77.7	70.5	70.4	70.5
3	73.6	76.4	84.6	84.5	84.5
4	41.1	37.8	31.9	31.8	31.9
5	75.6	75.0	47.8	47.7	47.8
6	75.5	74.9	69.9	69.9	69.9
7	35.7	35.6	40.7	40.5	40.6
8	30.1	30.0	29.9	29.8	29.9
9	45.8	45.5	54.5	54.4	54.4
10	40.9	40.4	37.0	36.9	37.0
11	21.6	21.4	21.3	21.2	21.3
12	40.4	40.2	40.0	39.9	40.0
13	40.9	40.9	40.8	40.7	40.7
14	56.3	56.1	56.2	56.1	56.1
15	32.1	32.1	32.1	32.0	32.0
16	81.5	81.5	81.4	81.4	81.4
17	62.6	62.6	62.6	62.4	62.5
18	16.7	16.6	16.5	16.5	16.5
19	18.5	17.9	17.1	17.1	17.1
20	42.1	42.1	42.2	42.0	42.1
21	14.8	14.8	14.9	14.8	148.0
22	111.5	111.5	111.8	111.5	111.5
23	40.6	40.6	41.8	40.7	40.6
24	81.7	81.7	70.5	81.3	81.7
25	38.0	38.0	39.9	38.1	38.0
26	65.2	65.2	65.3	65.0	65.2
27	13.6	13.6	13.6	13.4	13.6
	Glc (I)	Glc (I)	Gal	Gal	Gal
1′	104.2	103.3	103.1	103.0	103.0
2′	83.7	75.3	72.5	72.5	72.5
3′	78.0	78.5	75.5	75.4	75.5
4′	71.6	71.6	79.5	79.6	79.4
5′	78.4	78.4	75.7	75.6	75.7
6′	62.7	62.8	60.6	60.6	60.6
	Glc (II)	Glc (II)	Glc (I)	Glc (I)	Glc (I)
1″	106.1	104.2	104.6	104.6	104.6
2″	76.9	83.7	81.2	81.1	81.2
3″	78.2	78.0	87.1	87.0	87.0
4″	71.3	71.6	70.3	70.2	70.3
5″	77.8	78.4	77.5	77.5	77.5
6″	62.5	62.7	62.8	62.8	62.8
		Glc (III)	Glc (II)	Glc (II)	Glc (II)
1″′		106.1	104.7	104.6	104.7
2″′		76.9	76.0	75.9	76.0
3″′		78.2	78.1	78.0	78.0
4″′		71.3	71.3	71.2	71.3
5″′		77.8	78.4	78.3	78.3
6″′		62.5	62.6	62.5	62.6
		Bz	Xyl	Xyl	Xyl
1″″		131.9	104.9	104.8	104.9
2″″		130.3	75.1	75.0	75.1
3″″		128.6	78.6	78.6	78.6
4″″		132.9	70.8	70.8	70.7
5″″		128.6	67.2	67.2	67.2
6″″		130.3			
7″″		166.8			
				Glc (III)	Glc (III)
1″″′				106.3	104.2
2″″′				75.6	83.6
3″″′				78.5	78.1
4″″′				71.6	71.6
5″″′				77.9	78.4
6″″′				62.7	62.5
					Glc (IV)
1″″″					106.0
2″″″					76.9
3″″″					78.3
4″″″					71.4
5″″″					77.8
6″″″					62.7
**Positions**	**6**	**7**	**8**	**9**	**10**
	**δ_C_**	**δ_C_**	**δ_C_**	**δ_C_**	**δ_C_**
1	42.4	37.1	38.8	42.2	46.9
2	69.7	29.9	30.1	73.7	70.3
3	78.2	77.4	77.9	73.6	84.4
4	37.7	34.8	32.5	41.1	31.9
5	75.3	44.6	47.8	75.6	48.0
6	75.1	28.9	70.8	75.5	69.9
7	35.8	32.3	40.7	35.8	40.2
8	30.1	35.2	30.6	30.1	28.6
9	45.7	54.3	54.6	45.9	55.0
10	40.6	35.8	36.1	41.3	37.1
11	21.5	21.2	21.2	21.7	21.3
12	40.3	40.0	40.2	40.5	35.2
13	41.0	40.6	40.8	40.9	46.6
14	56.2	56.4	56.3	56.2	56.1
15	32.3	31.9	32.2	32.5	32.3
16	81.2	81.5	81.1	81.1	144.7
17	63.1	62.5	63.0	63.9	155.3
18	16.7	16.5	16.5	16.8	16.1
19	18.2	12.2	16.0	18.5	16.9
20	42.0	42.0	41.9	40.6	196.3
21	15.0	14.8	15.0	16.3	27.1
22	109.2	111.6	109.2	110.6	
23	31.8	40.8	31.7	37.1	
24	29.2	81.4	29.2	28.3	
25	30.5	38.1	30.5	34.2	
26	66.8	65.1	66.8	75.2	
27	17.3	13.4	17.2	17.4	
	Ac	Gal	Gal	Glc	Gal
1′	21.4	102.4	100.5	104.8	103.0
2′	171.0	73.1	76.3	75.1	72.4
3′		75.5	76.5	78.5	75.4
4′		79.9	70.7	71.6	79.6
5′		76.1	76.6	78.4	75.7
6′		60.6	62.2	62.7	60.6
		Glc (I)	Rha		Glc (I)
1″		105.0	102.0		104.6
2″		81.3	72.4		81.2
3″		86.9	72.7		87.1
4″		70.4	74.3		70.4
5″		77.5	69.3		77.5
6″		62.9	18.6		62.8
		Glc (II)			Glc (II)
1″′		104.7			104.6
2″′		75.3			76.0
3″′		77.7			78.0
4″′		71.0			71.2
5″′		78.6			78.4
6″′		62.4			62.5
		Xyl			Xyl
1″″		104.9			104.8
2″″		75.0			75.0
3″″		78.6			78.6
4″″		70.7			70.8
5″″		67.2			67.2
		Glc (III)			
1″″′		106.3			
2″″′		75.6			
3″″′		78.5			
4″″′		71.7			
5″″′		78.0			
6″″′		62.8			

^(1)^ 125 MHz for **2**, **5**, **6**, **8**, and **9**. 150 MHz for **1**, **3**, **4**, **7**, and **10**.

## Data Availability

Not applicable.

## Supplementary Materials

The following supporting information can be downloaded at: https://www.mdpi.com/article/10.3390/molecules28176248/s1.

## Sample Availability

Isolated compounds are not available from the authors.
