# Supplementary material for: Determination of Structure and Cytotoxicity of Ten Undescribed Steroidal Glycosides from Allium cristophii × A. macleanii ‘Globemaster’
Source: Molecules. 2023 Aug 25;28(17):6248. doi: 10.3390/molecules28176248 (PMC10488941; doi:10.3390/molecules28176248)
Supplement: Supplementary file 1 [file molecules-28-06248-s001.zip › molecules-2559925-supplementary.pdf]

Supplementary material

Determination of structure and cytotoxicity of ten undescribed steroidal glycosides from *Allium cristophii*×*A. macleanii* 'Globemaster'

Tamami Shimazaki, Tomoki Iguchi*, Yuna Takahashi, Kie Yamamoto, Naoki Takahashi, and Yoshihiro Mimaki

Table of contents

- Figure S1. HRESITOFMS data of **1**
- Figure S2. IR spectrum of **1**
- Figure S3. ^{1}H NMR spectrum of **1**
- Figure S4. ^{13}C NMR spectrum of **1**
- Figure S5. ^{1}H-^{1}H COSY spectrum of **1**
- Figure S6. HSQC spectrum of **1**
- Figure S7. HMBC spectrum of **1**
- Figure S8. HRESITOFMS data of **2**
- Figure S9. IR spectrum of **2**
- Figure S10. UV spectrum of **2**
- Figure S11. ^{1}H NMR spectrum of **2**
- Figure S12. ^{13}C NMR spectrum of **2**
- Figure S13. ^{1}H-^{1}H COSY spectrum of **2**
- Figure S14. HSQC spectrum of **2**
- Figure S15. HMBC spectrum of **2**
- Figure S16. HRESITOFMS data of **3**
- Figure S17. IR spectrum of **3**
- Figure S18. ^{1}H NMR spectrum of **3**
- Figure S19. ^{13}C NMR spectrum of **3**

- Figure S20. ^{1}H-^{1}H COSY spectrum of **3**
- Figure S21. HSQC spectrum of **3**
- Figure S22. HMBC spectrum of **3**
- Figure S23. NOESY spectrum of **3**
- Figure S24. HRESITOFMS data of **4**
- Figure S25. IR spectrum of **4**
- Figure S26. ^{1}H NMR spectrum of **4**
- Figure S27. ^{13}C NMR spectrum of **4**
- Figure S28. ^{1}H-^{1}H COSY spectrum of **4**
- Figure S29. HSQC spectrum of **4**
- Figure S30. HMBC spectrum of **4**
- Figure S31. HRESITOFMS data of **5**
- Figure S32. IR spectrum of **5**
- Figure S33. ^{1}H NMR spectrum of **5**
- Figure S34. ^{13}C NMR spectrum of **5**
- Figure S35. ^{1}H-^{1}H COSY spectrum of **5**
- Figure S36. HSQC spectrum of **5**
- Figure S37. HMBC spectrum of **5**
- Figure S38. HRESITOFMS data of **6**

- Figure S39. IR spectrum of **6**
- Figure S40. ^{1}H NMR spectrum of **6**
- Figure S41. ^{13}C NMR spectrum of **6**
- Figure S42. ^{1}H-^{1}H COSY spectrum of **6**
- Figure S43. HSQC spectrum of **6**
- Figure S44. HMBC spectrum of **6**
- Figure S45. HRESITOFMS data of **7**
- Figure S46. IR spectrum of **7**
- Figure S47. ^{1}H NMR spectrum of **7**
- Figure S48. ^{13}C NMR spectrum of **7**
- Figure S49. ^{1}H-^{1}H COSY spectrum of **7**
- Figure S50. HSQC spectrum of **7**
- Figure S51. HMBC spectrum of **7**
- Figure S52. NOESY spectrum of **7**
- Figure S53. HRESITOFMS data of **8**
- Figure S54. IR spectrum of **8**
- Figure S55. ^{1}H NMR spectrum of **8**
- Figure S56. ^{13}C NMR spectrum of **8**
- Figure S57. ^{1}H-^{1}H COSY spectrum of **8**

- Figure S58. HSQC spectrum of **8**
- Figure S59. HMBC spectrum of **8**
- Figure S60. HRESITOFMS data of **9**
- Figure S61. IR spectrum of **9**
- Figure S62. ^{1}H NMR spectrum of **9**
- Figure S63. ^{13}C NMR spectrum of **9**
- Figure S64. ^{1}H-^{1}H COSY spectrum of **9**
- Figure S65. HSQC spectrum of **9**
- Figure S66. HMBC spectrum of **9**
- Figure S67. NOESY spectrum of **9**
- Figure S68. HRESITOFMS data of **10**
- Figure S69. IR spectrum of **10**
- Figure S70. UV spectrum of **10**
- Figure S71. ^{1}H NMR spectrum of **10**
- Figure S72. ^{13}C NMR spectrum of **10**
- Figure S73. ^{1}H-^{1}H COSY spectrum of **10**
- Figure S74. HSQC spectrum of **10**
- Figure S75. HMBC spectrum of **10**

Elemental Composition Report Page 1

Single Mass Analysis
Tolerance = 10.0 mDa / DBE: min = -1.5, max = 300.0
Element prediction: Off
Number of isotope peaks used for i-FIT = 3

Monoisotopic Mass, Even Electron Ions
7 formula(e) evaluated with 1 results within limits (up to 50 closest results for each mass)
Elements Used:
C: 1-300 H: 1-1000 O: 17-17 Na: 1-1
AGM-B2-1-3-2-5
M-162769-1 56 (0.522) AM2 (Ar,22000.0,0.00,0.00); ABS; Cm (25:60) 1: TOF MS ES+ 1.09e+008

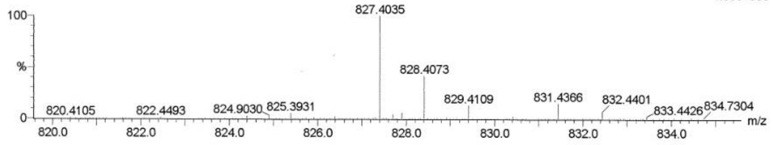

Minimum:				-1.5				
Maximum:		10.0	50.0	300.0				
Mass	Calc. Mass	mDa	PPM	DBE	i-FIT	Norm	Conf(%)	Formula
827.4035	827.4041	-0.6	-0.7	7.5	613.5	n/a	n/a	C39 H64 O17 Na

Figure S1. HRESITOFMS data of **1**

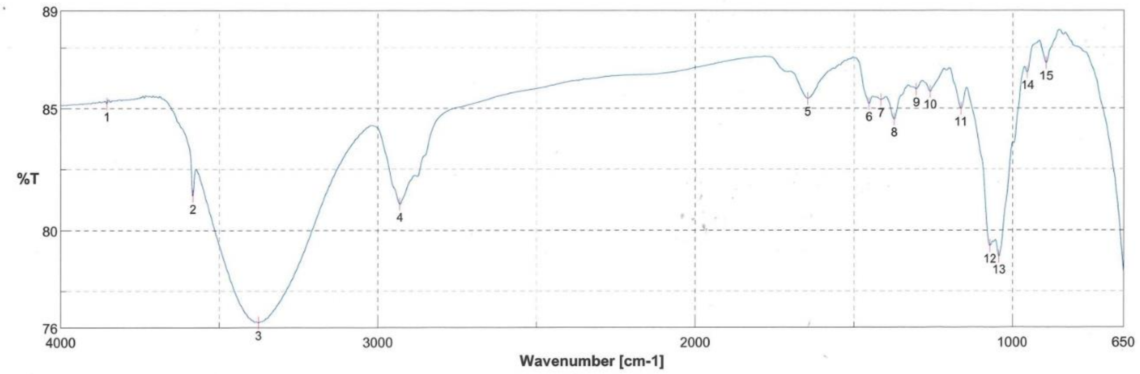

積算回数	40	分解	4 cm-1
ゼロフィリング	ON	アポダイゼーション	Cosine
ゲイン	Auto (1)	スキャンスピード	Auto (2 mm/sec)
測定日時	2021/03/01 14:54	更新日時	2021/03/01 14:55
測定者	YunaTakahashi		
ファイル名	Memory#1		
サンプル名	AGM-B1-4-6		
コメント	film		

No.	cm-1	%T	No.	cm-1	%T	No.	cm-1	%T	No.	cm-1	%T	No.	cm-1	%T
1	3854. 04	85. 1911	2	3583. 09	81. 4131	3	3375. 78	76. 2065	4	2930. 31	81. 0764	5	1645. 95	85. 4037
6	1452. 14	85. 1867	7	1415. 49	85. 3467	8	1374. 03	84. 5555	9	1303. 64	85. 7898	10	1259. 29	85. 6922
11	1162. 87	84. 9812	12	1071. 26	79. 3699	13	1041. 37	78. 9249	14	951. 698	86. 4858	15	894. 809	86. 8652

Figure S2. IR spectrum of **1**

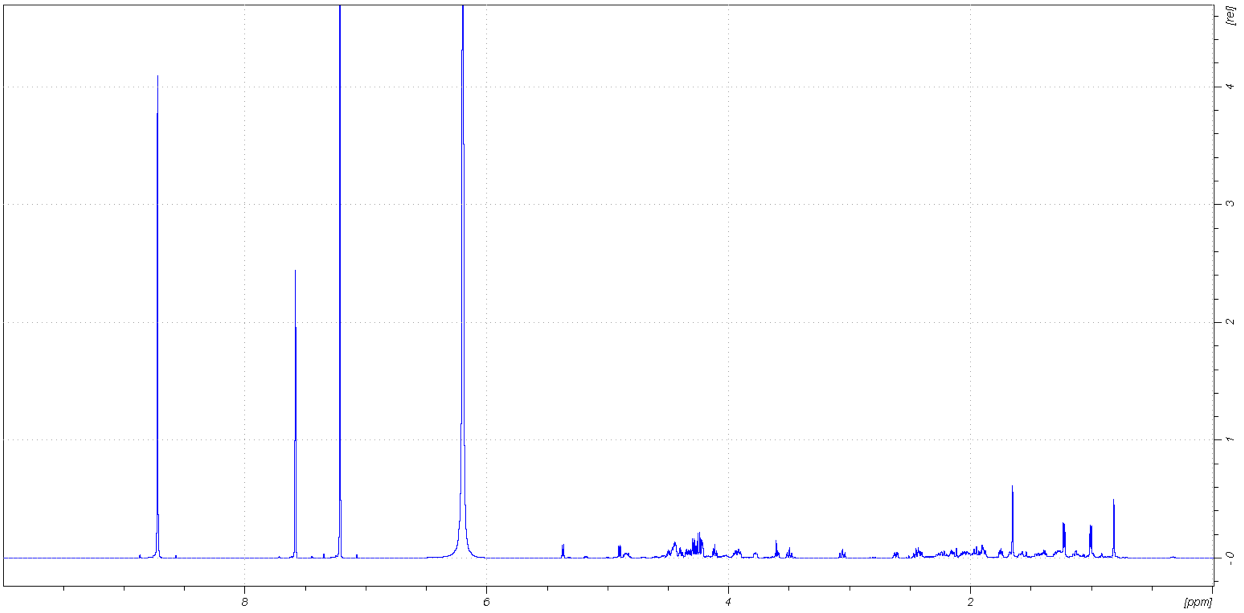

Figure S3. ^{1}H NMR spectrum of **1**

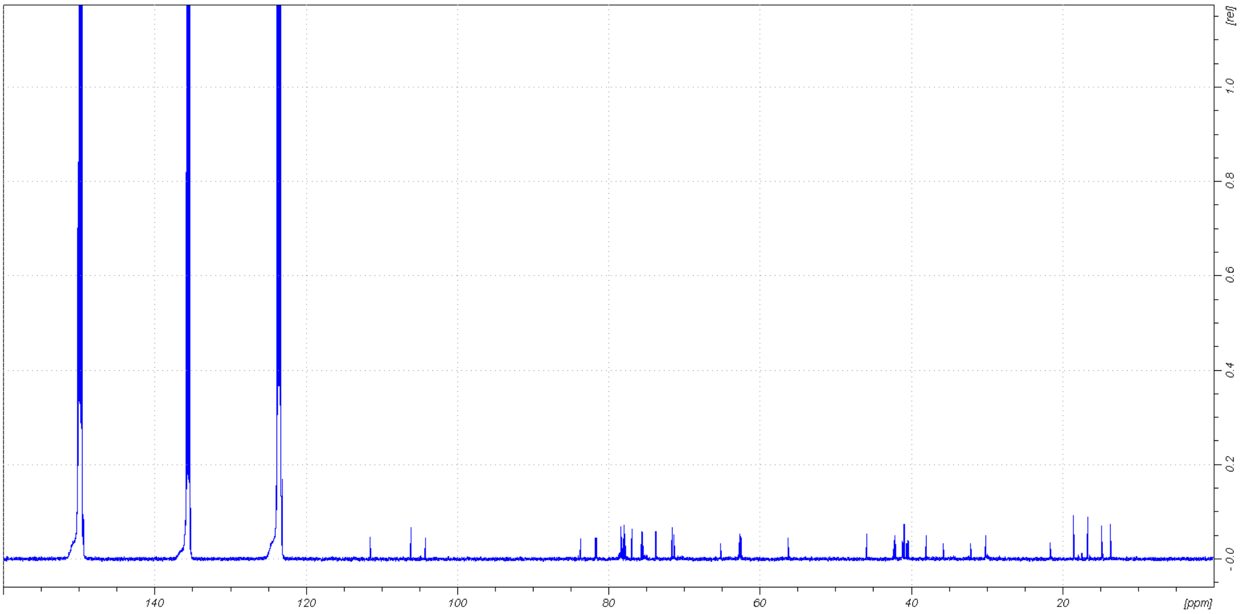

Figure S4. ^{13}C NMR spectrum of **1**

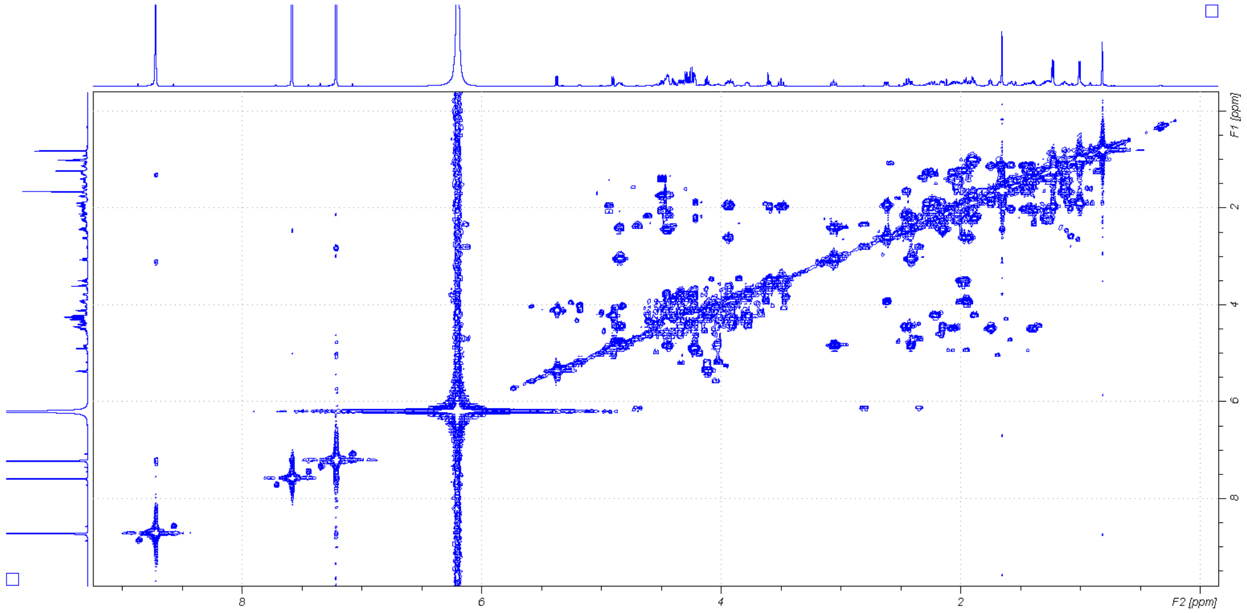

Figure S5. ^{1}H-^{1}H COSY spectrum of **1**

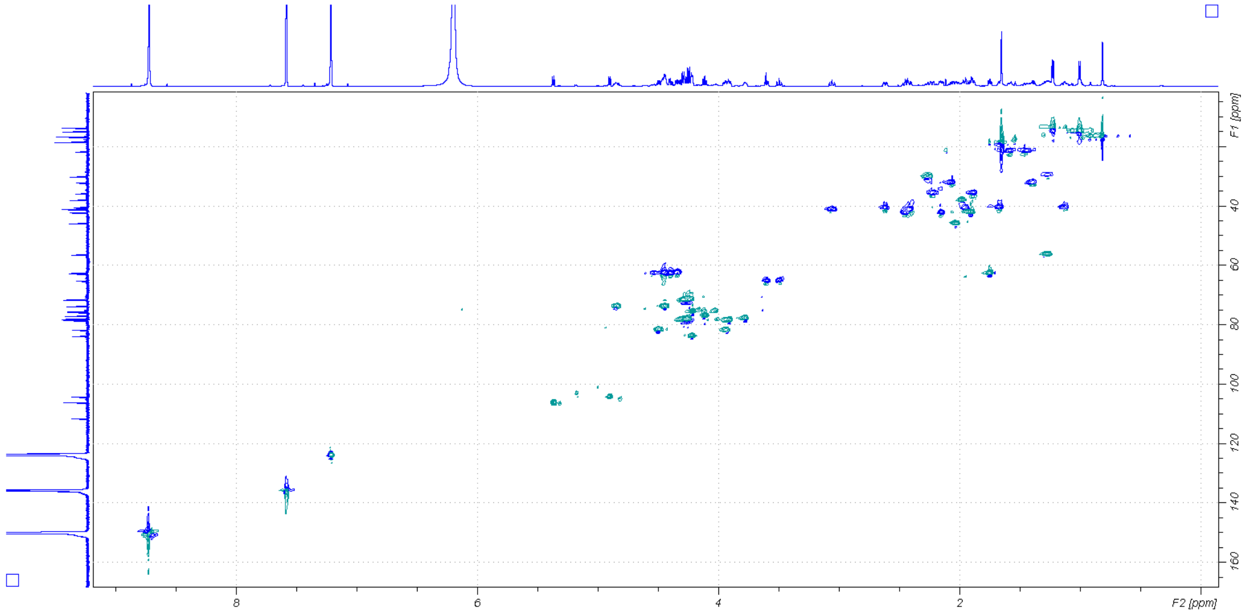

Figure S6. HSQC spectrum of **1**

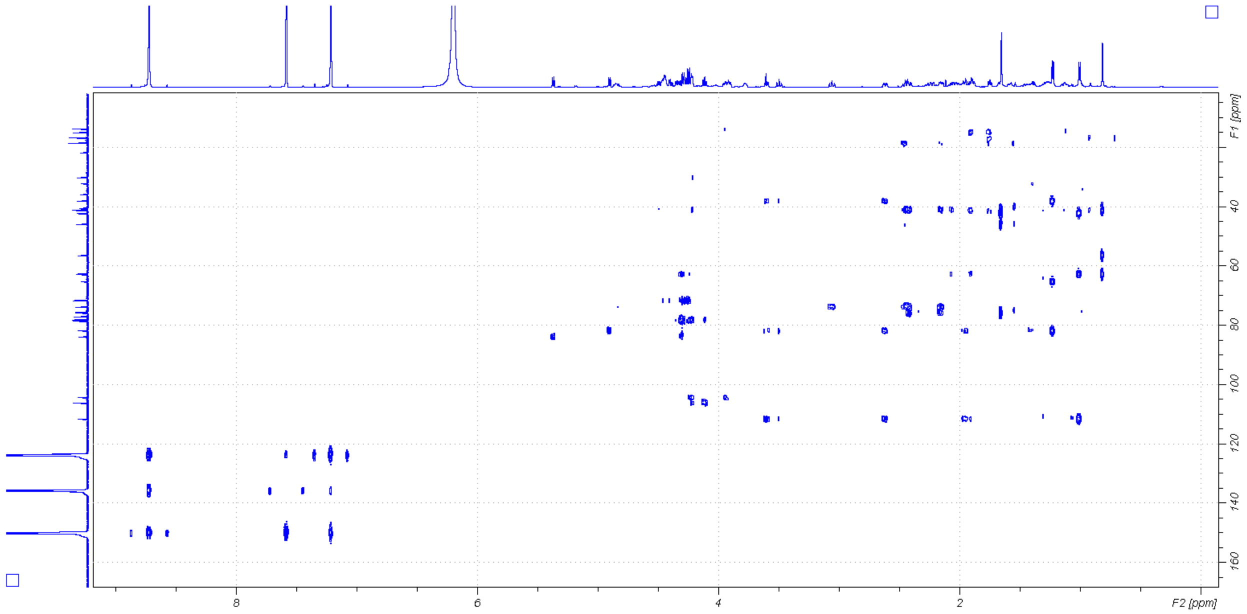

Figure S7. HMBC spectrum of **1**

Elemental Composition Report Page 1

Single Mass Analysis
Tolerance = 10.0 mDa / DBE: min = -1.5, max = 300.0
Element prediction: Off
Number of isotope peaks used for i-FIT = 3

Monoisotopic Mass, Even Electron Ions
9 formula(e) evaluated with 1 results within limits (up to 50 closest results for each mass)
Elements Used:
C: 0-300 H: 0-1000 O: 23-23 Na: 1-1
AGM-B1-4-9-4-3
M-17339 188 (1.521) AM2 (Ar,22000.0,0.00,0.00); ABS; Cm (187:224)
1: TOF MS ES+
7.56e+007

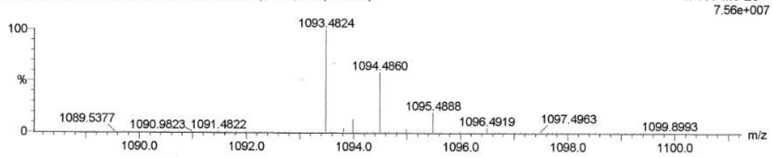

Minimum:				-1.5				
Maximum:		10.0	50.0	300.0				
Mass	Calc. Mass	mDa	PPM	DBE	i-FIT	Norm	Conf(%)	Formula
1093.4824	1093.4832	-0.8	-0.7	13.5	359.4	n/a	n/a	C52 H78 O23 Na

Figure S8. HRESITOFMS data of **2**

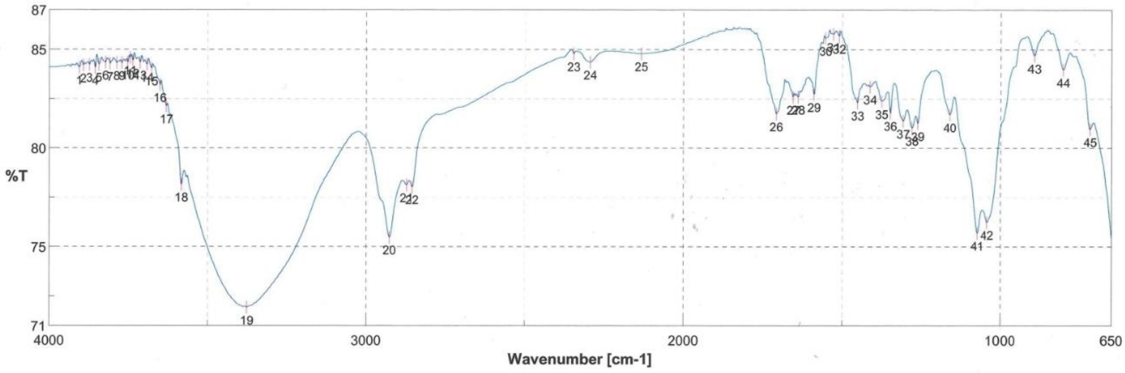

積算回数	40	分解	4 cm-1
ゼロフィリング	ON	アポダイゼーション	Cosine
ゲイン	Auto (1)	スキャンスピード	Auto (2 mm/sec)
測定日時	2021/06/08 14:13	更新日時	2021/06/08 14:16
測定者	Yuna Takahashi		
ファイル名	Memory#1		
サンプル名	AGM-19		
コメント	film		

No.	cm-1	%T	No.	cm-1	%T	No.	cm-1	%T	No.	cm-1	%T	No.	cm-1	%T
1	3906. 11	84. 0752	2	3892. 61	84. 1783	3	3873. 33	84. 2187	4	3855. 01	84. 0638	5	3843. 43	84. 2261
6	3823. 19	84. 324	7	3808. 72	84. 2623	8	3787. 51	84. 3082	9	3771. 12	84. 3028	10	3752. 8	84. 3306
11	3745. 08	84. 5702	12	3736. 4	84. 4233	13	3713. 26	84. 3251	14	3692. 05	84. 2184	15	3677. 59	84. 0356
16	3650. 59	83. 1916	17	3630. 34	82. 1216	18	3583. 09	78. 1497	19	3375. 78	71. 9175	20	2926. 45	75. 473
21	2872. 45	78. 1199	22	2855. 1	78. 0241	23	2345. 98	84. 7695	24	2294. 88	84. 3556	25	2133. 85	84. 7779
26	1708. 62	81. 7306	27	1654. 62	82. 5812	28	1639. 2	82. 5883	29	1590. 02	82. 715	30	1552. 42	85. 5685
31	1527. 35	85. 7676	32	1509. 99	85. 6808	33	1451. 17	82. 2952	34	1412. 6	83. 1156	35	1375. 96	82. 3695
36	1348	81. 7678	37	1307. 5	81. 3783	38	1279. 54	81. 0195	39	1261. 22	81. 2459	40	1159. 97	81. 7001
41	1073. 19	75. 6927	42	1041. 37	76. 24	43	893. 844	84. 6837	44	803. 206	83. 9745	45	716. 425	80. 9552

Figure S9. IR spectrum of **2**

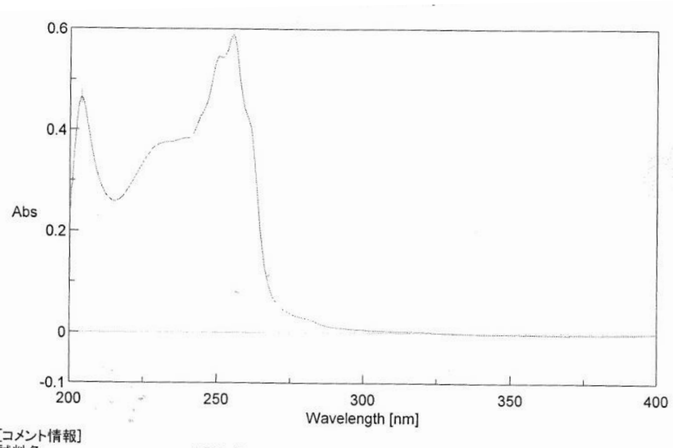

[コメント情報]

試料名	AGM-19
コメント	0.5mg/10mL
測定者	Yuna Takahashi
所属	
会社	東京薬科大学　漢方ｴ

[データ情報]

作成日時	2021/06/07 11:14
データタイプ	等間隔データ
横軸	Wavelength [nm]
縦軸	Abs
スタート	400 nm
エンド	200 nm
データ間隔	0.1 nm
データ数	2001

[測定情報]

装置名	V-630
機種名	V-630
シリアル番号	A017661148
付属品名	USE-753
付属品シリアルNo.	A017661148
セル長	10 mm
測光モード	Abs
測定範囲	400 - 200 nm
データ取込間隔	0.1 nm
UV/Vis バンド幅	1.5 nm
レスポンス	Fast
走査速度	40 nm/min
光源切換	340 nm
光源	D2/WI
フィルタ切換	ステップ
補正	ベースライン

[ピーク検出結果]

No.	位置	強度	No.	位置	強度	No.	位置	強度
1	255.2	0.58852	2	204	0.466314			

Figure S10. UV spectrum of **2**

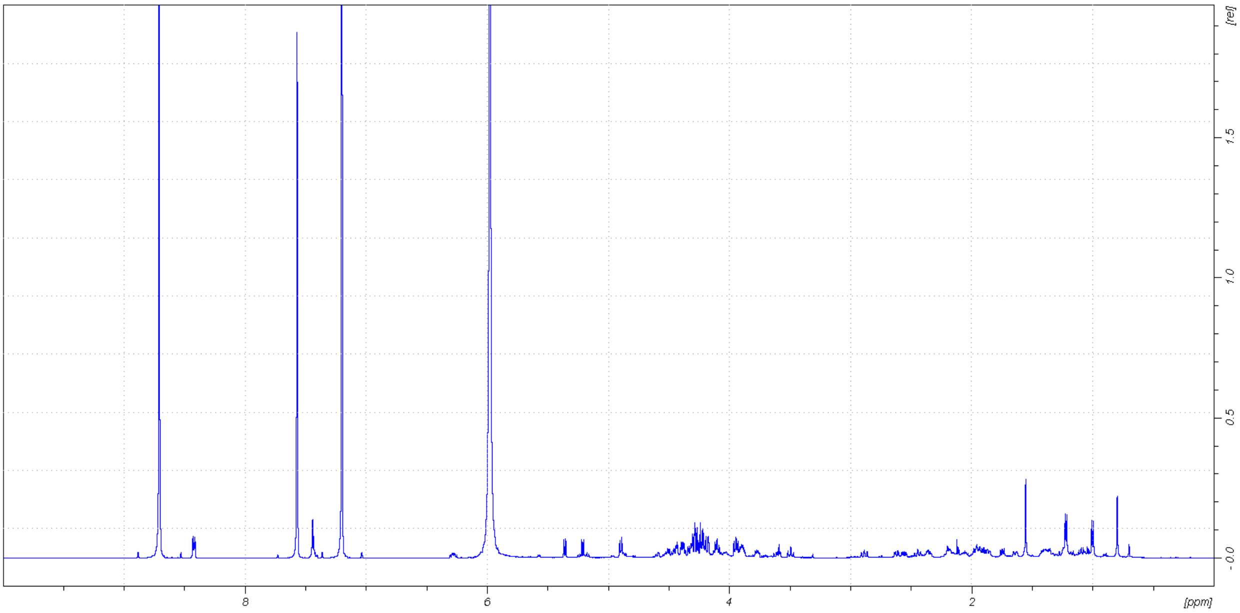

Figure S11. ^{1}H NMR spectrum of **2**

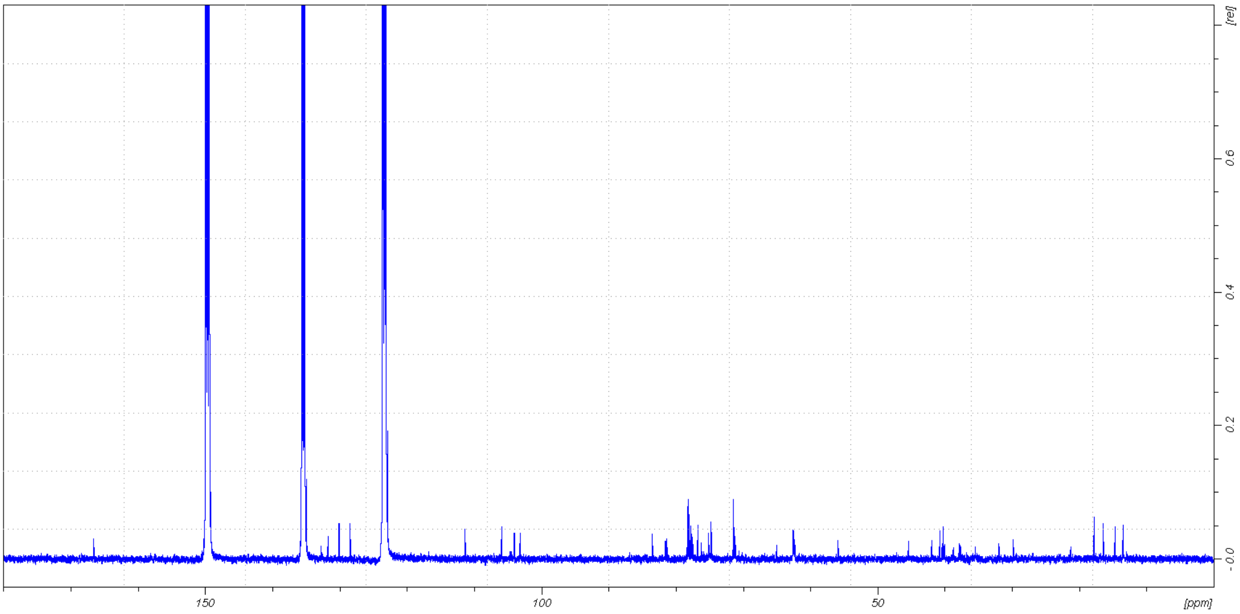

Figure S12. ^{13}C NMR spectrum of **2**

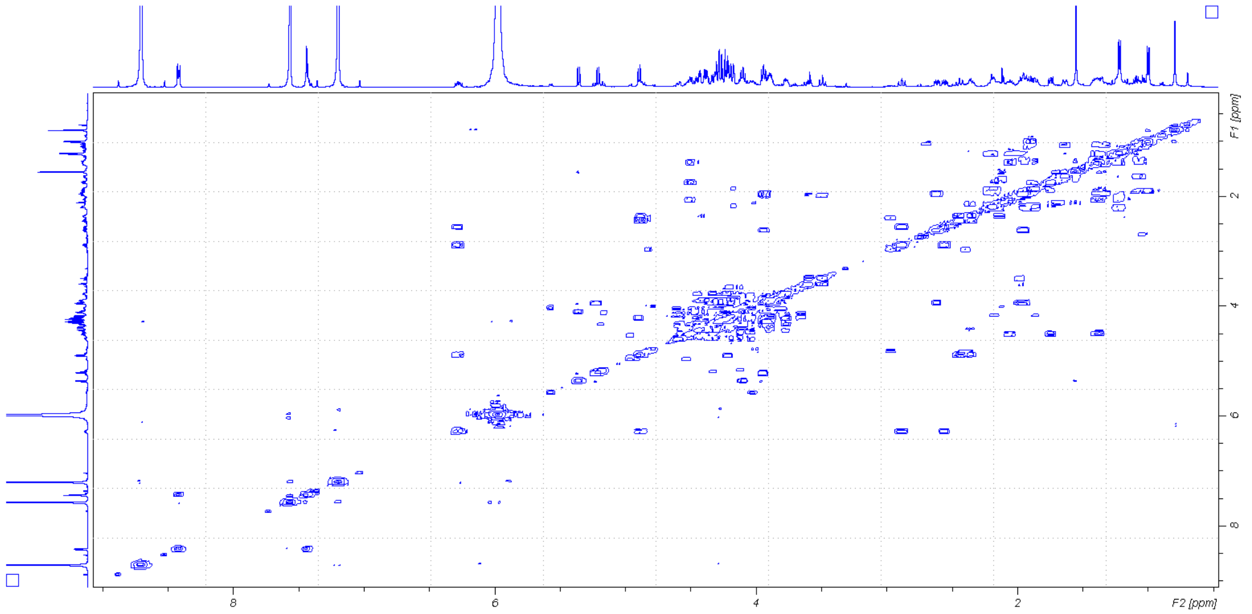

Figure S13. ^{1}H-^{1}H COSY spectrum of **2**

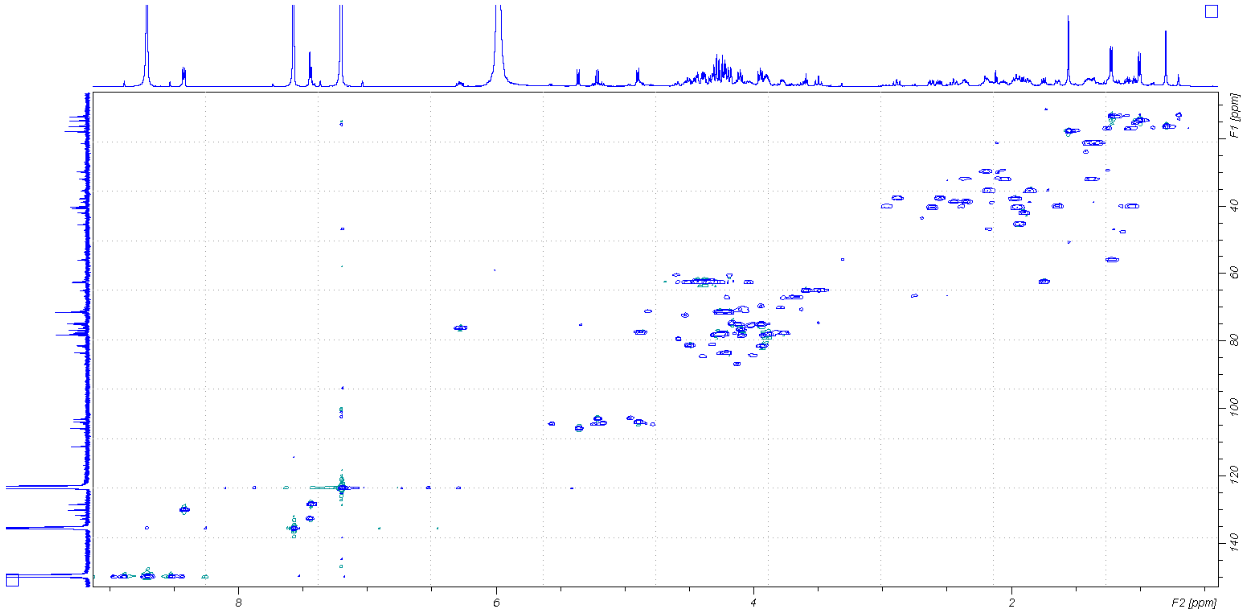

Figure S14. HSQC spectrum of **2**

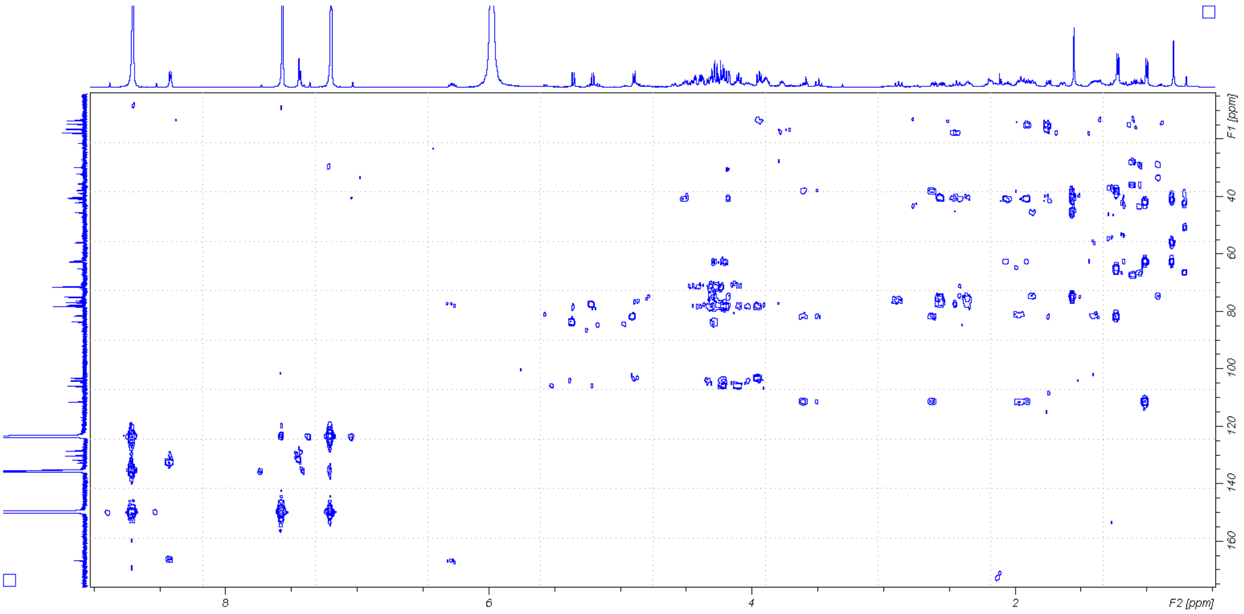

Figure S15. HMBC spectrum of **2**

Elemental Composition Report Page 1

Single Mass Analysis
Tolerance = 10.0 mDa / DBE: min = -1.5, max = 300.0
Element prediction: Off
Number of isotope peaks used for i-FIT = 3

Monoisotopic Mass, Even Electron Ions
10 formula(e) evaluated with 1 results within limits (up to 50 closest results for each mass)
Elements Used:
C: 0-300 H: 0-3000 O: 25-25 Na: 1-1
AGM-C5-4-1-2-4-5
M-19024 740 (7.427) AM2 (Ar,22000.0,0.00,0.00); ABS; Cm (720:750) 1: TOF MS ES+ 4.53e+007

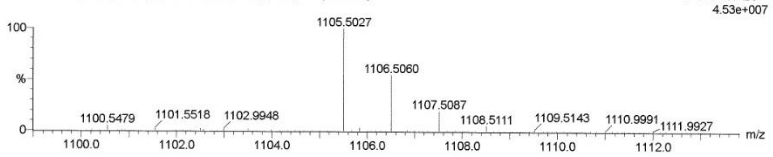

	Calc. Mass	mDa	PPM	DBE	i-FIT	Norm	Conf(%)	Formula
Minimum:				-1.5				
Maximum:		10.0	5.0	300.0				
Mass	Calc. Mass	mDa	PPM	DBE	i-FIT	Norm	Conf(%)	Formula
1105.5027	1105.5043	-1.6	-1.4	9.5	352.1	n/a	n/a	C50 H82 O25 Na

Figure S16. HRESITOFMS data of **3**

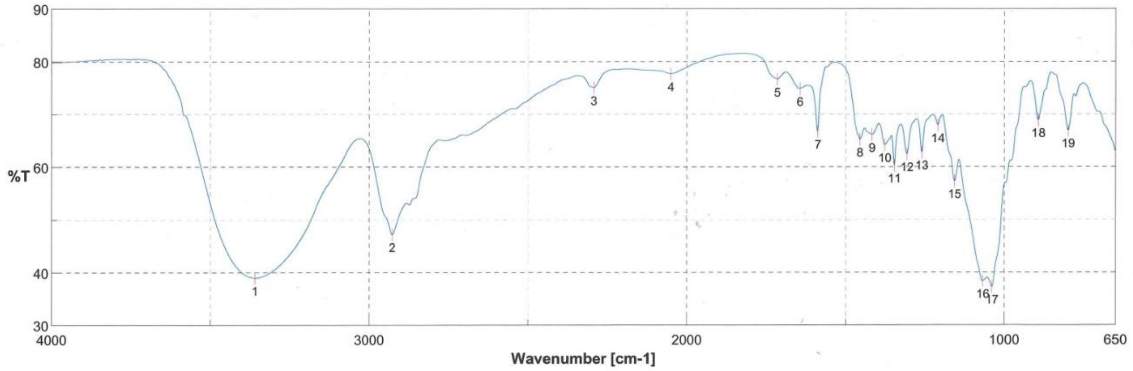

積算回数	40	分解	4 cm-1
ゼロフィリング	ON	アポダイゼーション	Cosine
ゲイン	Auto (1)	スキャンスピード	Auto (2 mm/sec)
測定日時	2011/12/30 4:53	更新日時	2011/12/30 4:54
測定者	yamamoto kie		
ファイル名	Memory#1		
サンプル名	AGM-C5-4-1-2-4-5		
コメント	film		

No.	cm-1	%T	No.	cm-1	%T	No.	cm-1	%T	No.	cm-1	%T	No.	cm-1	%T
1	3358.43	38.959	2	2926.45	47.2008	3	2292.95	75.0554	4	2050.92	77.6512	5	1715.37	76.6173
6	1644.98	74.841	7	1589.06	66.7347	8	1455.03	65.2056	9	1417.42	66.1034	10	1377.89	64.1928
11	1347.03	60.2929	12	1307.5	62.44	13	1260.25	62.7589	14	1210.11	67.926	15	1157.08	57.3433
16	1068.37	38.3341	17	1038.48	37.2882	18	893.844	68.8971	19	800.314	66.9354			

Figure S17. IR spectrum of **3**

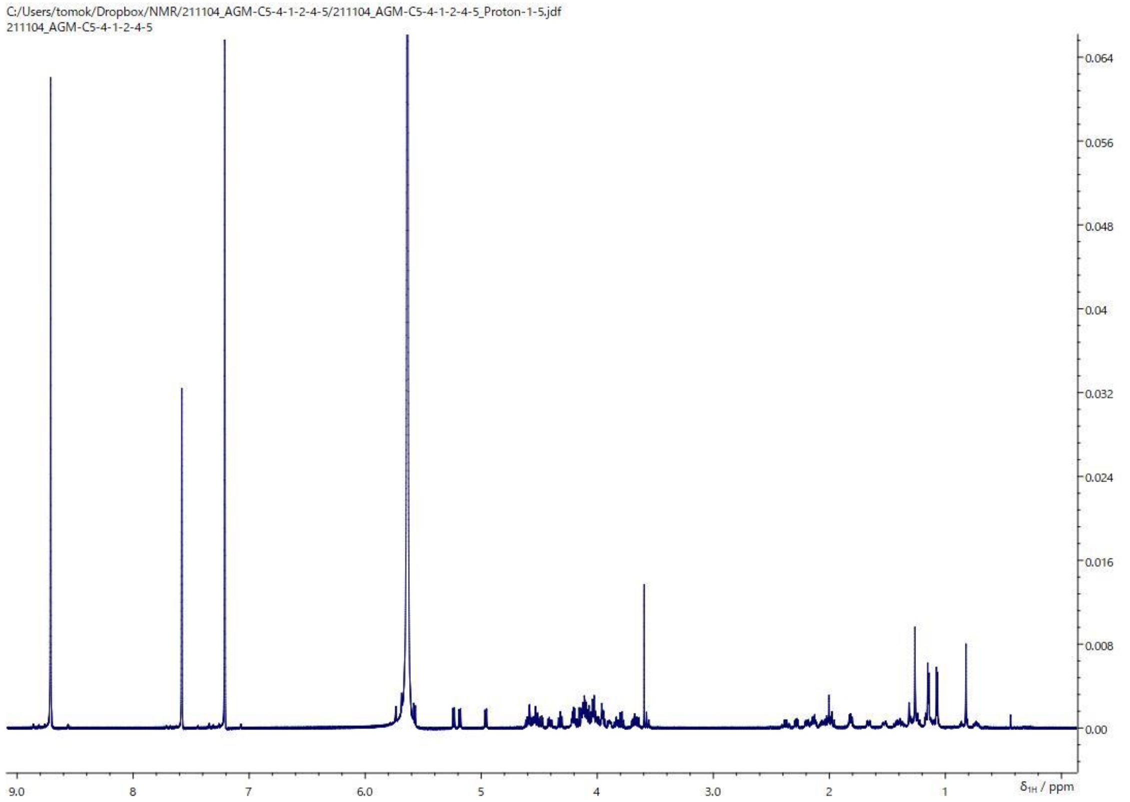

Figure S18. ^{1}H NMR spectrum of **3**

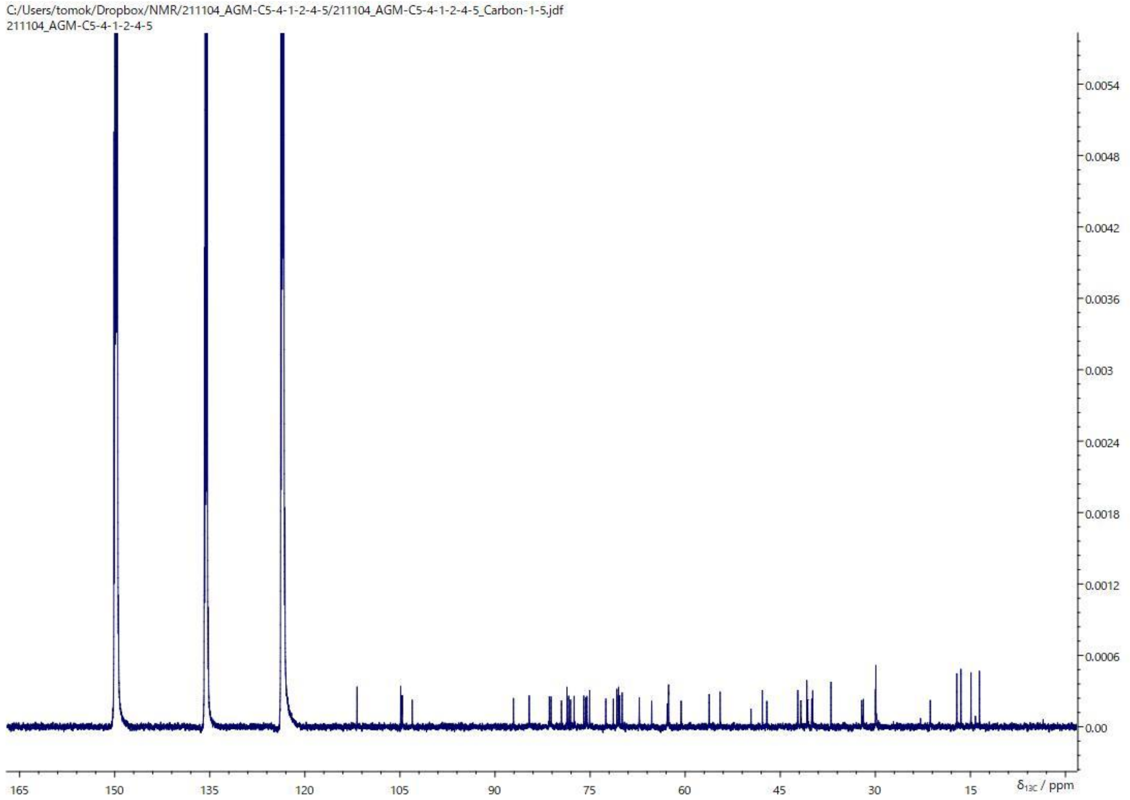

Figure S19. ^{13}C NMR spectrum of **3**

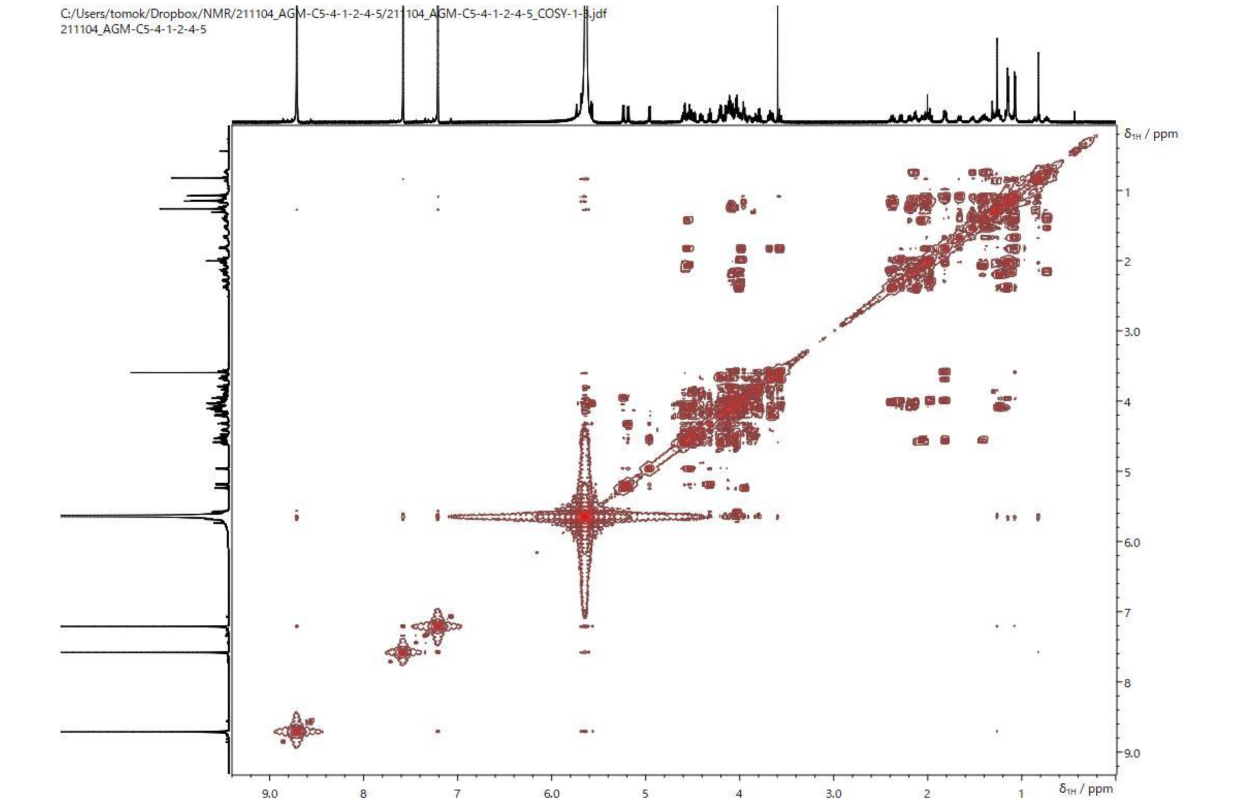

Figure S20. ^{1}H-^{1}H COSY spectrum of **3**

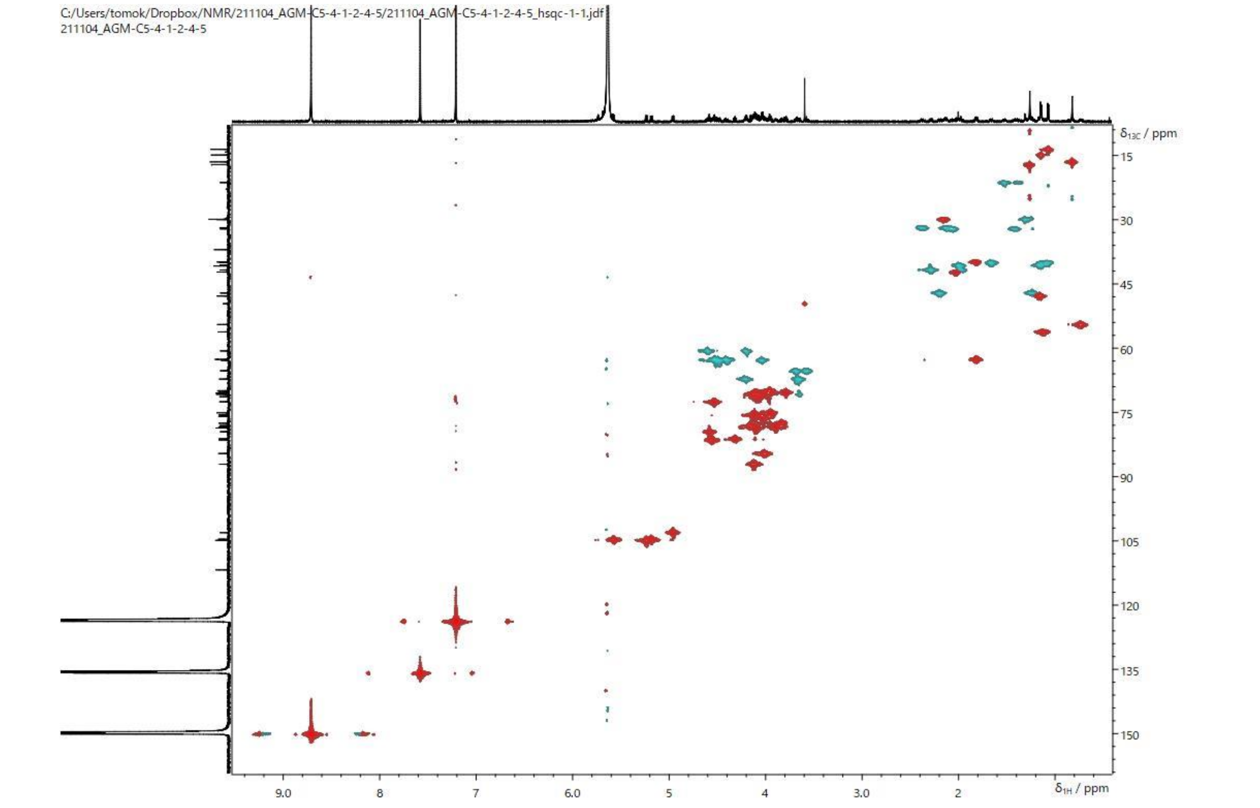

Figure S21. HSQC spectrum of **3**

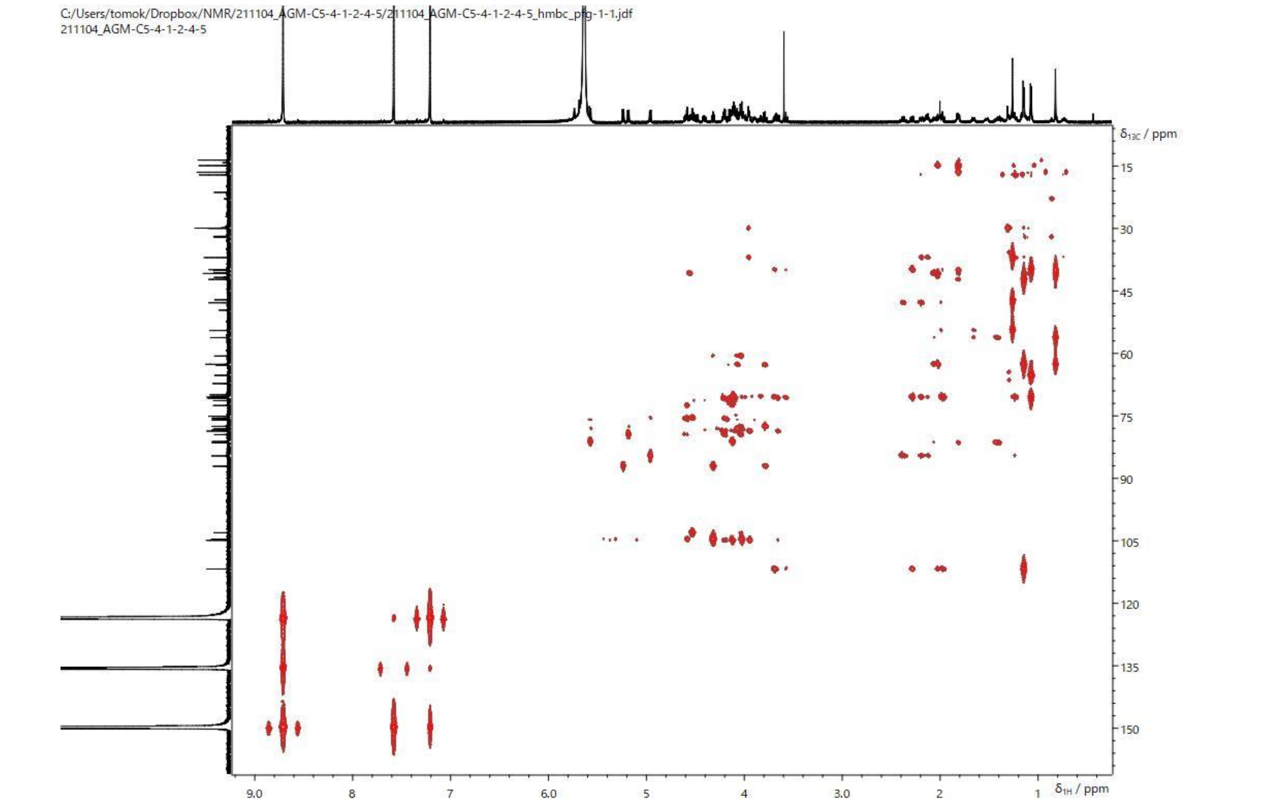

Figure S22. HMBC spectrum of **3**

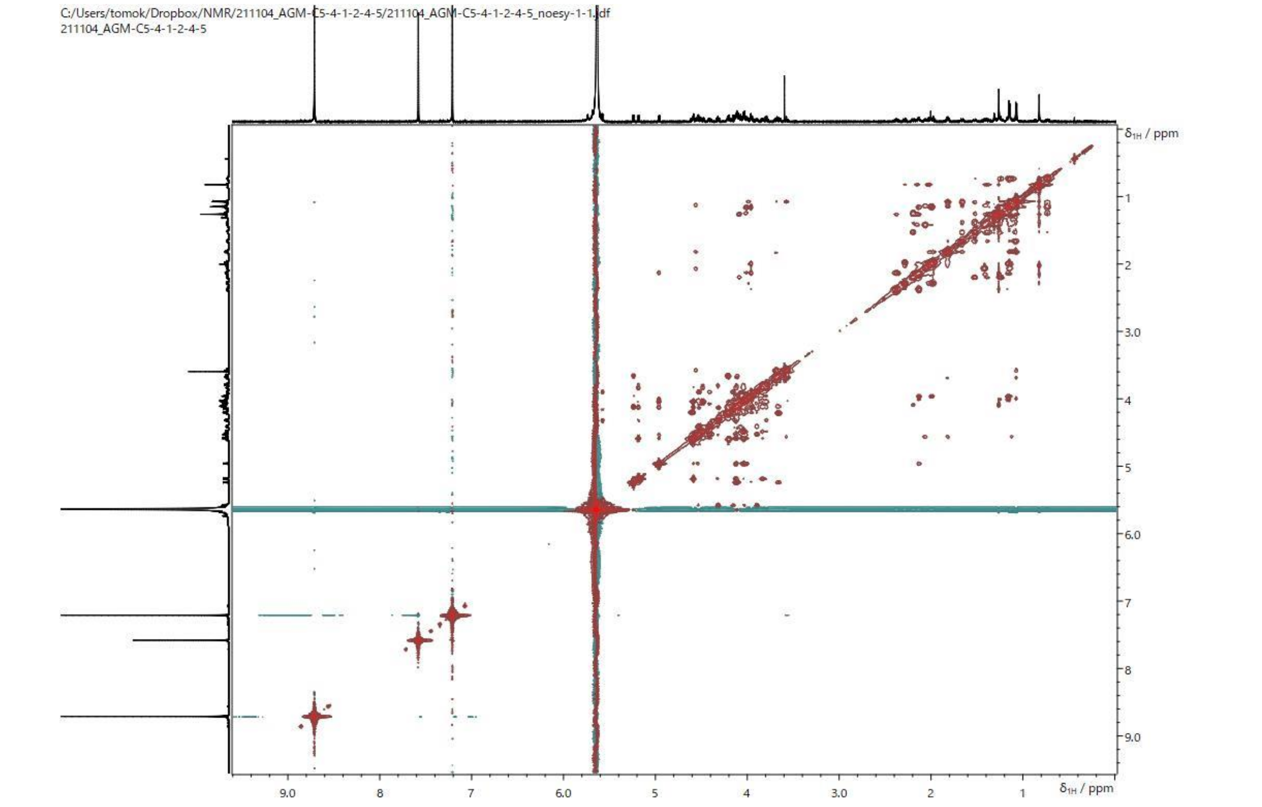

Figure S23. NOESY spectrum of **3**

Elemental Composition Report Page 1

Single Mass Analysis
Tolerance = 10.0 mDa / DBE: min = -1.5, max = 300.0
Element prediction: Off
Number of isotope peaks used for i-FIT = 3

Monoisotopic Mass, Even Electron Ions
10 formula(e) evaluated with 1 results within limits (up to 50 closest results for each mass)
Elements Used:
C: 1-600 H: 1-1000 O: 30-30 Na: 1-1
AGM-B2-2-8-4
M-16607 78 (0.714) AM2 (Ar,22000.0,0.00,0.00); ABS; Cm (53:87) 1: TOF MS ES+ 1.07e+008

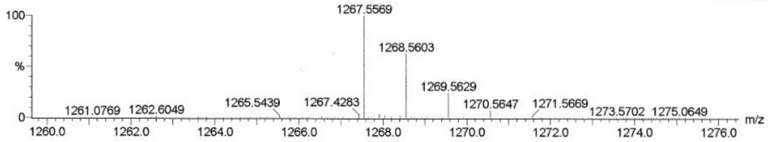

Minimum:				-1.5				
Maximum:		10.0	50.0	300.0				
Mass	Calc. Mass	mDa	PPM	DBE	i-FIT	Norm	Conf(%)	Formula
1267.5569	1267.5571	-0.2	-0.2	10.5	404.3	n/a	n/a	C56 H92 O30 Na

Figure S24. HRESITOFMS data of **4**

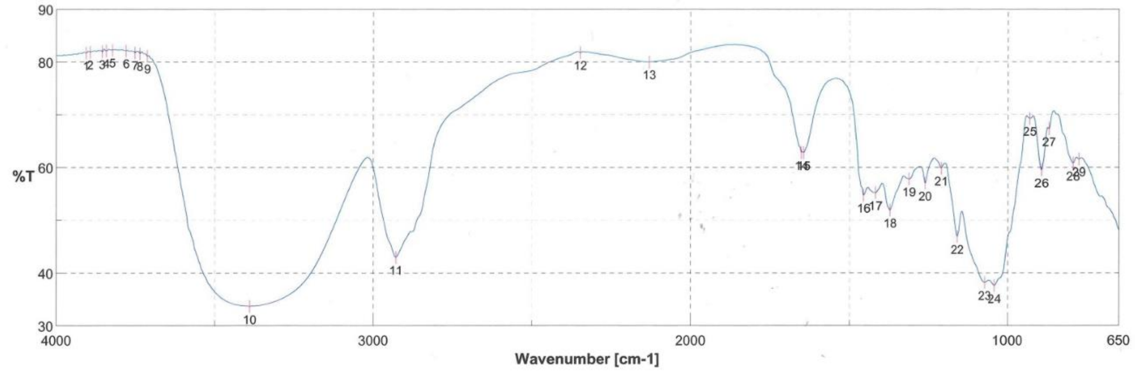

積算回数	40	分解	4 cm-1
ゼロフィリング	ON	アポダイゼーション	Cosine
ゲイン	Auto (1)	スキャンスピード	Auto (2 mm/sec)
測定日時	2021/06/08 13:47	更新日時	2021/06/08 13:48
測定者	Yuna Takahashi		
ファイル名	Memory#1		
サンプル名	AGM-6		
コメント	film		

No. cm-1	%T	No. cm-1	%T	No. cm-1	%T	No. cm-1	%T	No. cm-1	%T
1 3906. 11	81. 7315	2 3892. 61	81. 9002	3 3855. 01	81. 965	4 3842. 47	82. 1276	5 3823. 19	82. 2789
6 3779. 8	82. 1585	7 3751. 83	81. 8038	8 3736. 4	81. 6301	9 3712. 3	81. 1334	10 3389. 28	33. 6118
11 2928. 38	42. 9421	12 2347. 91	81. 786	13 2130. 96	80. 0098	14 1650. 77	62. 803	15 1644. 02	62. 772
16 1454. 06	54. 7083	17 1417. 42	55. 1832	18 1372. 1	51. 8958	19 1311. 36	57. 7979	20 1260. 25	56. 9772
21 1210. 11	59. 8498	22 1159. 01	46. 8951	23 1072. 23	38. 1567	24 1040. 41	37. 5431	25 928. 557	69. 2363
26 893. 844	59. 4918	27 871. 667	67. 2727	28 794. 528	60. 7923	29 776. 208	61. 5535		

Figure S25. IR spectrum of **4**

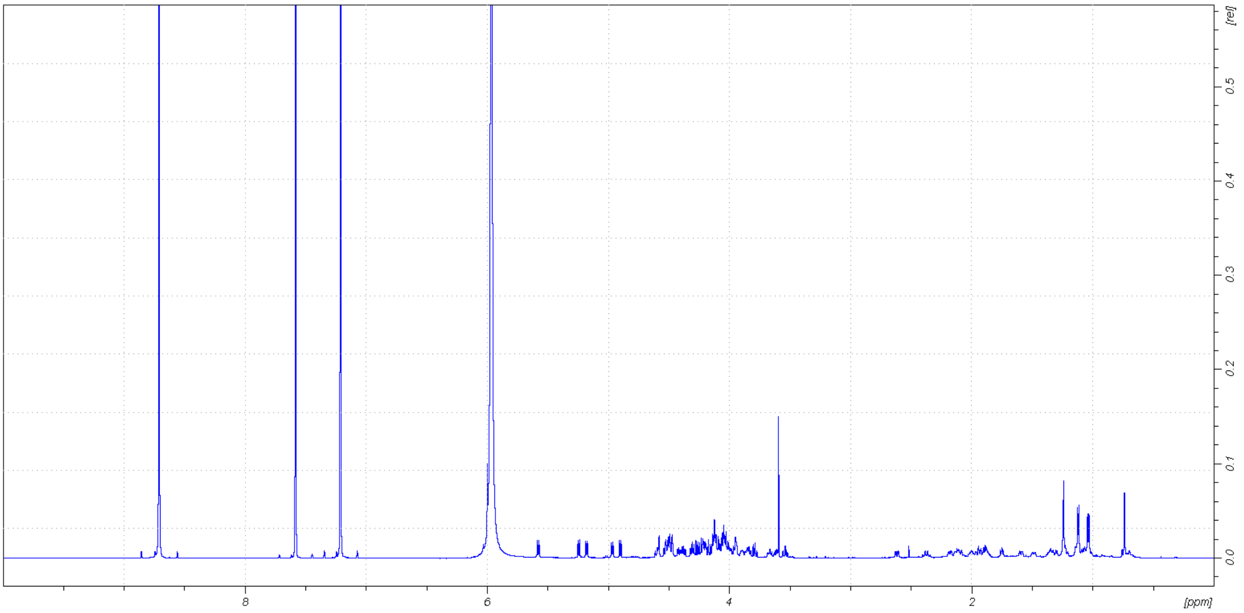

Figure S26. ^{1}H NMR spectrum of **4**

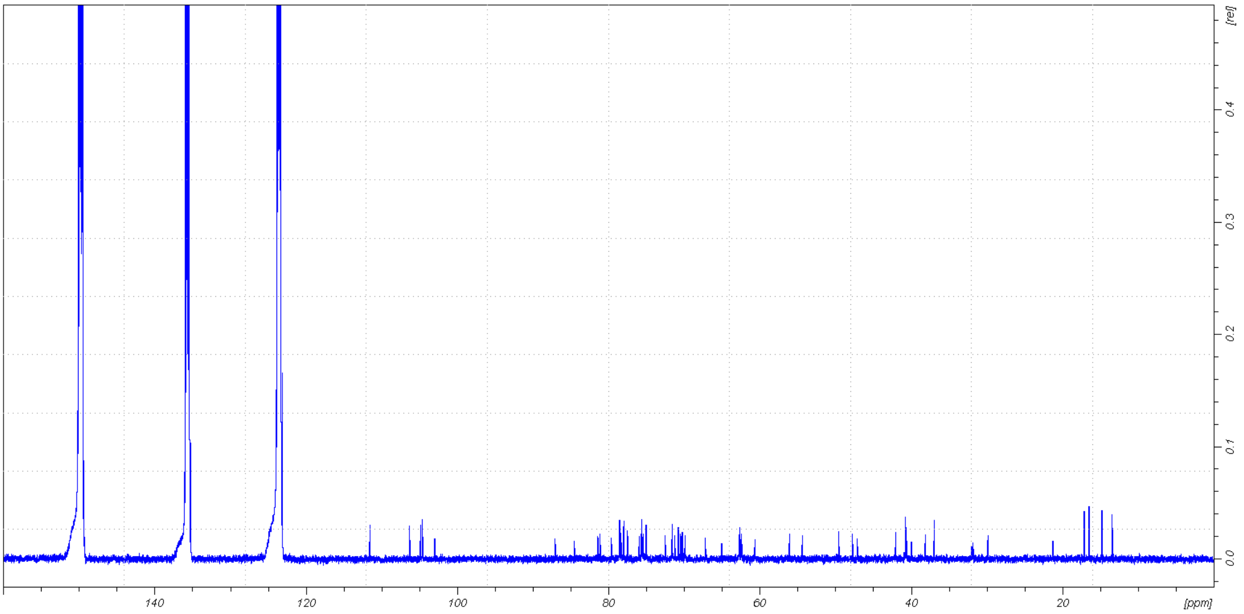

Figure S27. ^{13}C NMR spectrum of **4**

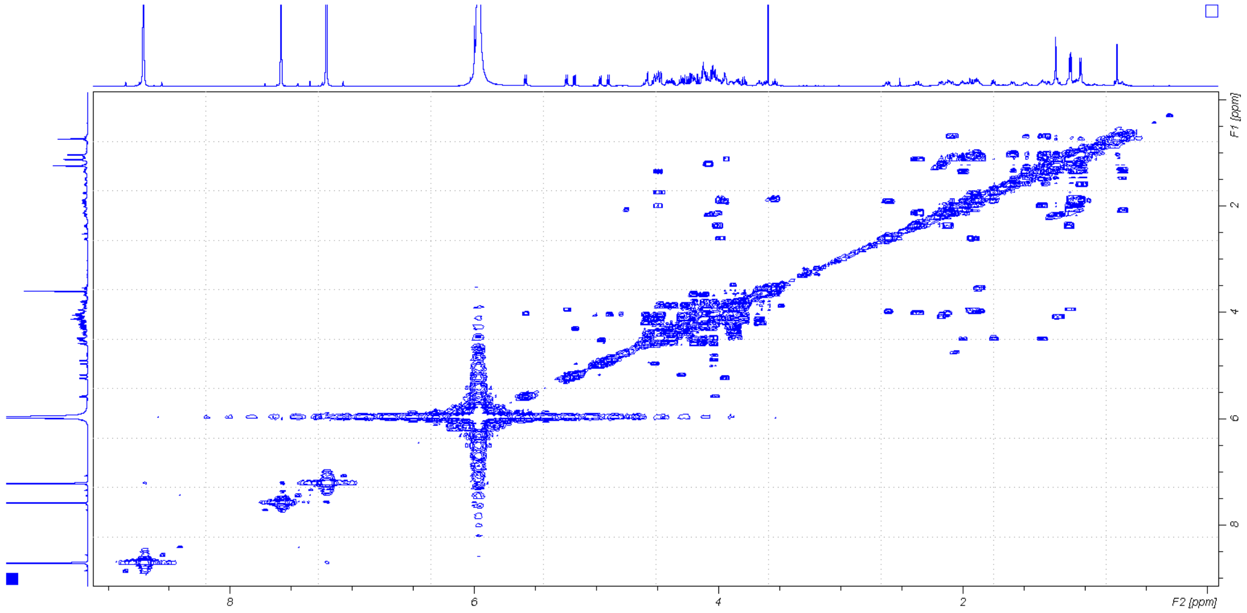

Figure S28. ¹H-¹H COSY spectrum of **4**

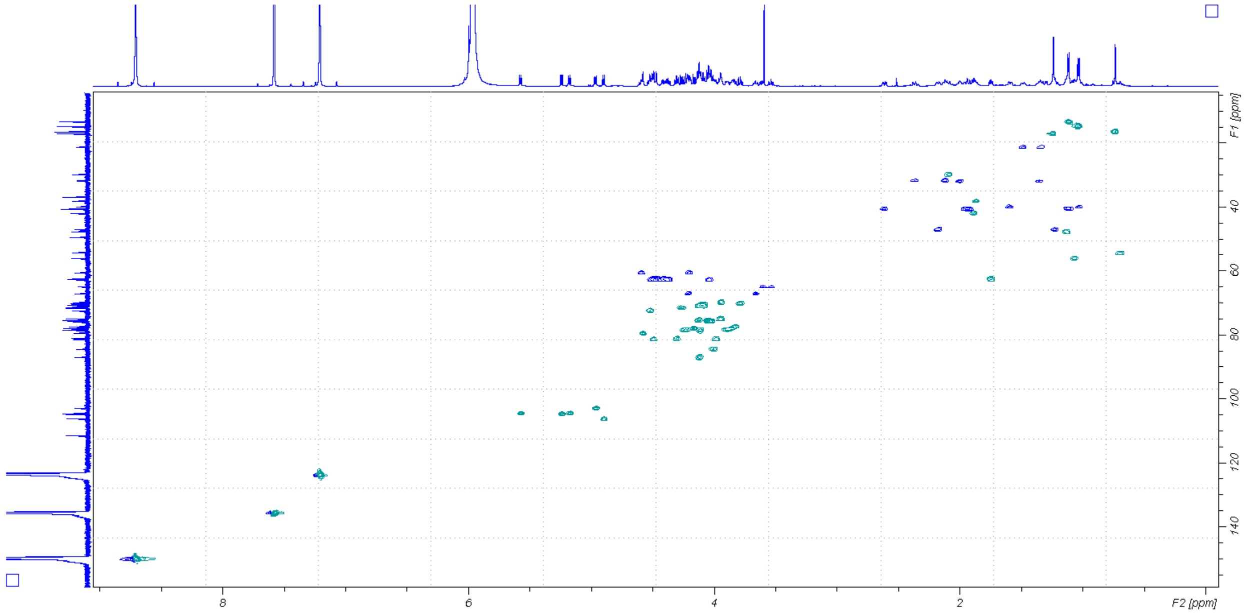

Figure S29. HSQC spectrum of **4**

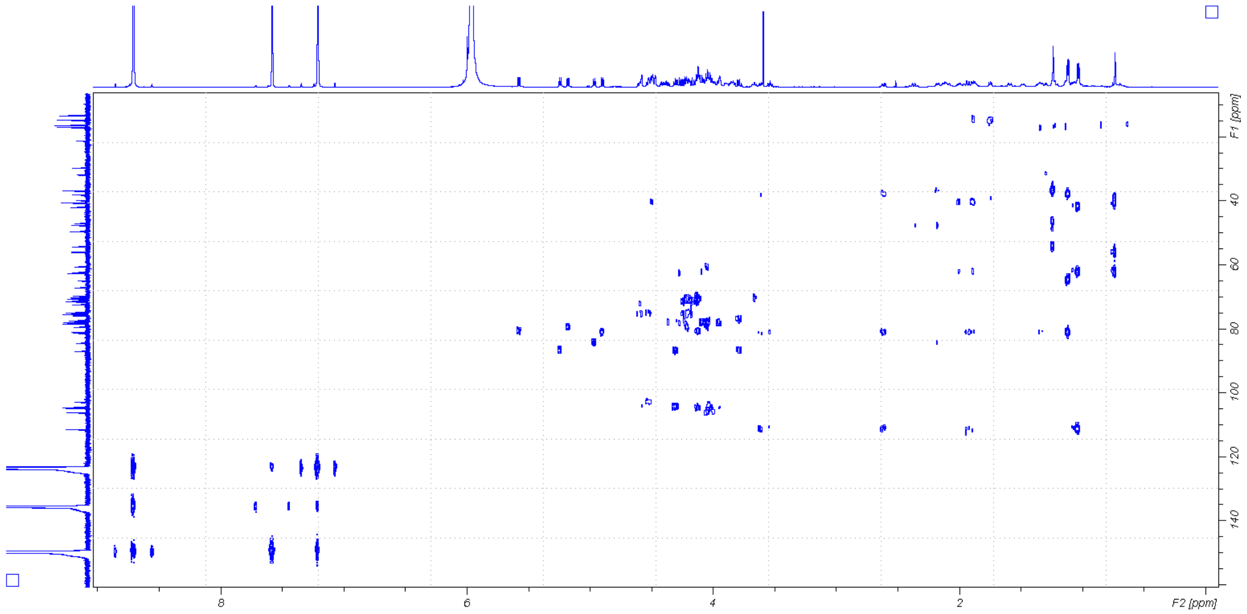

Figure S30. HMBC spectrum of **4**

Elemental Composition Report **Page 1**

Single Mass Analysis
Tolerance = 10.0 mDa / DBE: min = -1.5, max = 300.0
Element prediction: Off
Number of isotope peaks used for i-FIT = 3

Monoisotopic Mass, Even Electron Ions
11 formula(e) evaluated with 1 results within limits (up to 50 closest results for each mass)
Elements Used:
C: 1-300 H: 1-1000 O: 35-35 Na: 1-1
AGM-B1-5-1-9
M-16976 166 (1.347) AM2 (Ar,22000.0,0.00,0.00); ABS; Sb (10,10.00); Sm (SG, 3x3.00); Cm (160:198) 1: TOF MS ES+ 5.08e+007

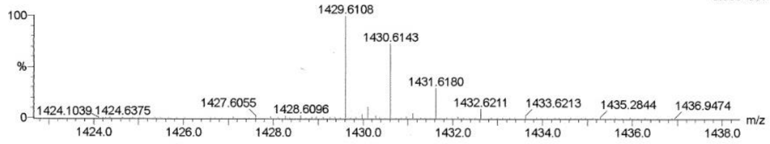

Minimum:				-1.5				
Maximum:		10.0	50.0	300.0				
Mass	Calc. Mass	mDa	PPM	DBE	i-FIT	Norm	Conf(%)	Formula
1429.6108	1429.6099	0.9	0.6	11.5	453.5	n/a	n/a	C62 H102 O35 Na

Figure S31. HRESITOFMS data of **5**

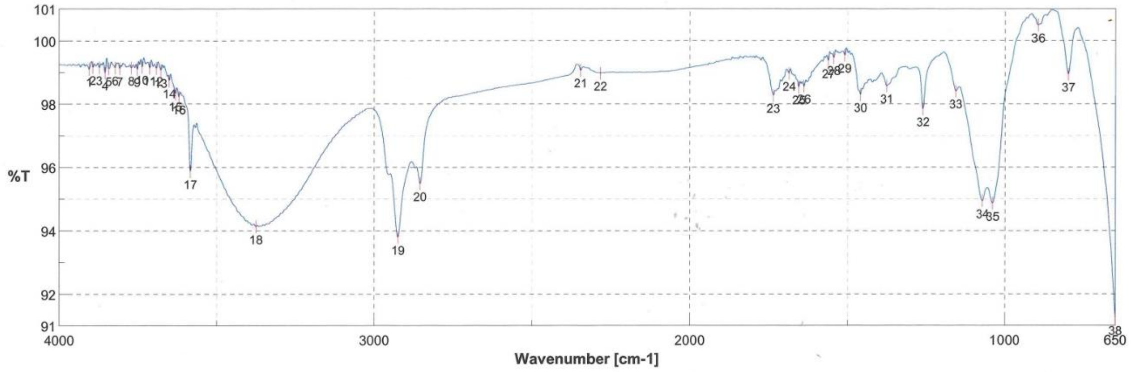

積算回数	40	分解	4 cm-1
ゼロフィリング	ON	アポダイゼーション	Cosine
ゲイン	Auto (1)	スキャンスピード	Auto (2 mm/sec)
測定日時	2021/06/08 13:56	更新日時	2021/06/08 13:58
測定者	Yuna Takahashi		
ファイル名	Memory#3		
サンプル名	AGM-9		
コメント	film		

No. cm-1	%T	No. cm-1	%T	No. cm-1	%T	No. cm-1	%T	No. cm-1	%T
1 3904. 18	99. 1029	2 3892. 61	99. 1637	3 3872. 36	99. 1563	4 3854. 04	98. 983	5 3842. 47	99. 119
6 3822. 22	99. 1454	7 3807. 76	99. 1226	8 3771. 12	99. 1454	9 3751. 83	99. 0963	10 3736. 4	99. 1728
11 3712. 3	99. 1375	12 3691. 09	99. 1278	13 3677. 59	99. 0668	14 3649. 62	98. 7307	15 3630. 34	98. 3238
16 3619. 73	98. 2246	17 3584. 06	95. 8666	18 3374. 82	94. 1237	19 2924. 52	93. 7919	20 2855. 1	95. 4801
21 2345. 98	99. 0692	22 2283. 3	98. 9748	23 1735. 62	98. 2665	24 1685. 48	98. 9656	25 1654. 62	98. 5348
26 1638. 23	98. 562	27 1560. 13	99. 3643	28 1542. 77	99. 4651	29 1509. 03	99. 5417	30 1458. 89	98. 2905
31 1375. 96	98. 571	32 1260. 25	97. 8404	33 1156. 12	98. 406	34 1071. 26	94. 9354	35 1037. 52	94. 8501
36 895. 773	100. 494	37 799. 35	98. 9379	38 650. 858	91. 2493				

Figure S32. IR spectrum of **5**

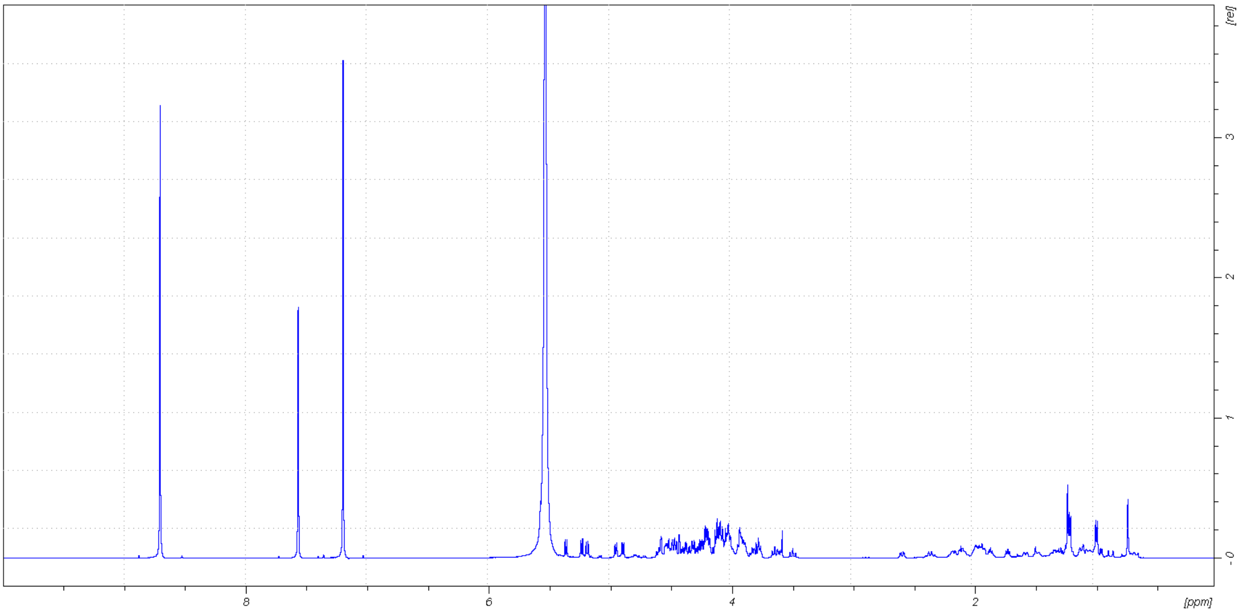

Figure S33. ¹H NMR spectrum of **5**

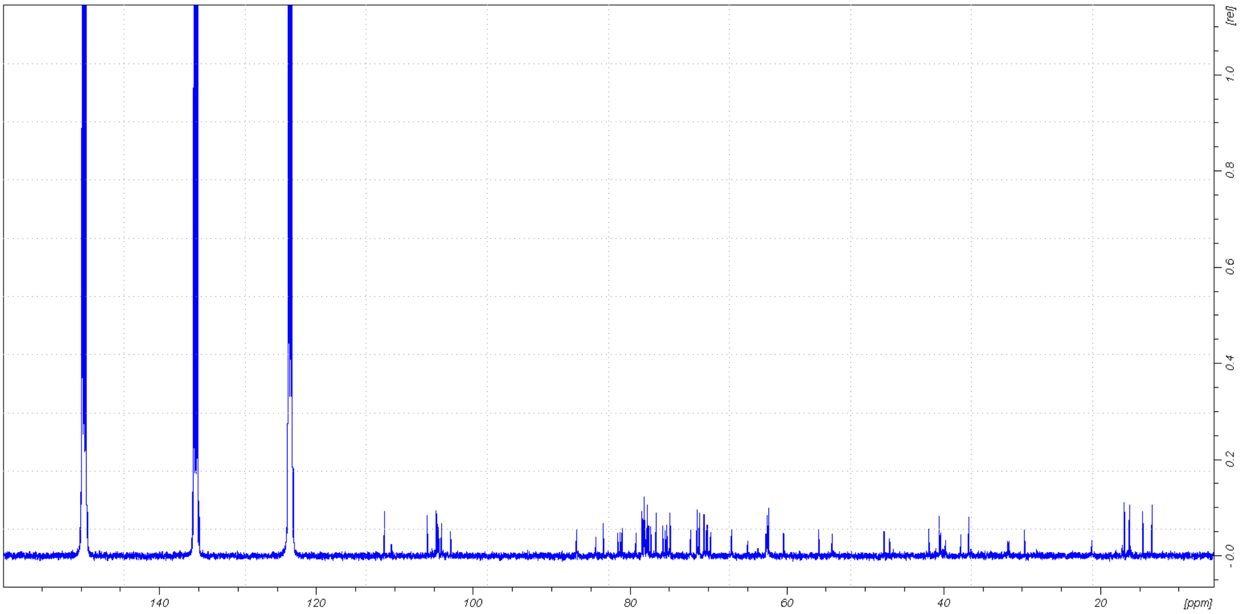

Figure S34. ¹³C NMR spectrum of **5**

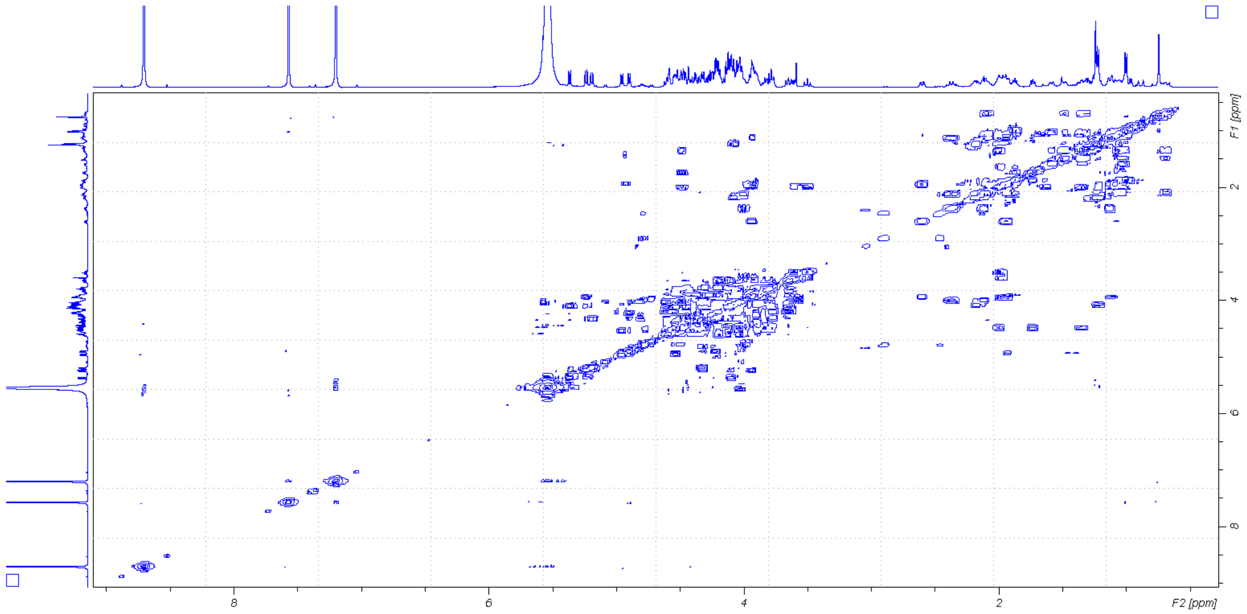

Figure S35. ^{1}H-^{1}H COSY spectrum of **5**

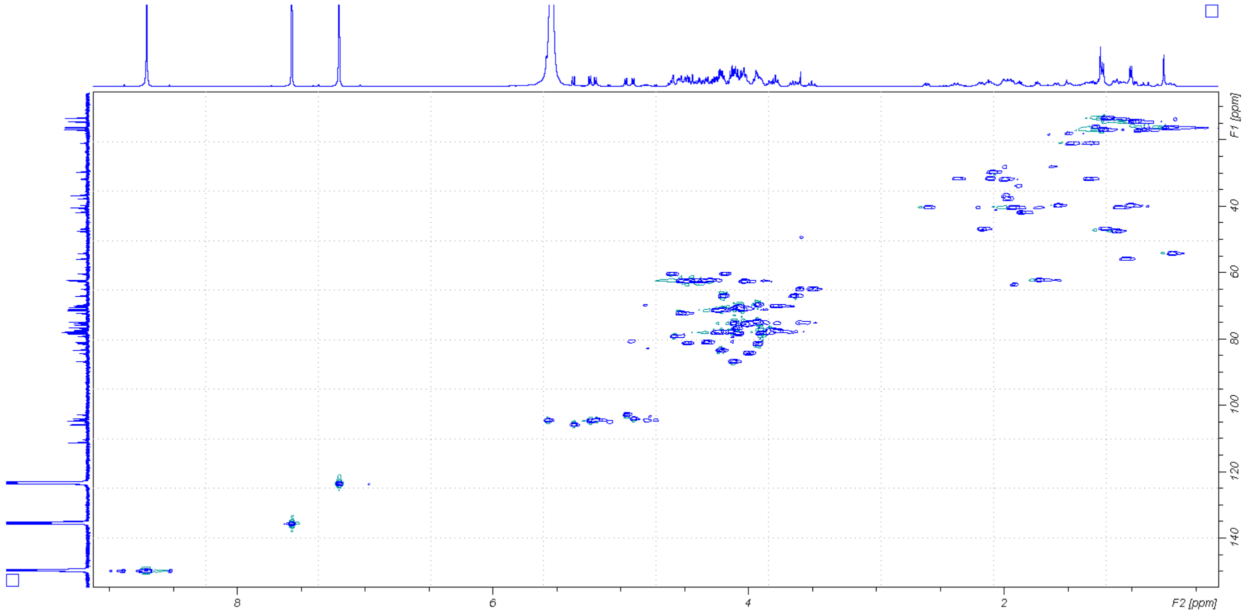

Figure S36. HSQC spectrum of **5**

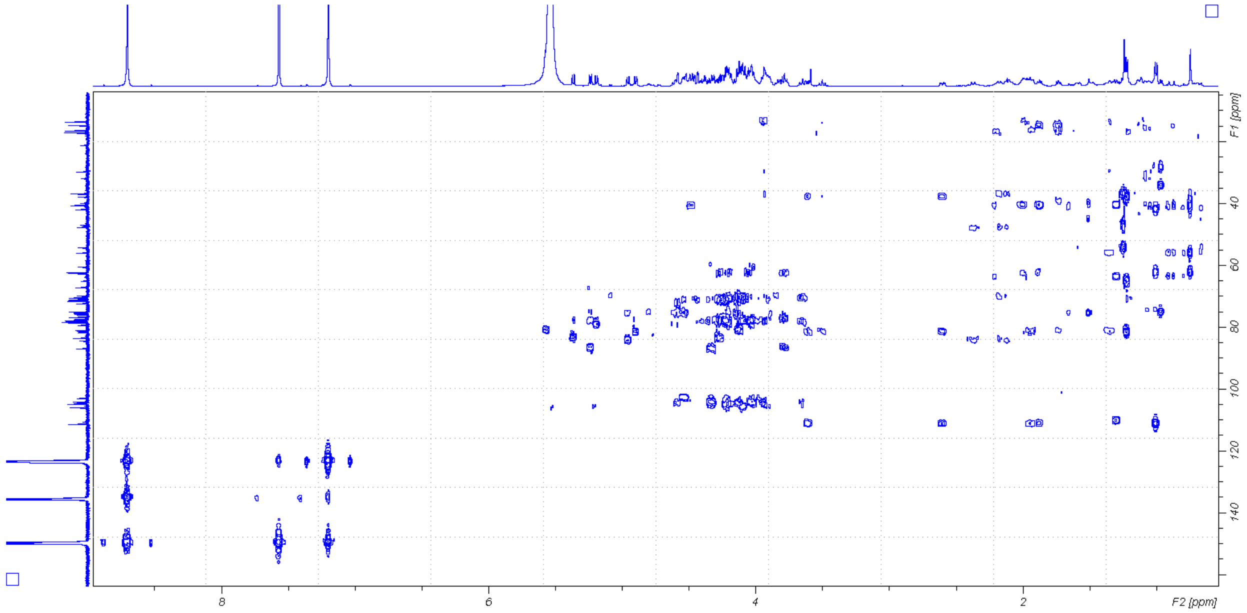

Figure S37. HMBC spectrum of **5**

Elemental Composition Report **Page 1**

Single Mass Analysis
Tolerance = 10.0 mDa / DBE: min = -1.5, max = 300.0
Element prediction: Off
Number of isotope peaks used for i-FIT = 3

Monoisotopic Mass, Even Electron Ions
5 formula(e) evaluated with 1 results within limits (up to 50 closest results for each mass)
Elements Used:
C: 0-300 H: 0-1000 O: 7-7 Na: 1-1
AGM-C1-4-3-2-4-2
M-18267 201 (2.038) AM2 (Ar,22000.0,0.00,0.00); ABS; Cm (178:208) 1: TOF MS ES+ 6.34e+007

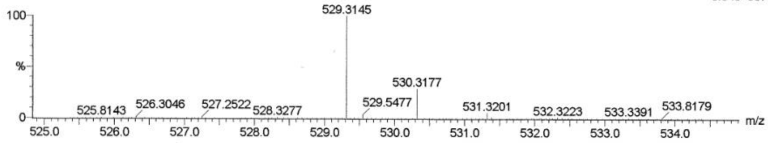

Minimum:				-1.5				
Maximum:		10.0	10.0	300.0				
Mass	Calc. Mass	mDa	PPM	DBE	i-FIT	Norm	Conf(%)	Formula
529.3145	529.3141	0.4	0.8	6.5	719.8	n/a	n/a	C29 H46 O7 Na

Figure S38. HRESITOFMS data of **6**

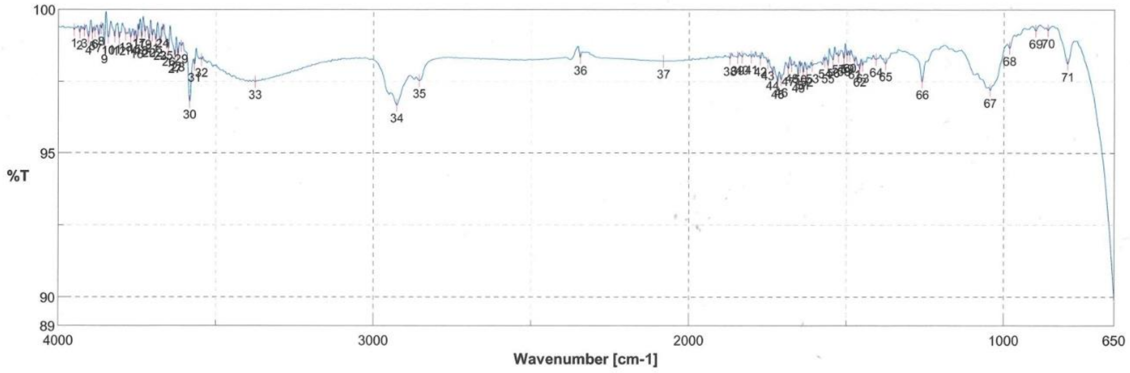

積算回数	40	分解	4 cm-1
ゼロフィリング	ON	アポダイゼーション	Cosine
ゲイン	Auto (1)	スキャンスピード	Auto (2 mm/sec)
測定日時	2021/06/08 14:34	更新日時	2021/06/08 14:35
測定者	Yuna Takahashi		
ファイル名	Memory#2		
サンプル名	AGM-21		
コメント	film		

No. cm-1	%T	No. cm-1	%T	No. cm-1	%T	No. cm-1	%T	No. cm-1	%T
1 3950. 46	99. 2879	2 3933. 11	99. 2062	3 3918. 64	99. 2761	4 3905. 15	99. 0243	5 3892. 61	99. 1784
6 3884. 9	99. 2512	7 3872. 36	99. 111	8 3864. 65	99. 3441	9 3855. 01	98. 7243	10 3842. 47	99. 0269
11 3822. 22	99. 0533	12 3807. 76	99. 0024	13 3787. 51	99. 1333	14 3771. 12	99. 0172	15 3759. 55	99. 0703
16 3751. 83	98. 8988	17 3745. 08	99. 28	18 3736. 4	99. 0139	19 3725. 8	99. 2332	20 3713. 26	98. 9382
21 3702. 66	99. 1057	22 3691. 09	98. 9421	23 3677. 59	98. 8357	24 3669. 87	99. 2712	25 3657. 34	98. 8446
26 3650. 59	98. 628	27 3630. 34	98. 3797	28 3620. 7	98. 4394	29 3610. 09	98. 7327	30 3584. 06	96. 7952
31 3568. 63	98. 0864	32 3546. 45	98. 2507	33 3375. 78	97. 4851	34 2926. 45	96. 662	35 2855. 1	97. 5215
36 2345. 02	98. 3349	37 2081. 78	98. 2036	38 1870. 61	98. 2994	39 1846. 51	98. 3326	40 1832. 04	98. 3445
41 1803. 12	98. 3478	42 1774. 19	98. 2887	43 1751. 05	98. 146	44 1735. 62	97. 8106	45 1719. 23	97. 5016
46 1708. 62	97. 559	47 1686. 44	97. 9468	48 1675. 84	98. 0564	49 1654. 62	97. 7171	50 1647. 87	98. 024
51 1638. 23	97. 8216	52 1627. 63	97. 9268	53 1610. 27	98. 056	54 1570. 74	98. 2219	55 1560. 13	98. 0471
56 1543. 74	98. 2472	57 1526. 38	98. 3837	58 1509. 03	98. 2984	59 1499. 38	98. 3989	60 1490. 7	98. 4175

No. cm-1	%T	No. cm-1	%T	No. cm-1	%T	No. cm-1	%T	No. cm-1	%T
61 1475. 28	98. 2055	62 1458. 89	97. 9319	63 1450. 21	98. 0648	64 1407. 78	98. 267	65 1376. 93	98. 1238
66 1260. 25	97. 4899	67 1044. 26	97. 1939	68 983. 518	98. 6588	69 899. 63	99. 2666	70 862. 989	99. 2784
71 799. 35	98. 1136								

Figure S39. IR spectrum of **6**

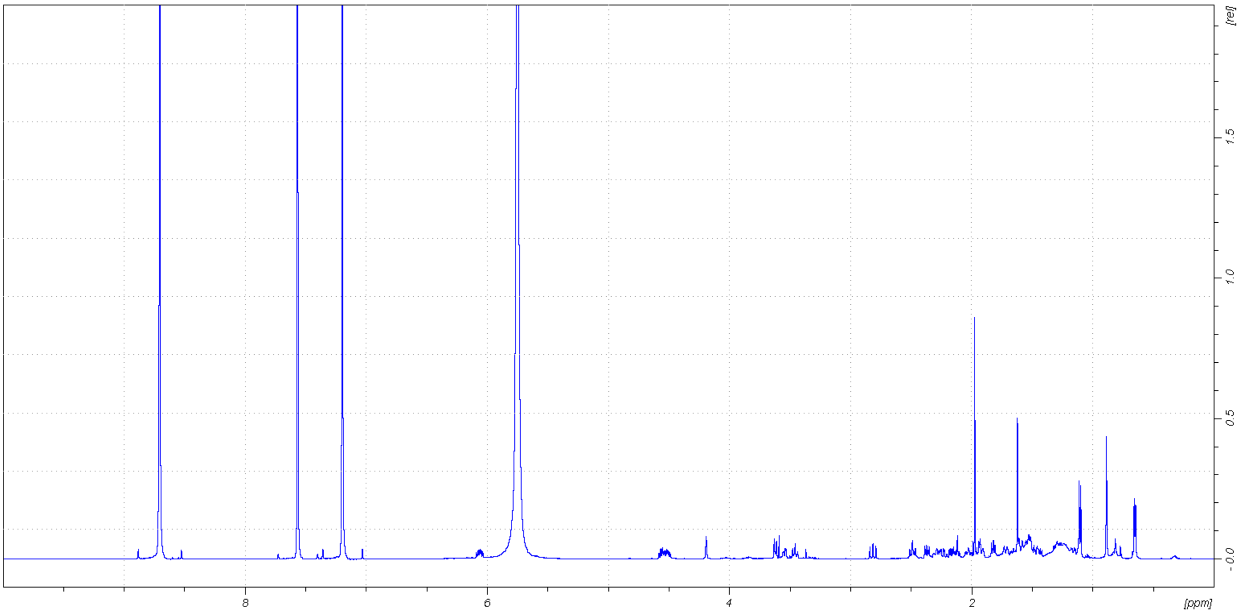

Figure S40. ^{1}H NMR spectrum of **6**

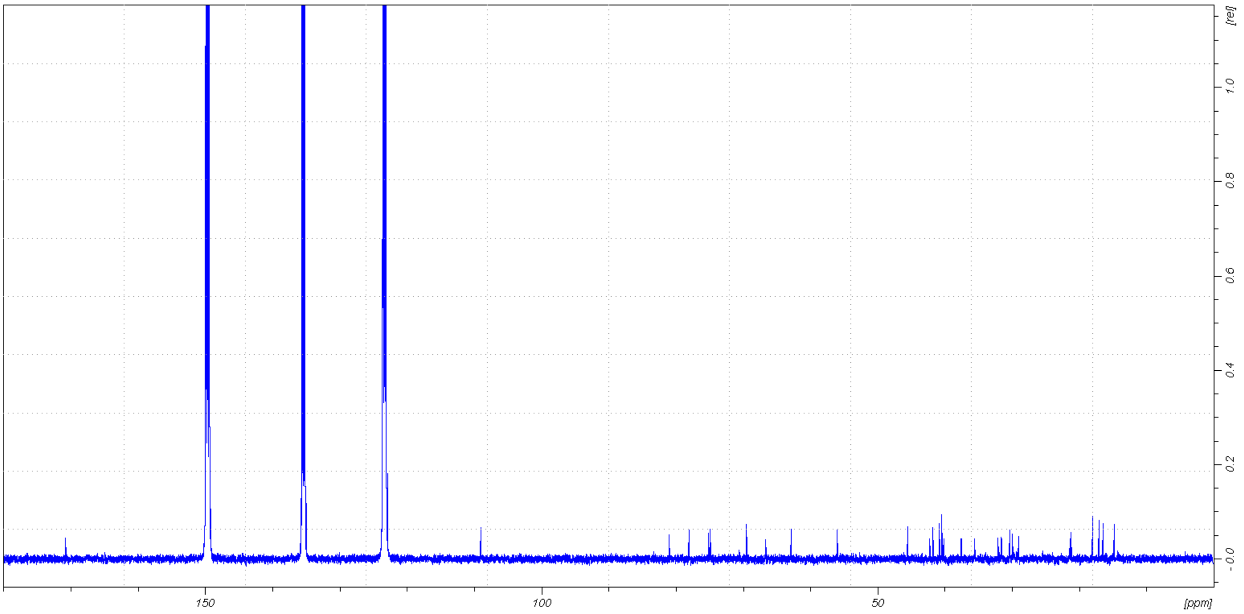

Figure S41. ^{13}C NMR spectrum of **6**

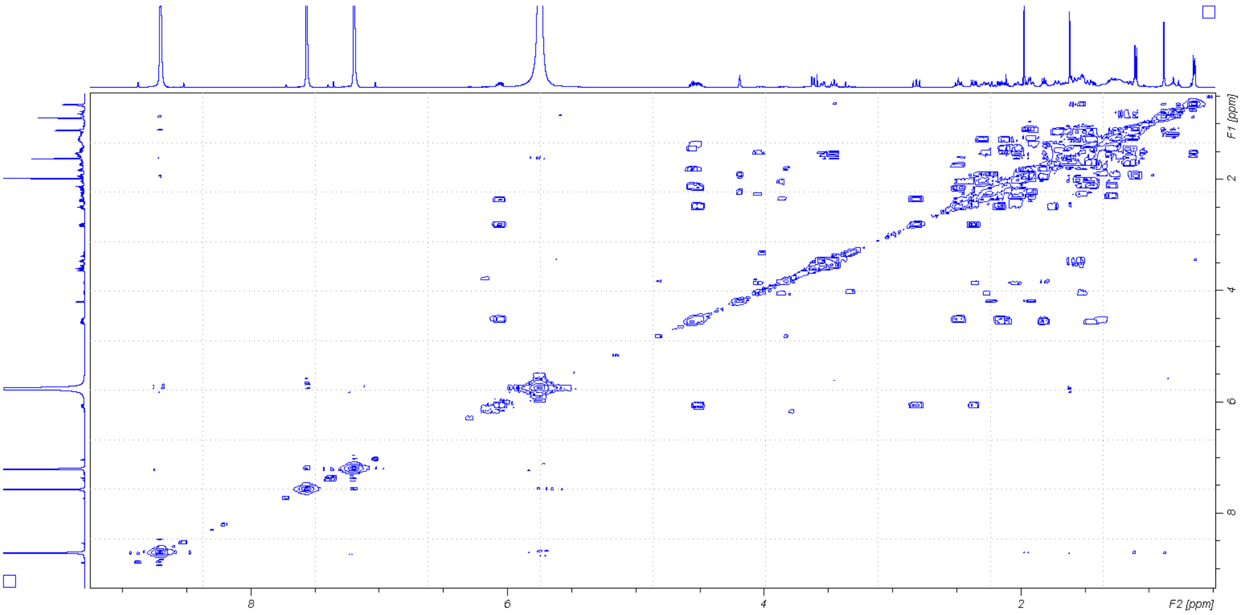

Figure S42. ^1H-^1H COSY spectrum of **6**

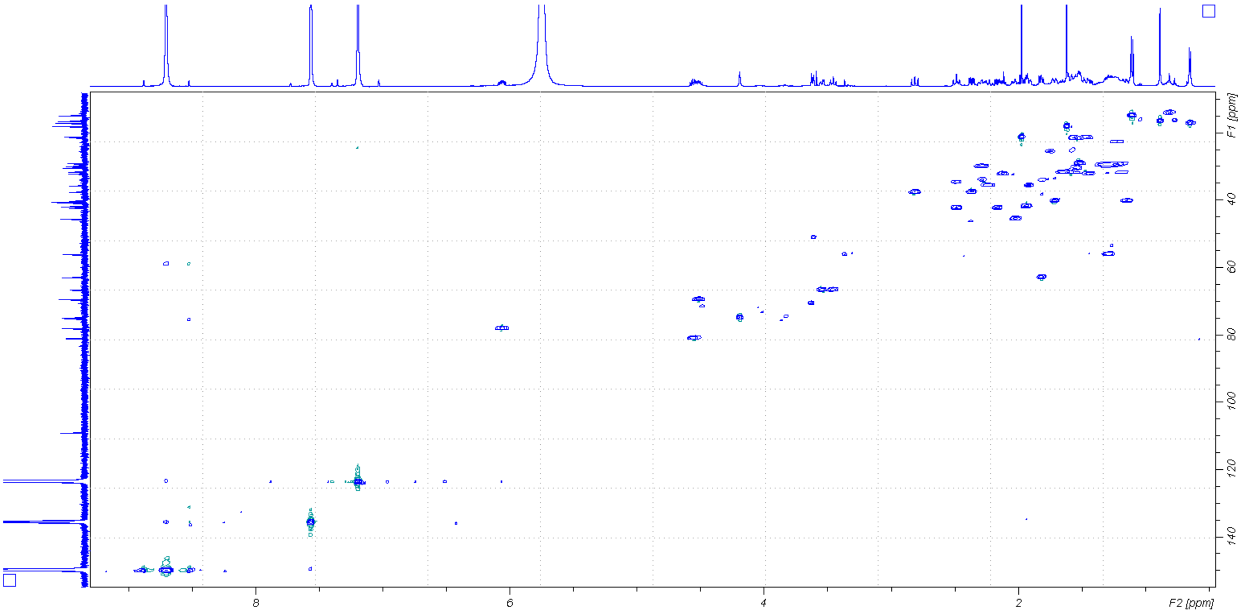

Figure S43. HSQC spectrum of **6**

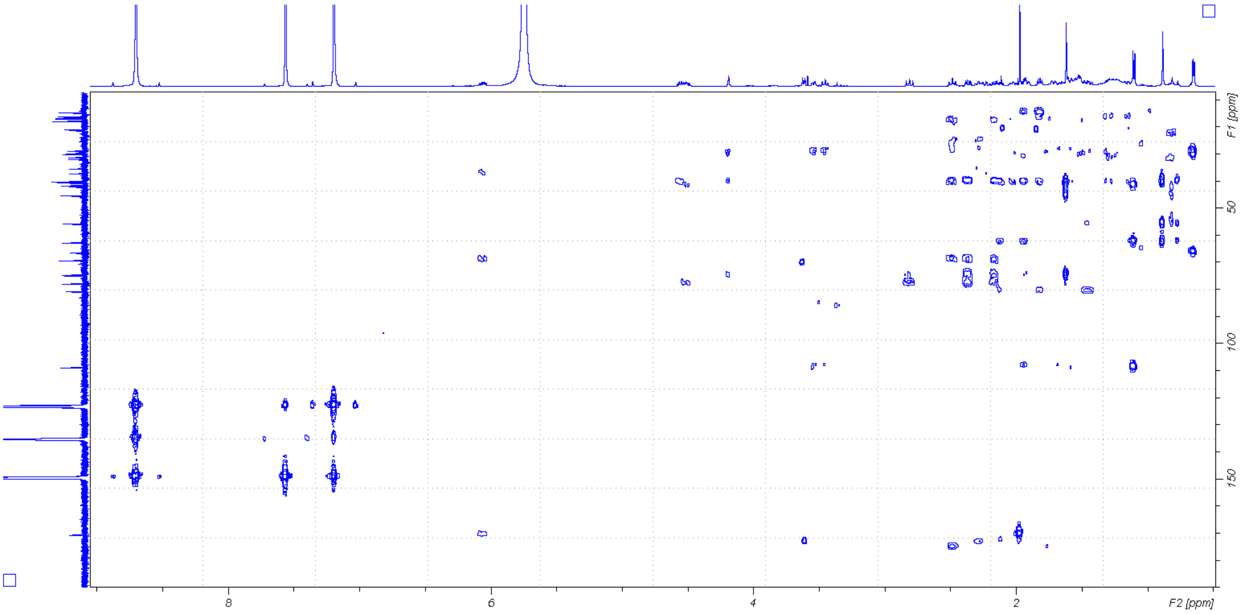

Figure S44. HMBC spectrum of **6**

Elemental Composition Report Page 1

Single Mass Analysis
Tolerance = 10.0 mDa / DBE: min = -1.5, max = 300.0
Element prediction: Off
Number of isotope peaks used for i-FIT = 3

Monoisotopic Mass, Even Electron Ions
10 formula(e) evaluated with 1 results within limits (up to 50 closest results for each mass)
Elements Used:
C: 0-300 H: 0-3000 O: 28-28 Na: 1-1
AGM-C5-4-1-2-6-3
M-19025 631 (6.332) AM2 (Ar,22000.0,0.00,0.00); ABS; Cm (631:659) 1: TOF MS ES+ 6.54e+007

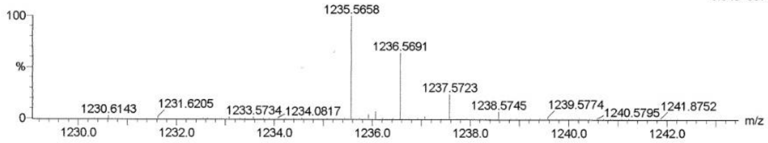

Minimum:				-1.5				
Maximum:		10.0	5.0	300.0				
Mass	Calc. Mass	mDa	PPM	DBE	i-FIT	Norm	Conf(%)	Formula
1235.5658	1235.5673	-1.5	-1.2	10.5	357.0	n/a	n/a	C56 H92 O28 Na

Figure S45. HRESITOFMS data of **7**

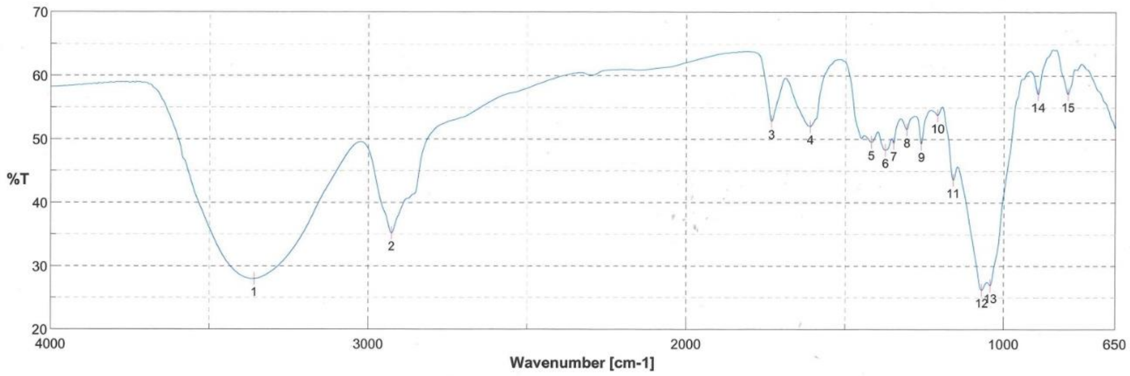

積算回数	40	分解	4 cm-1
ゼロフィリング	ON	アポダイゼーション	Cosine
ゲイン	Auto (2)	スキャンスピード	Auto (2 mm/sec)
測定日時	2011/12/30 4:44	更新日時	2011/12/30 4:46
測定者	yamamoto kie		
ファイル名	Memory#1		
サンプル名	AGM-C5-4-1-2-6-3		
コメント	film		

No.	cm-1	%T	No.	cm-1	%T	No.	cm-1	%T	No.	cm-1	%T	No.	cm-1	%T
1	3358.43	27.9677	2	2926.45	35.1516	3	1731.76	52.8299	4	1611.23	51.9995	5	1418.39	49.5457
6	1374.03	48.3154	7	1348	49.4192	8	1306.54	51.5471	9	1260.25	49.2885	10	1209.15	53.7654
11	1159.97	43.5887	12	1069.33	26.2412	13	1041.37	26.9631	14	893.844	57.1124	15	799.35	57.1268

Figure S46. IR spectrum of **7**

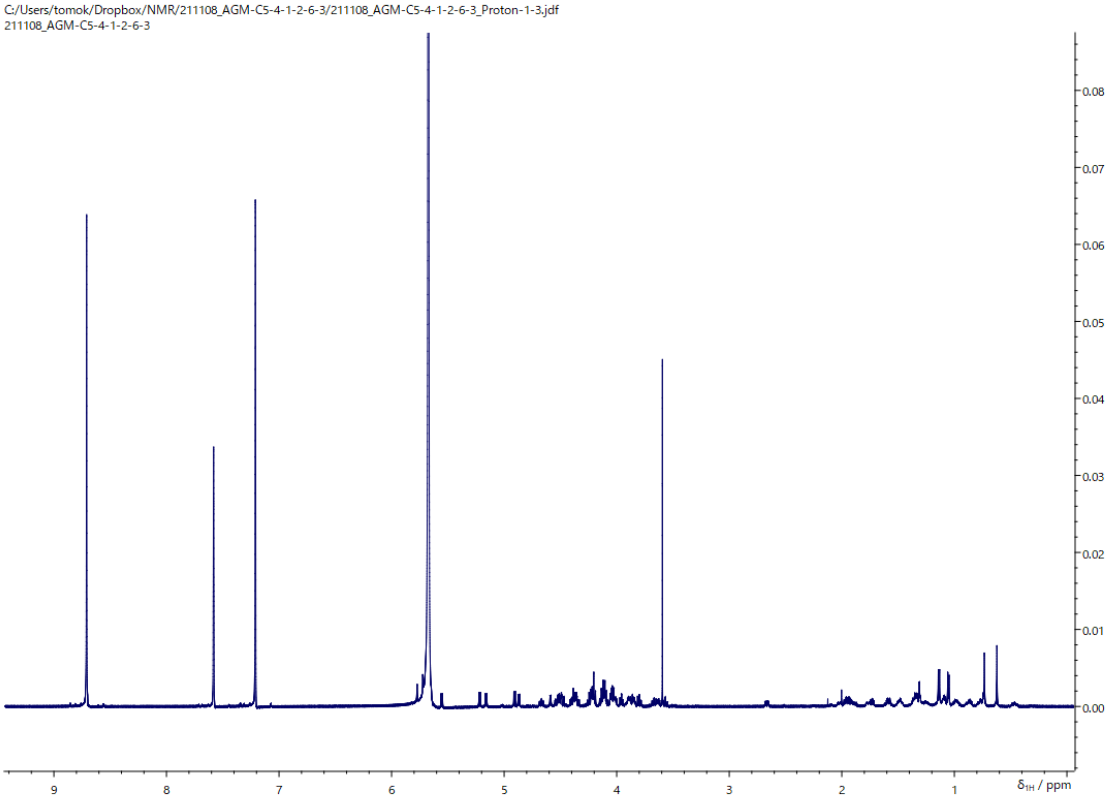

Figure S47. ^{1}H NMR spectrum of **7**

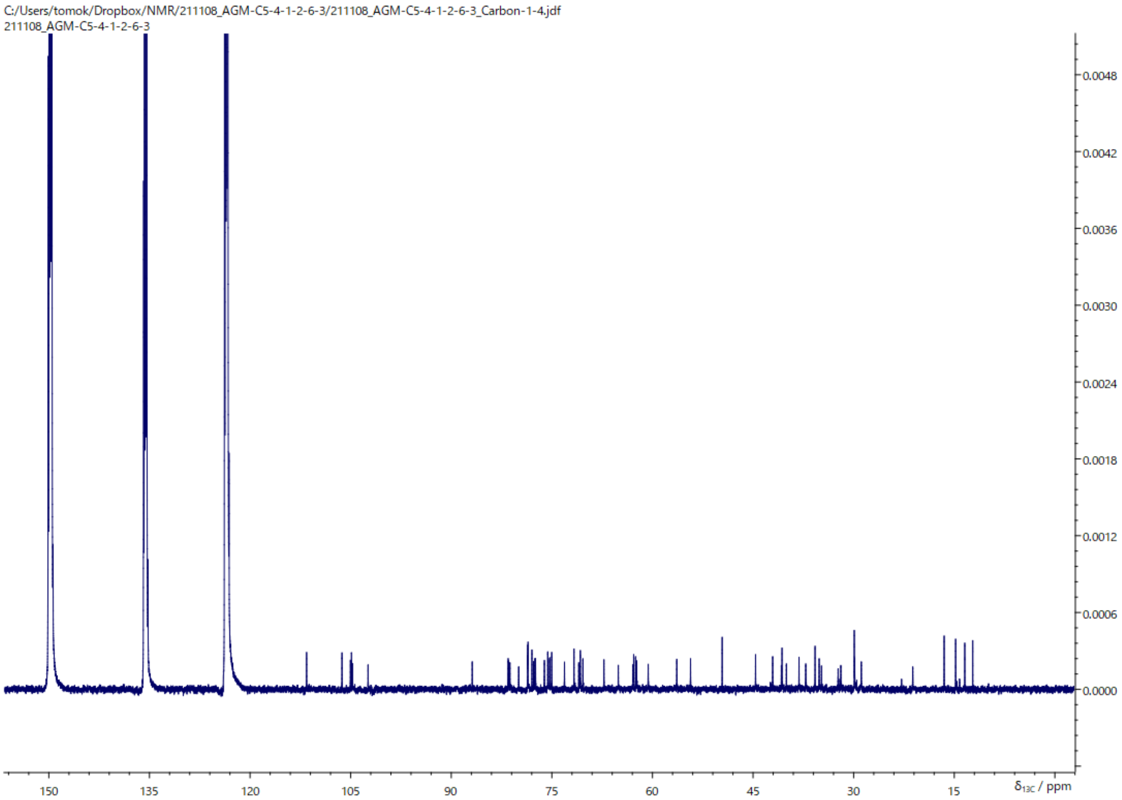

Figure S48. ^{13}C NMR spectrum of **7**

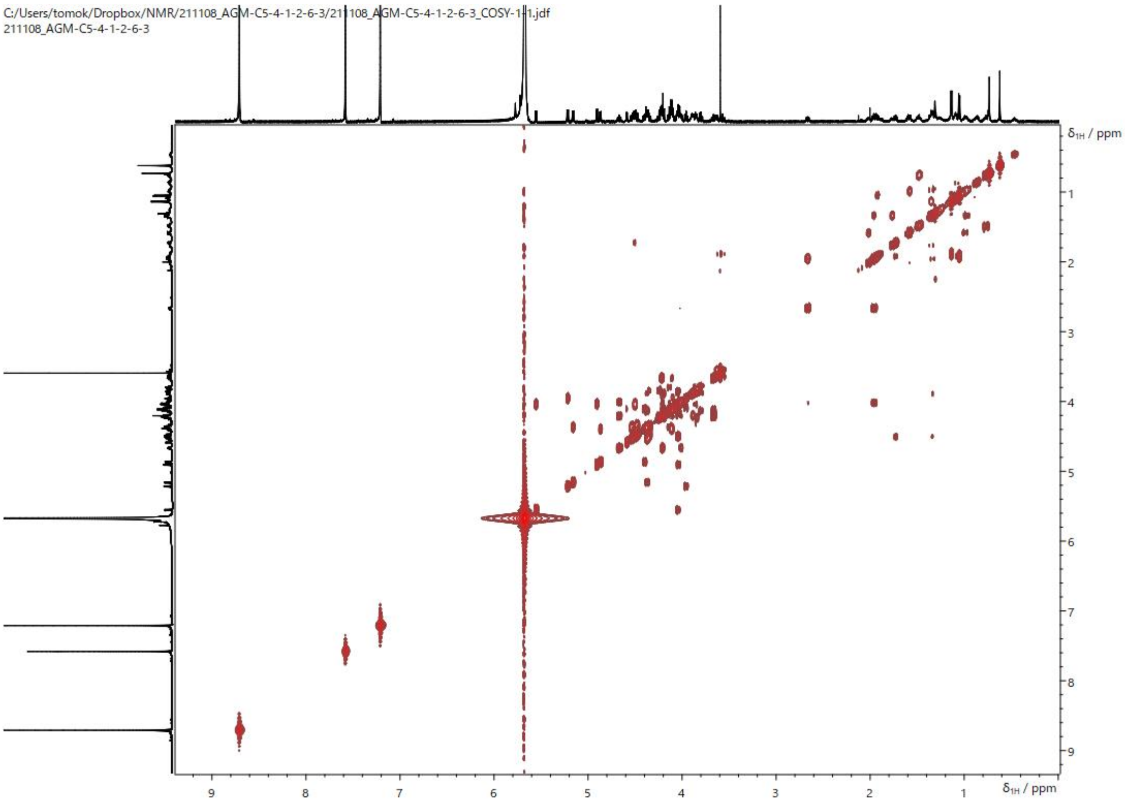

Figure S49. ^{1}H-^{1}H COSY spectrum of **7**

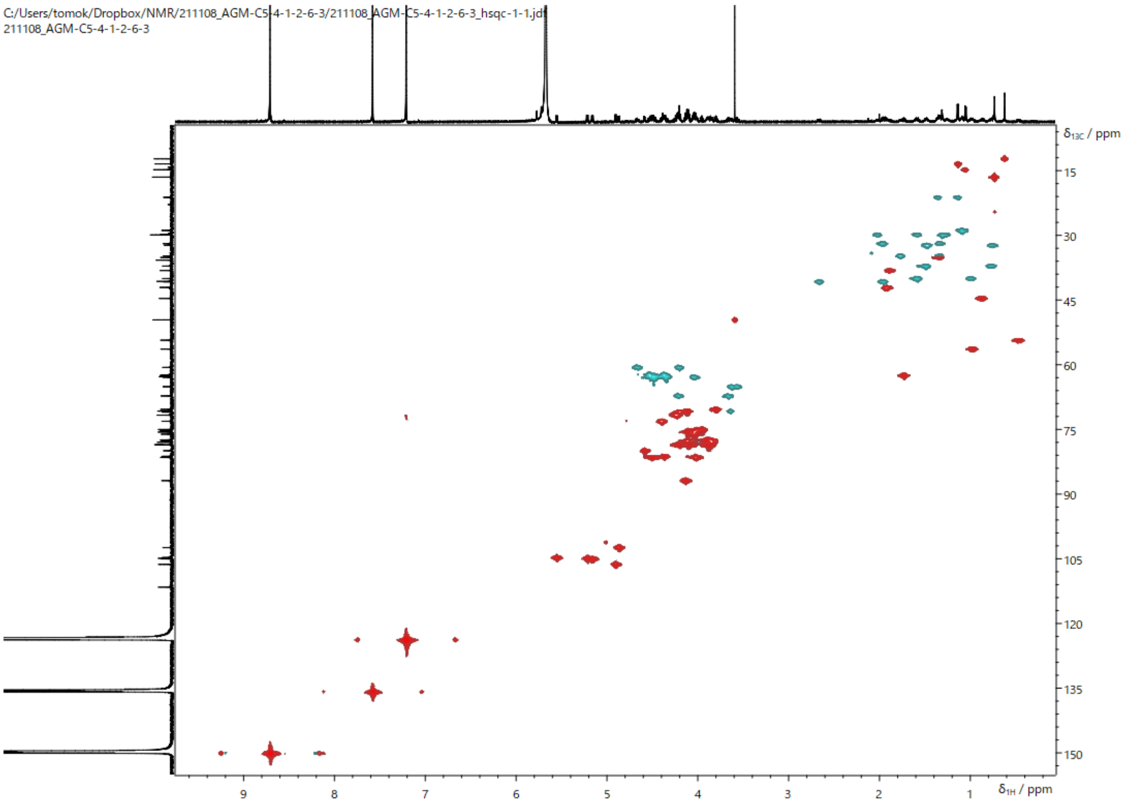

Figure S50. HSQC spectrum of **7**

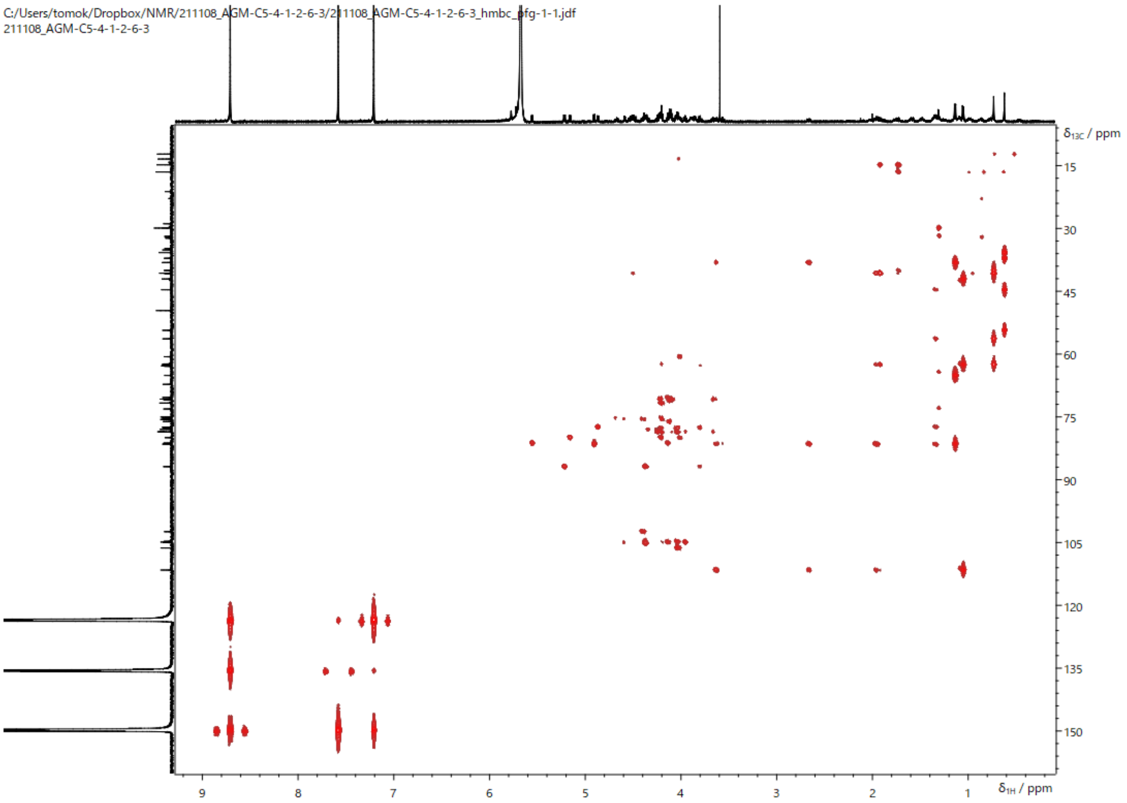

Figure S51. HMBC spectrum of **7**

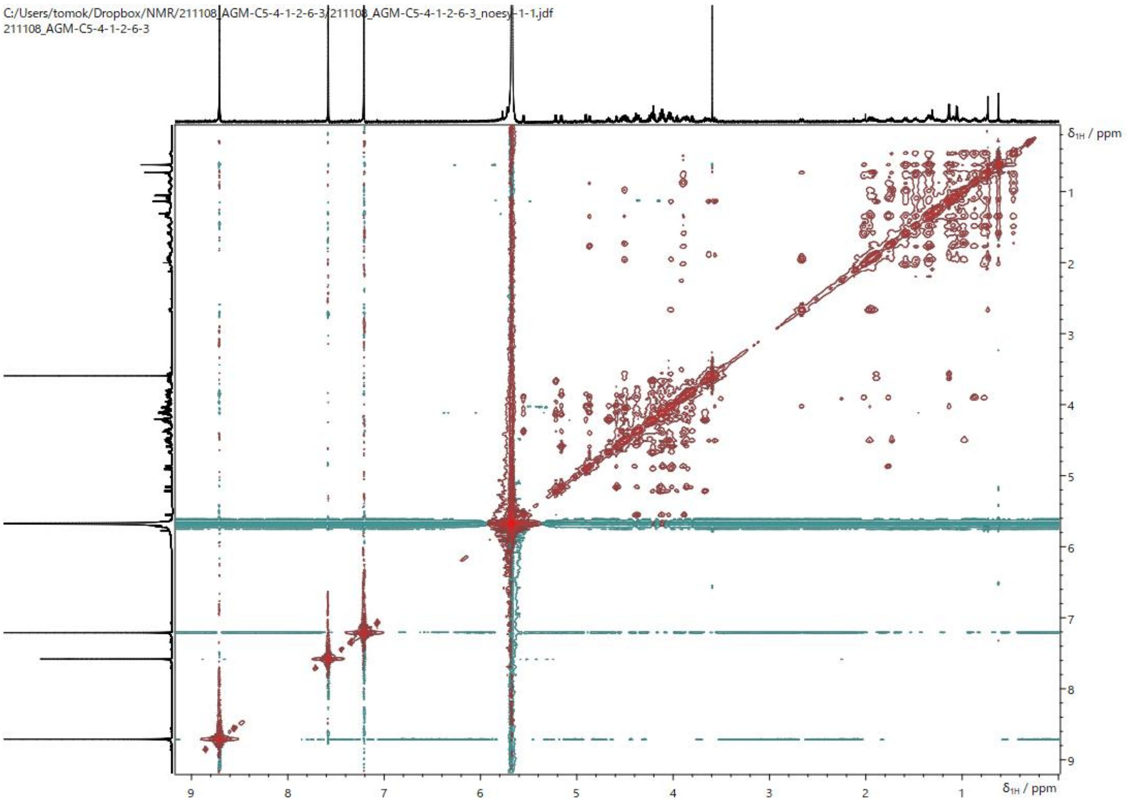

Figure S52. NOESY spectrum of **7**

Elemental Composition Report **Page 1**

Single Mass Analysis
Tolerance = 10.0 mDa / DBE: min = -1.5, max = 300.0
Element prediction: Off
Number of isotope peaks used for i-FIT = 3

Monoisotopic Mass, Even Electron Ions
7 formula(e) evaluated with 1 results within limits (up to 50 closest results for each mass)
Elements Used:
C: 0-100 H: 0-3000 O: 13-13 Na: 1-1
AGM-C3-7-3
M-19542 81 (0.837) AM2 (Ar,22000.0,0.00,0.00); ABS; Cm (81:112) 1: TOF MS ES+ 9.23e+007

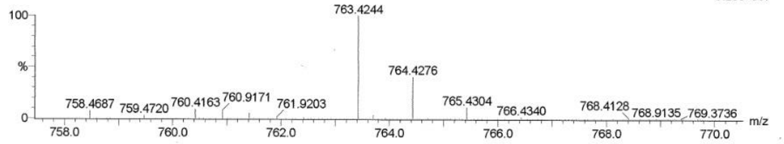

Mass	Calc. Mass	mDa	PPM	DBE	i-FIT	Norm	Conf(%)	Formula
Minimum:				-1.5				
Maximum:		10.0	5.0	300.0				
763.4244	763.4245	-0.1	-0.1	7.5	589.5	n/a	n/a	C39 H64 O13 Na

Figure S53. HRESITOFMS data of **8**

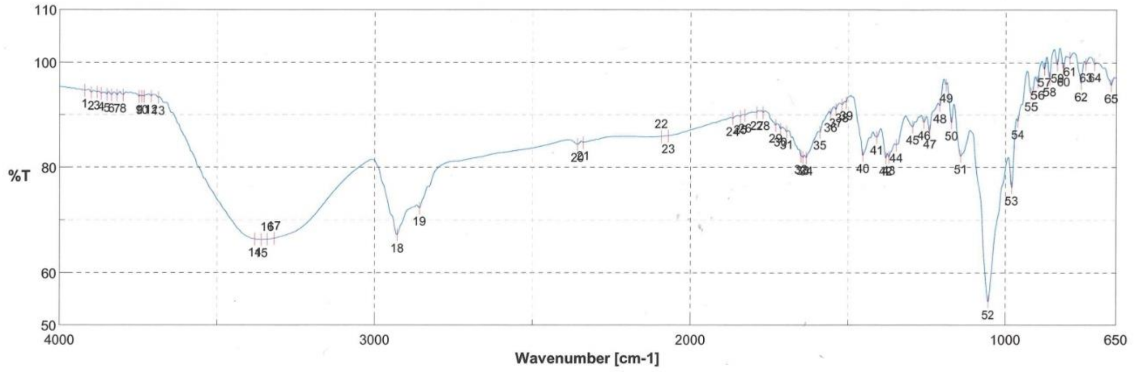

積算回数	40	分解	4 cm-1
ゼロフィリング	ON	アポダイゼーション	Cosine
ゲイン	Auto (4)	スキャンスピード	Auto (2 mm/sec)
測定日時	2011/11/24 21:26	更新日時	2011/11/24 21:30
測定者	Yamamoto		
ファイル名	Memory#2		
サンプル名	AGM-27		
コメント	film		

No.	cm-1	%T	No.	cm-1	%T	No.	cm-1	%T	No.	cm-1	%T	No.	cm-1	%T
1	3920.57	94.6026	2	3900.32	94.2194	3	3882	94.2855	4	3868.5	94.0337	5	3850.18	93.8485
6	3836.68	93.7559	7	3818.36	93.6996	8	3799.08	93.7477	9	3747.98	93.448	10	3740.26	93.5076
11	3732.55	93.504	12	3709.41	93.5369	13	3687.23	93.2817	14	3380.6	66.3478	15	3360.35	66.216
16	3341.07	66.2928	17	3319.86	66.4736	18	2929.34	67.1441	19	2858.95	72.2432	20	2358.52	84.3149
21	2340.19	84.6769	22	2093.35	85.8732	23	2071.17	85.9831	24	1866.76	89.2971	25	1842.65	89.6546
26	1828.19	89.931	27	1790.58	90.4083	28	1770.33	90.4372	29	1730.8	88.0185	30	1715.37	87.2943
31	1696.09	86.7763	32	1650.77	82.0884	33	1644.98	81.8693	34	1634.38	81.8133	35	1590.99	86.7008
36	1557.24	90.0962	37	1539.88	90.9812	38	1520.6	91.9486	39	1507.1	92.3575	40	1454.06	82.2595
41	1410.67	85.7234	42	1381.75	81.7683	43	1372.1	81.9221	44	1348.96	84.2604	45	1296.89	87.7152
46	1260.25	88.5596	47	1241.93	86.9095	48	1210.11	91.755	49	1190.83	95.6845	50	1173.47	88.4849
51	1143.58	82.1332	52	1055.84	54.4587	53	981.59	76.108	54	962.305	88.7529	55	918.914	94.086
56	899.63	96.2722	57	879.381	98.7367	58	863.953	96.9173	59	838.883	99.518	60	818.634	98.8369

No.	cm-1	%T	No.	cm-1	%T	No.	cm-1	%T	No.	cm-1	%T	No.	cm-1	%T
61	798.385	100.81	62	762.709	95.9513	63	747.281	99.614	64	718.354	99.6394	65	666.285	95.6064

Figure S54. IR spectrum of **8**

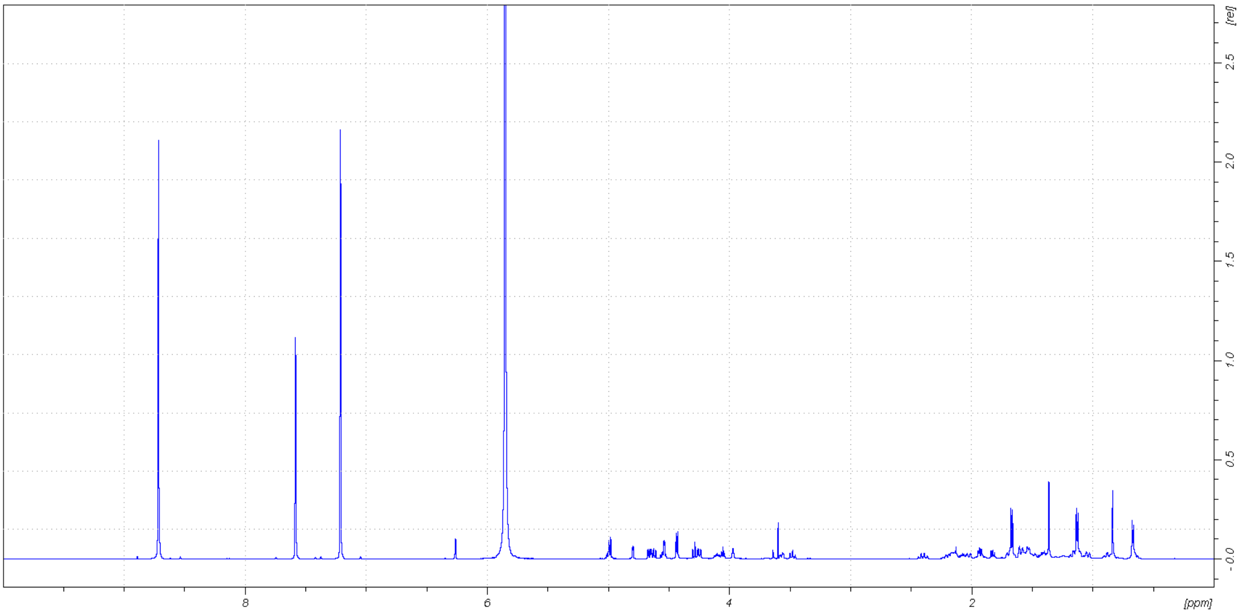

Figure S55. ^{1}H NMR spectrum of **8**

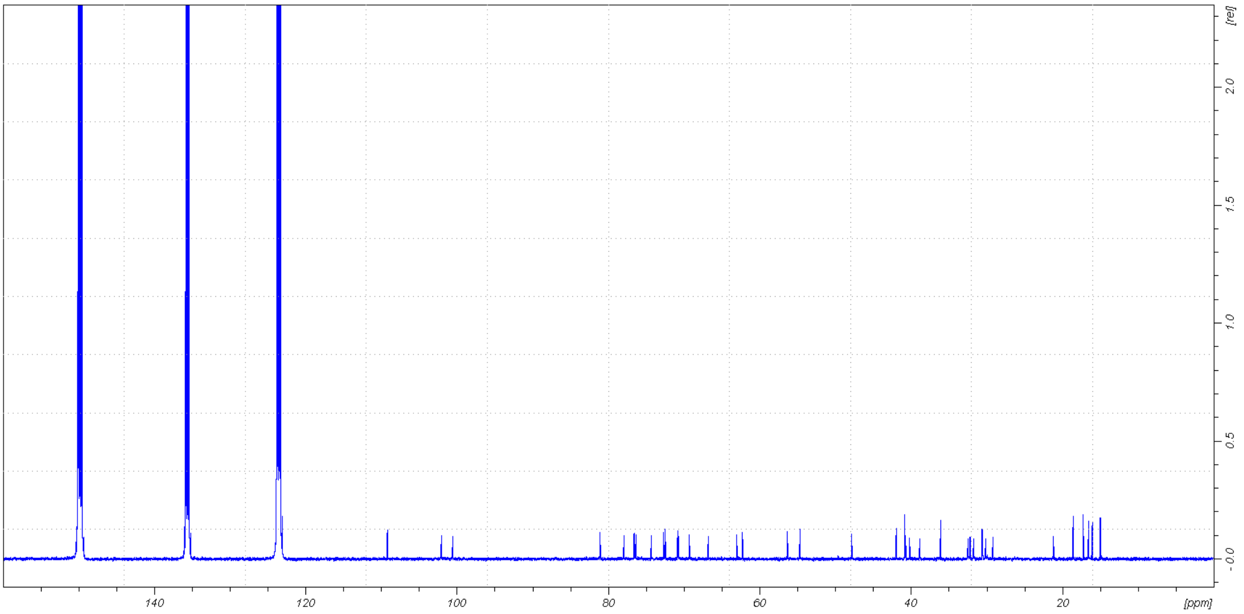

Figure S56. ^{13}C NMR spectrum of **8**

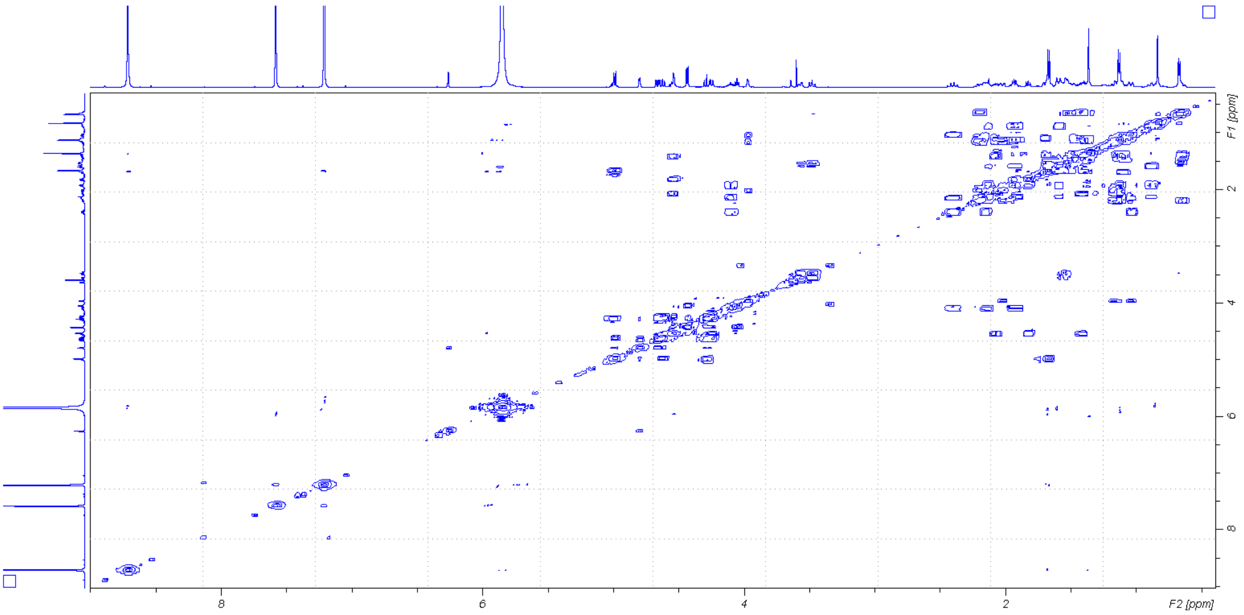

Figure S57. ^1H-^1H COSY spectrum of **8**

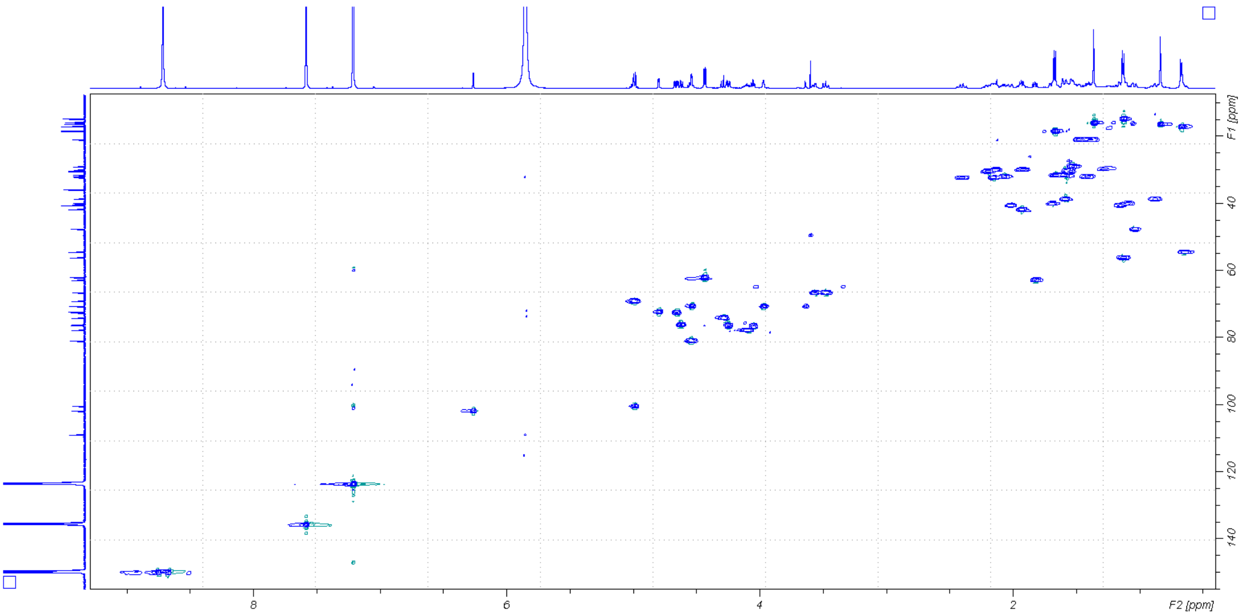

Figure S58. HSQC spectrum of **8**

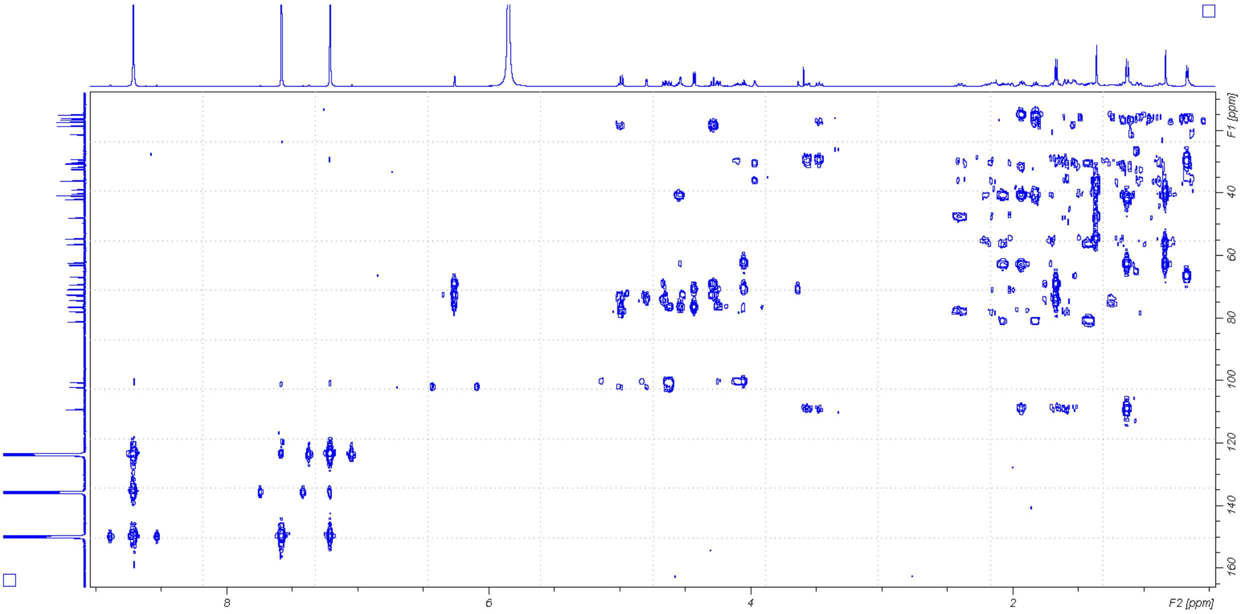

Figure S59. HMBC spectrum of **8**

Elemental Composition Report Page 1

Single Mass Analysis
Tolerance = 10.0 mDa / DBE: min = -1.5, max = 300.0
Element prediction: Off
Number of isotope peaks used for i-FIT = 3

Monoisotopic Mass, Even Electron Ions
6 formula(e) evaluated with 1 results within limits (up to 50 closest results for each mass)
Elements Used:
C: 1-300 H: 1-1000 O: 12-12 Na: 1-1
AGM-B1-7-5-1
M-17252 336 (2.693) AM2 (Ar,22000.0,0.00,0.00); ABS; Cm (311:350) 1: TOF MS ES+ 2.12e+007

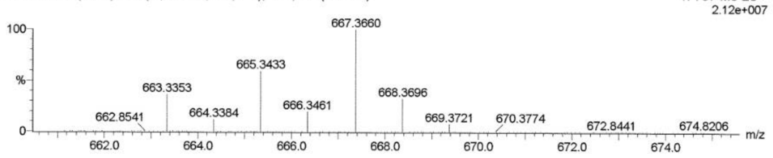

Minimum:				-1.5				
Maximum:		10.0	50.0	300.0				
Mass	Calc. Mass	mDa	PPM	DBE	i-FIT	Norm	Conf(%)	Formula
667.3660	667.3669	-0.9	-1.3	5.5	460.1	n/a	n/a	C33 H56 O12 Na

Figure S60. HRESITOFMS data of **9**

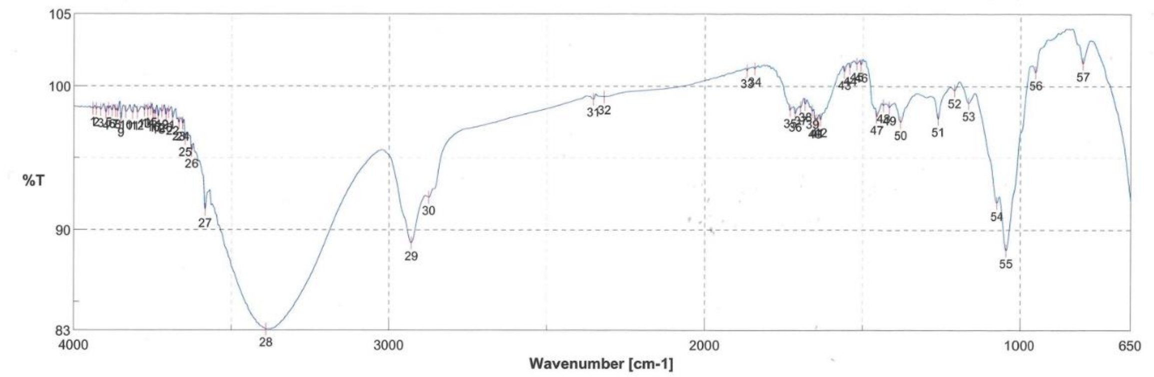

積算回数	40	分解	4 cm-1
ゼロフィリング	ON	アポダイゼーション	Cosine
ゲイン	Auto (1)	スキャンスピード	Auto (2 mm/sec)
測定日時	2021/06/08 14:05	更新日時	2021/06/08 14:06
測定者	Yuna Takahashi		
ファイル名	Memory#2		
サンプル名	AGM-15		
コメント	film		

No.	cm-1	%T	No.	cm-1	%T	No.	cm-1	%T	No.	cm-1	%T	No.	cm-1	%T
1	3939. 86	98. 3996	2	3929. 25	98. 3676	3	3914. 79	98. 3184	4	3898. 4	98. 1444	5	3889. 72	98. 3726
6	3879. 11	98. 2699	7	3868. 5	98. 2579	8	3861. 75	98. 2293	9	3851. 15	97. 6636	10	3835. 72	98. 1805
11	3814. 51	98. 1104	12	3799. 08	98. 1382	13	3776. 9	98. 2897	14	3765. 33	98. 3214	15	3756. 65	98. 3818
16	3748. 94	98. 0367	17	3742. 19	98. 01	18	3732. 55	97. 8714	19	3720. 98	98. 2258	20	3708. 44	97. 9715
21	3699. 76	98. 1885	22	3687. 23	97. 7784	23	3666. 98	97. 4116	24	3654. 44	97. 3927	25	3646. 73	96. 3094
26	3626. 48	95. 503	27	3584. 06	91. 3698	28	3389. 28	83. 0756	29	2929. 34	89. 0831	30	2874. 38	92. 2434
31	2353. 69	99. 0919	32	2318. 98	99. 2518	33	1866. 76	101. 094	34	1842. 65	101. 218	35	1730. 8	98. 3298
36	1714. 41	98. 0807	37	1697. 05	98. 5157	38	1682. 59	98. 7644	39	1659. 45	98. 28	40	1651. 73	97. 5897
41	1644. 98	97. 6404	42	1634. 38	97. 6802	43	1557. 24	100. 987	44	1539. 88	101. 259	45	1519. 63	101. 562
46	1506. 13	101. 493	47	1455. 03	97. 854	48	1434. 78	98. 6554	49	1416. 46	98. 5075	50	1379. 82	97. 4851
51	1260. 25	97. 7066	52	1209. 15	99. 715	53	1163. 83	98. 826	54	1073. 19	91. 8717	55	1042. 34	88. 569
56	948. 806	100. 967	57	801. 278	101. 591									

Figure S61. IR spectrum of **9**

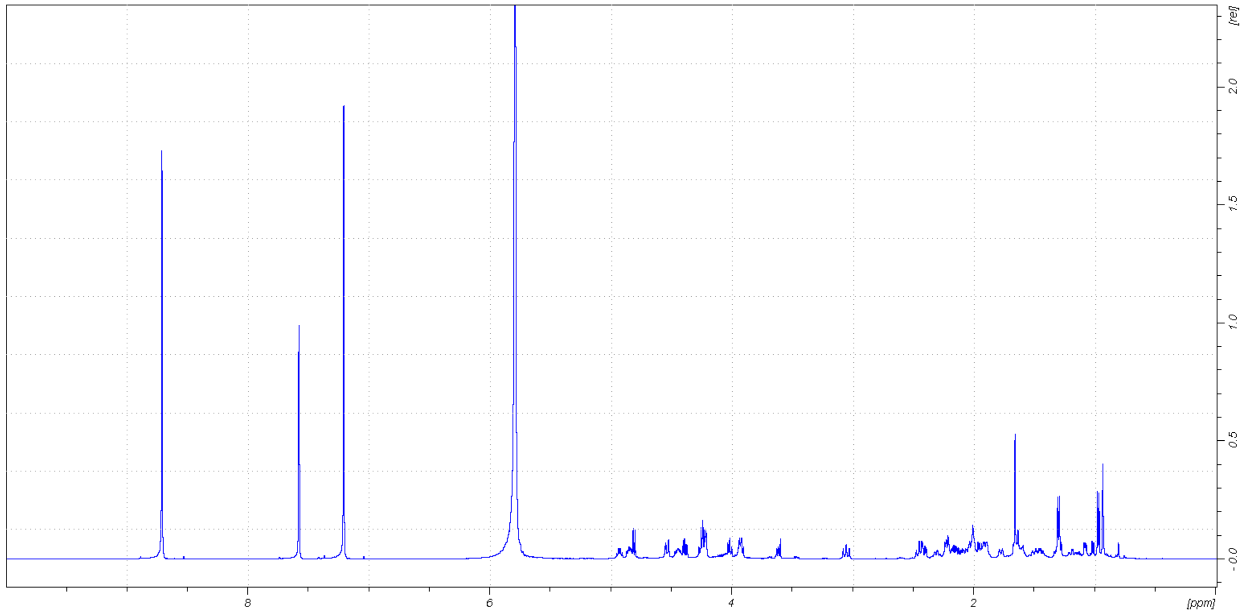

Figure S62. ^{1}H NMR spectrum of **9**

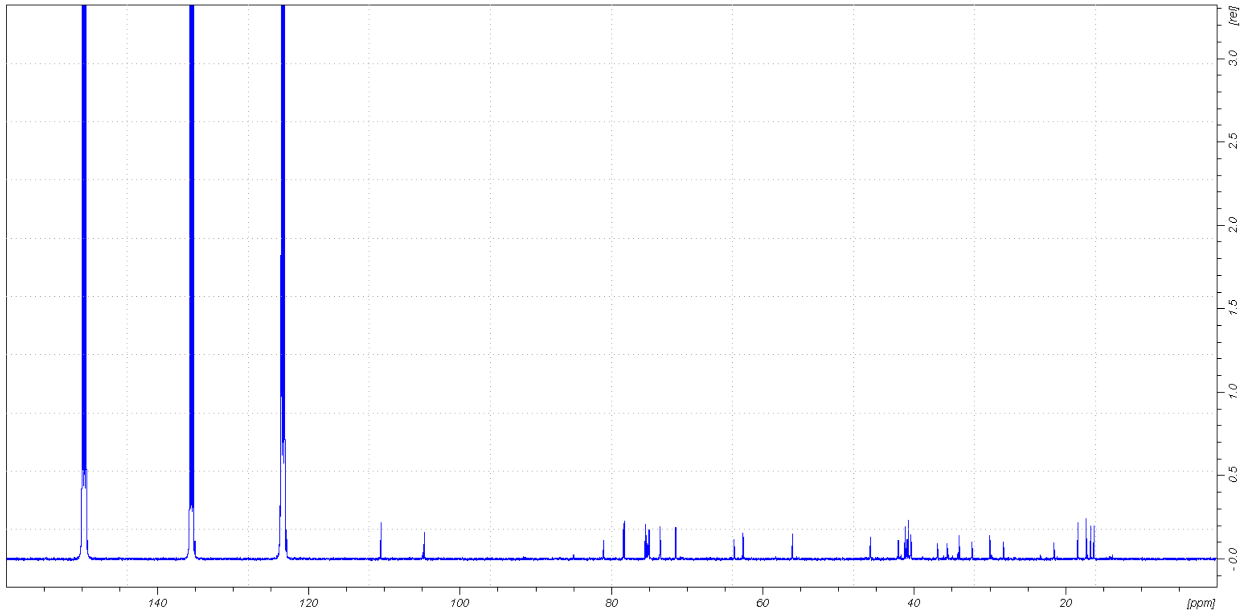

Figure S63. ^{13}C NMR spectrum of **9**

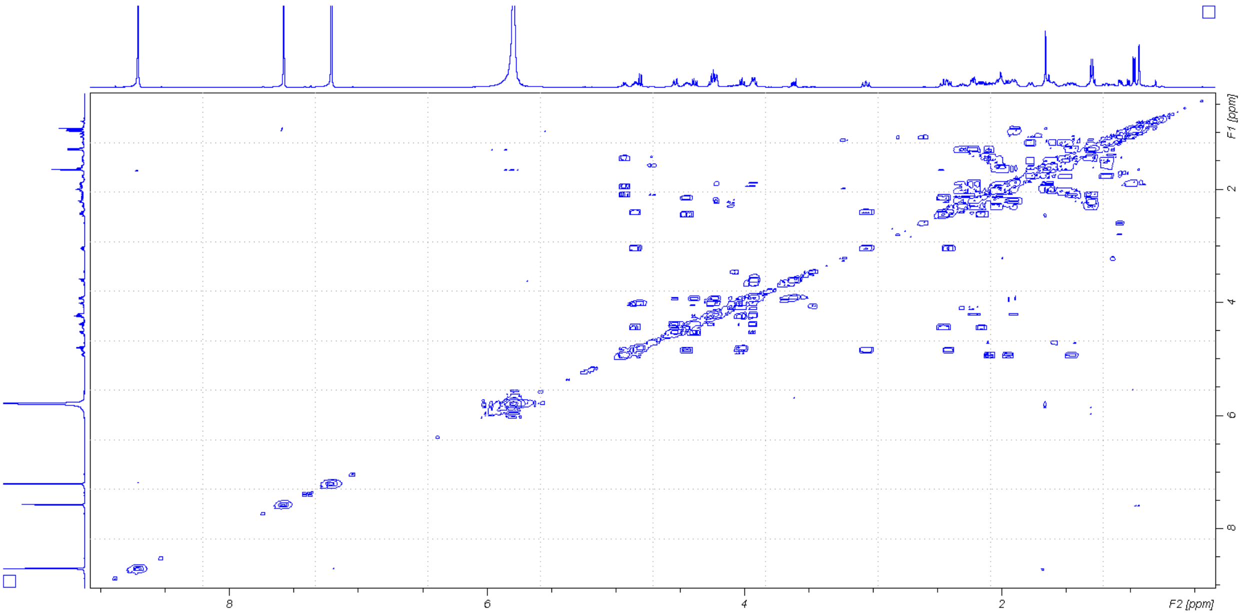

Figure S64. ^1H-^1H COSY spectrum of **9**

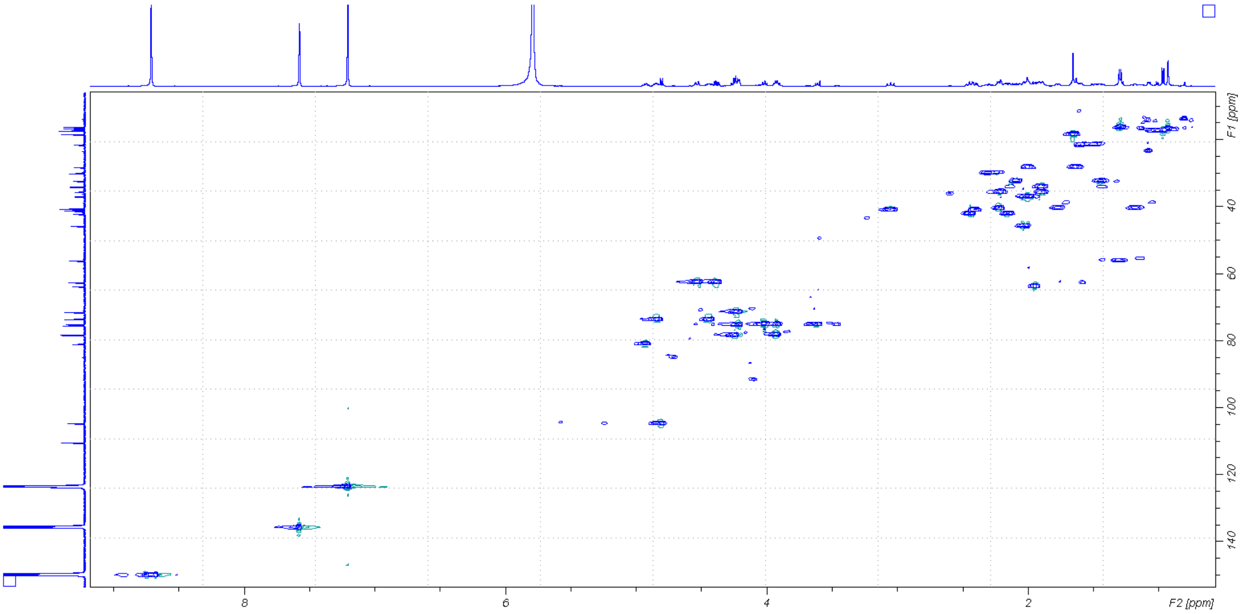

Figure S65. HSQC spectrum of **9**

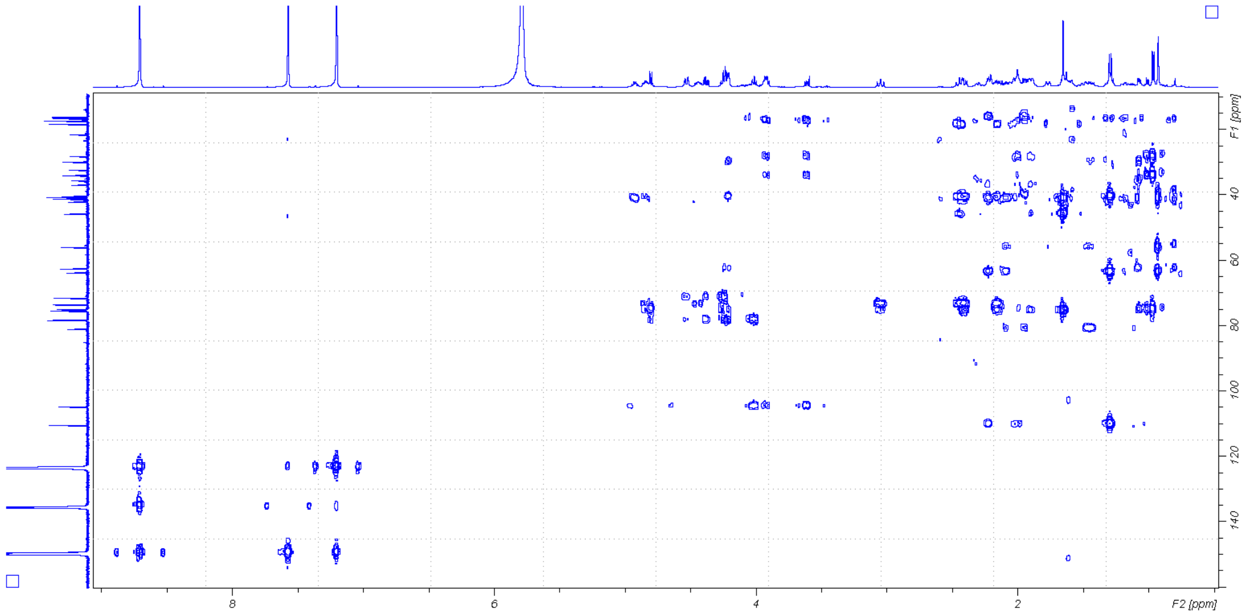

Figure S66. HMBC spectrum of **9**

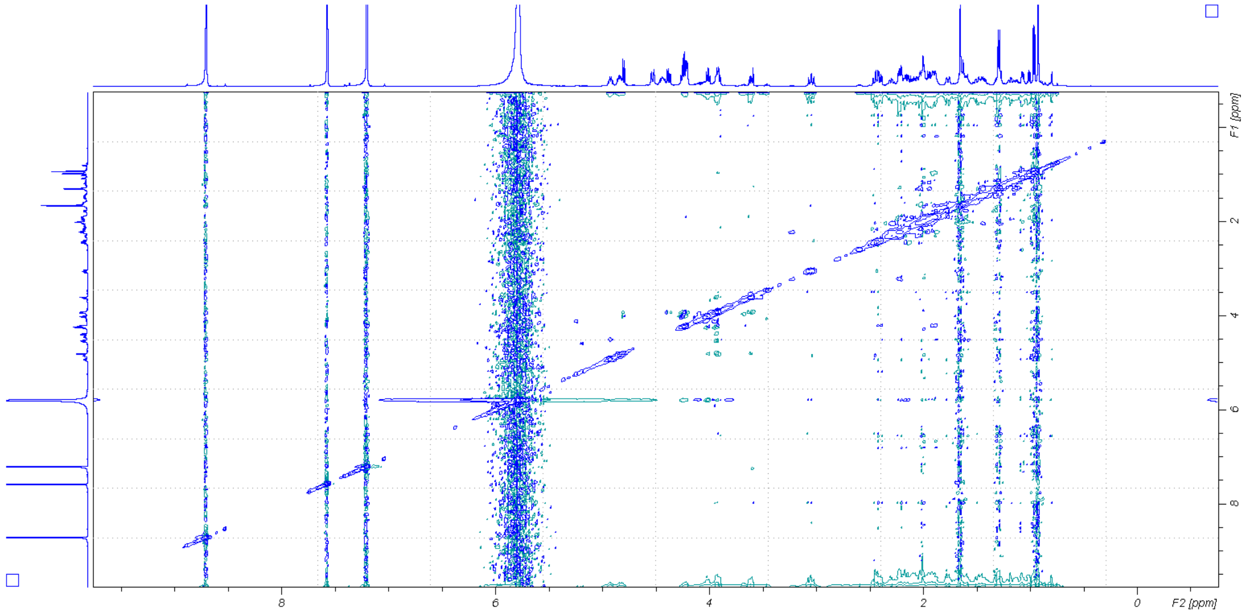

Figure S67. NOESY spectrum of **9**

Elemental Composition Report Page 1

Single Mass Analysis
Tolerance = 10.0 mDa / DBE: min = -1.5, max = 300.0
Element prediction: Off
Number of isotope peaks used for i-FIT = 3

Monoisotopic Mass, Even Electron Ions
8 formula(e) evaluated with 1 results within limits (up to 50 closest results for each mass)
Elements Used:
C: 1-300 H: 1-1000 O: 23-23 Na: 1-1

AGM-B2-5-7-2-3
M-16682 432 (3.081) AM2 (Ar,22000.0,0.00,0.00); ABS; Cm (430:473) 1: TOF MS ES+ 1.30e+006

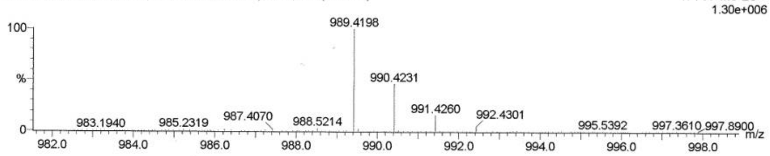

	Mass	Calc. Mass	mDa	PPM	DBE	i-FIT	Norm	Conf(%)	Formula
Minimum:					-1.5				
Maximum:			10.0	50.0	300.0				
	989.4198	989.4206	-0.8	-0.8	9.5	332.4	n/a	n/a	C44 H70 O23 Na

Figure S68. HRESITOFMS data of **10**

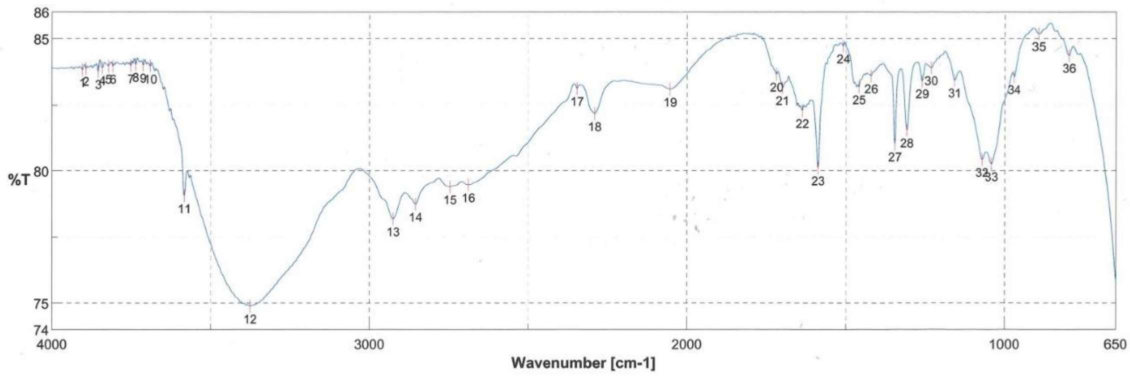

積算回数	40	分解	4 cm-1
ゼロフィリング	ON	アポダイゼーション	Cosine
ゲイン	Auto (1)	スキャンスピード	Auto (2 mm/sec)
測定日時	2021/03/01 15:17	更新日時	2021/03/01 15:20
測定者	Yuna Takahashi		
ファイル名	AGM-8 2回目.jws		
サンプル名	AGM-8 2回目		
コメント	film		

No.	cm-1	%T	No.	cm-1	%T	No.	cm-1	%T	No.	cm-1	%T	No.	cm-1	%T
1	3904.18	83.8373	2	3892.61	83.8806	3	3854.04	83.7641	4	3841.51	83.9125	5	3822.22	83.9649
6	3807.76	83.97	7	3751.83	83.9776	8	3736.4	84.0328	9	3712.3	84.0072	10	3690.12	83.9686
11	3583.09	79.0608	12	3374.82	74.8839	13	2925.48	78.183	14	2855.1	78.745	15	2746.14	79.3975
16	2688.28	79.4753	17	2345.98	83.1188	18	2291.02	82.1683	19	2053.82	83.0947	20	1718.26	83.6616
21	1699.94	83.1548	22	1637.27	82.2846	23	1588.09	80.1153	24	1508.06	84.7281	25	1458.89	83.1816
26	1421.28	83.5971	27	1346.07	81.0187	28	1308.46	81.5506	29	1259.29	83.4076	30	1231.33	83.9015
31	1157.08	83.4399	32	1071.26	80.4352	33	1040.41	80.2752	34	970.019	83.5686	35	892.88	85.1939
36	797.421	84.3705												

Figure S69. IR spectrum of **10**

ピーク検出 - 2021_03_05-3.jws

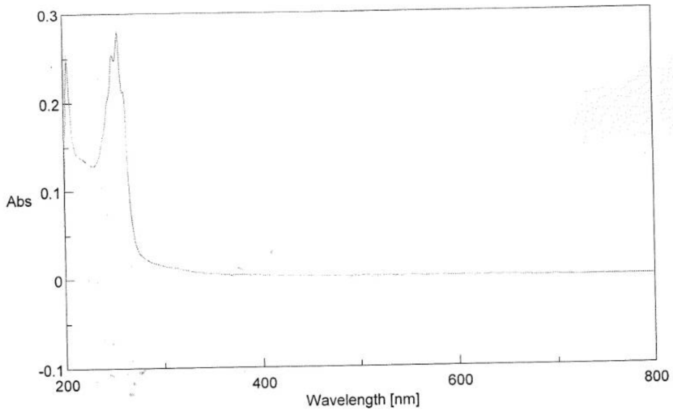

[コメント情報]

試料名	AGM-B2-5-7-2-3
コメント	1/10希釈
測定者	Yuna Takahashi
所属	
会社	東京薬科大学 漢方エ

[データ情報]

作成日時	2021/03/05 20:14
データタイプ	等間隔データ
横軸	Wavelength [nm]
縦軸	Abs
スタート	800 nm
エンド	200 nm
データ間隔	0.2 nm
データ数	3001

[測定情報]

装置名	V-630
機種名	V-630
シリアル番号	A017661148
付属品名	USE-753
付属品シリアルNo.	A017661148
セル長	10 mm
測光モード	Abs
測定範囲	800 - 200 nm
データ取込間隔	0.2 nm
UV/Vis バンド幅	1.5 nm
レスポンス	Fast
走査速度	100 nm/min
光源切換	340 nm
光源	D2/WI
フィルタ切換	ステップ
補正	ベースライン

[ピーク検出結果]

No.	位置	強度	No.	位置	強度	No.	位置	強度
1	255.4	0.279596	2	203.6	0.247403			

Figure S70. UV spectrum of **10**

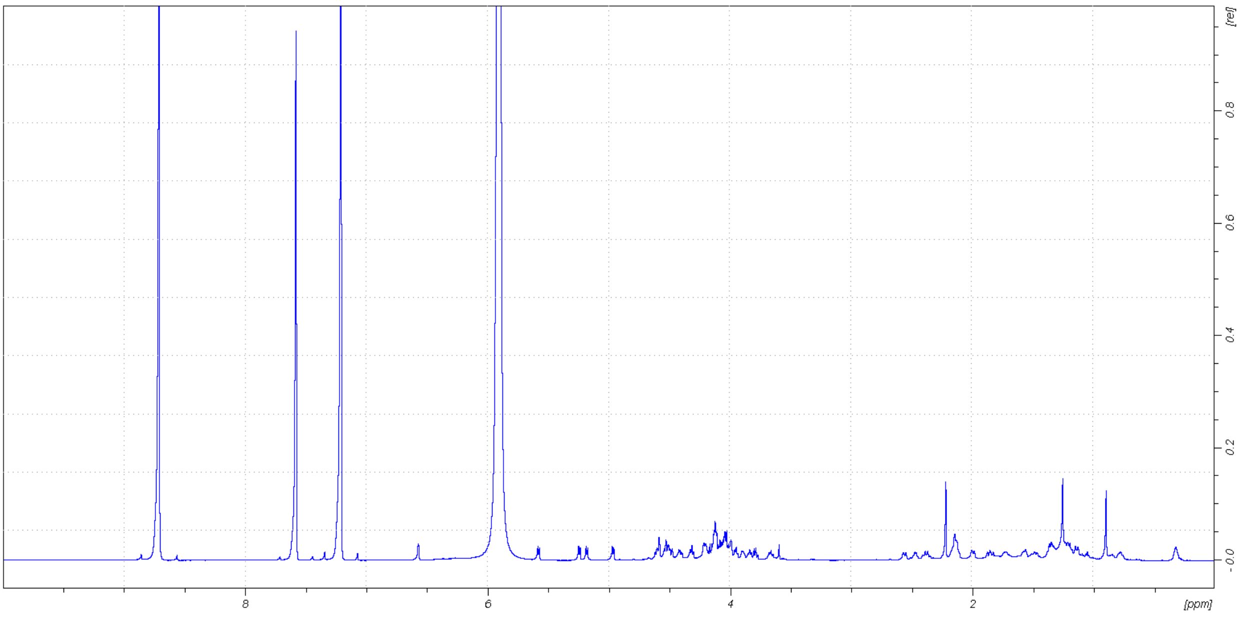

Figure S71. ^{1}H NMR spectrum of **10**

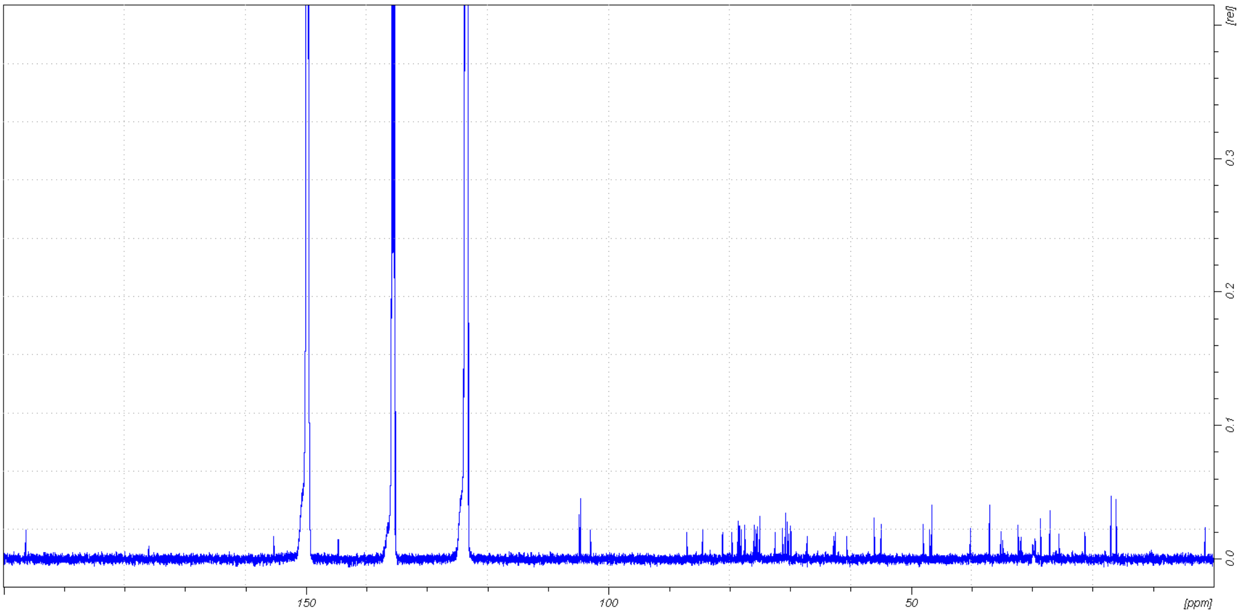

Figure S72. ^{13}C NMR spectrum of **10**

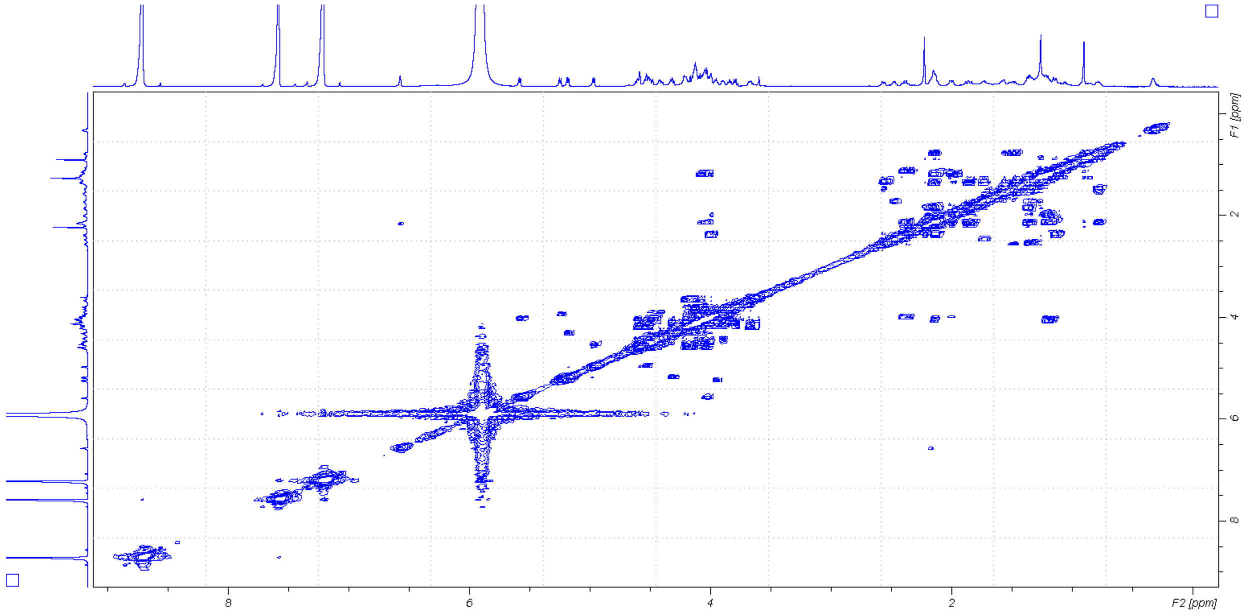

Figure S73. ^{1}H-^{1}H COSY spectrum of **10**

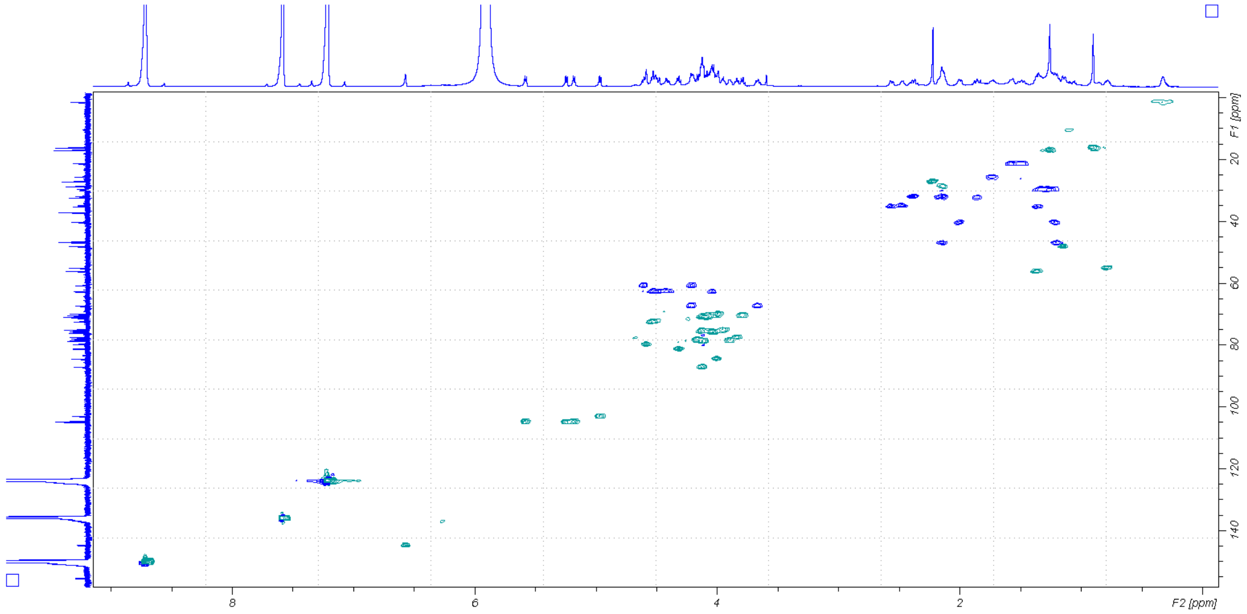

Figure S74. HSQC spectrum of **10**

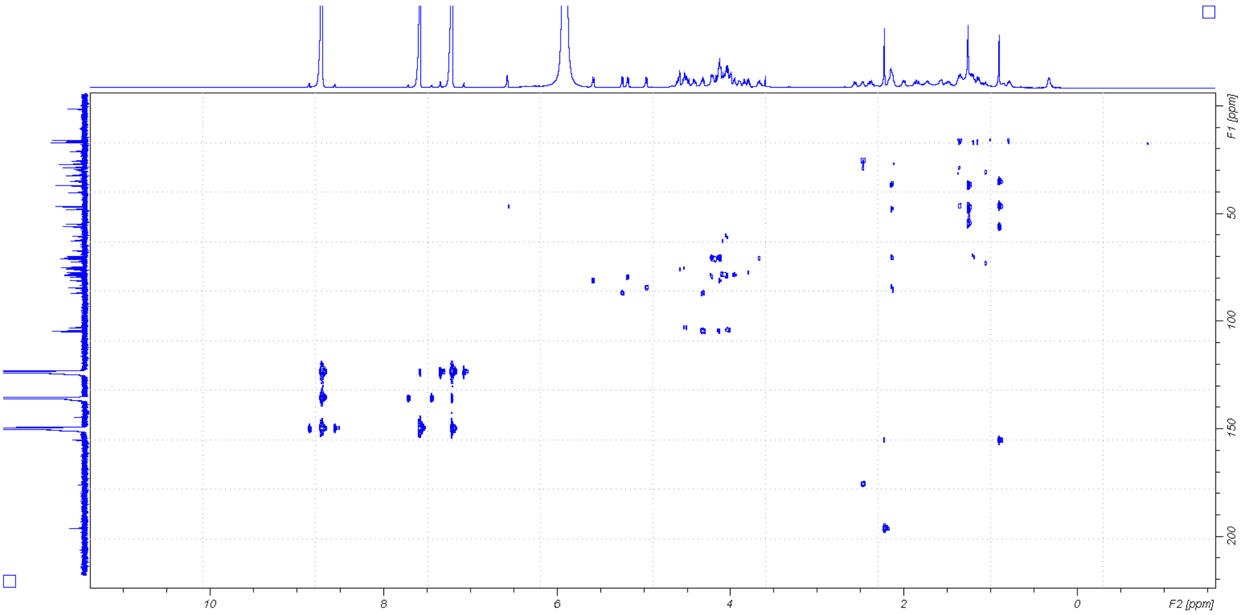

Figure S75. HMBC spectrum of **10**
